# Metabolome Dynamics of Smutted Sugarcane Reveals Mechanisms Involved in Disease Progression and Whip Emission

**DOI:** 10.3389/fpls.2017.00882

**Published:** 2017-05-31

**Authors:** Patricia D. C. Schaker, Leila P. Peters, Thais R. Cataldi, Carlos A. Labate, Camila Caldana, Claudia B. Monteiro-Vitorello

**Affiliations:** ^1^Department of Genetics, “Luiz de Queiroz”' College of Agriculture, University of São PauloSão Paulo, Brazil; ^2^Brazilian Bioethanol Science and Technology LaboratorySão Paulo, Brazil; ^3^Max Planck Partner Group at Brazilian Bioethanol Science and Technology LaboratorySão Paulo, Brazil

**Keywords:** plant–microbe interactions, fungal pathogen, sugarcane smut, metabolomics, meristem

## Abstract

Sugarcane smut disease, caused by the biotrophic fungus *Sporisorium scitamineum*, is characterized by the development of a whip-like structure from the plant meristem. The disease causes negative effects on sucrose accumulation, fiber content and juice quality. The aim of this study was to exam whether the transcriptomic changes already described during the infection of sugarcane by *S. scitamineum* result in changes at the metabolomic level. To address this question, an analysis was conducted during the initial stage of the interaction and through disease progression in a susceptible sugarcane genotype. GC-TOF-MS allowed the identification of 73 primary metabolites. A set of these compounds was quantitatively altered at each analyzed point as compared with healthy plants. The results revealed that energetic pathways and amino acid pools were affected throughout the interaction. Raffinose levels increased shortly after infection but decreased remarkably after whip emission. Changes related to cell wall biosynthesis were characteristic of disease progression and suggested a loosening of its structure to allow whip growth. Lignin biosynthesis related to whip formation may rely on Tyr metabolism through the overexpression of a bifunctional PTAL. The altered levels of Met residues along with overexpression of SAM synthetase and ACC synthase genes suggested a role for ethylene in whip emission. Moreover, unique secondary metabolites antifungal-related were identified using LC-ESI-MS approach, which may have potential biomarker applications. Lastly, a putative toxin was the most important fungal metabolite identified whose role during infection remains to be established.

## Introduction

Sugarcane (*Saccharum* spp.) has long been recognized as one of the world's most efficient crops in converting solar energy into harvestable chemical energy, storing exceptionally high concentrations of sucrose, which can achieve 25% of fresh weight under favorable conditions (Chandra, 2011). Therefore, sugarcane is the main feedstock for sugar and ethanol production in tropical and subtropical countries (Botha and Moore, 2014). The carbon partitioning is directly related to the well-established concept of source (photosynthetic) and sink (non-photosynthetic) tissues systems (McCormick et al., 2009), where sucrose is synthesized in source tissues, transported via phloem and distributed via apoplast (Robinson-Beers and Evert, 1991) and symplast (Rae et al., 2005). Typically, in mature tissues of sugarcane, the carbon skeletons are converted to sucrose and stored in cellular vacuoles, whereas, in younger tissues, they are used for building proteins and synthesizing cell wall fibers (Bindon and Botha, 2002; Rae et al., 2005).

In the sucrose metabolism, carbon is partitioned into several compounds including organic acids, amino acids, proteins, cell wall components and secondary metabolites (Botha and Whittaker, 1997; Wang et al., 2013). In response to pathogen attack, carbon partitioning can be affected by the activation of a wide range of defense mechanisms, which involves the redistribution of energy to the synthesis of secondary metabolites, cell wall reinforcement, production of reactive oxygen species (ROS) and changes in hormonal status (Bolton, 2009). Sugarcane is constantly challenged by biotic stress, which can compromise crop productivity. Sugarcane smut is one of the most important diseases, leading to economic losses due to a reduction in sugar content and juice quality (Croft and Braithwaite, 2006; Sundar et al., 2012). Sugarcane smut is caused by the biotrophic basidiomycete *Sporisorium scitamineum*. The characteristic symptom of the disease is the development of whip-like structure from plant meristems composed of sugarcane tissues surrounded by fungal sporogenesis (Sundar et al., 2012). Smut whips act as a sink tissue and depends on the plant carbon supply for growth (Doidy et al., 2012). Many studies have been carried out to identify the molecular basis of this disease, including changes in gene expression, protein accumulation and specific cell wall components, that can be used as determinants of resistance (Legaz et al., 1998; Piñon et al., 1999; Heinze et al., 2001; Fontaniella et al., 2002; Borrás-Hidalgo et al., 2005; Millanes et al., 2005; Lao et al., 2008; Santiago et al., 2009, 2010, 2012; Que et al., 2011; You-Xiong et al., 2011; Su et al., 2013; Wu et al., 2013; Esh, 2014; Huang et al., 2015; Barnabas et al., 2016; Peters, 2016; Schaker et al., 2016).

Changes in gene expression and/or protein accumulation are not always directly related to the observed biological function and phenotype, which are the result of multiple regulatory interactions (Fiehn et al., 2000). Metabolomics has emerged as a complementary tool to functional genomics with the potential to accelerate the understanding of complex molecular interactions in biological systems (Hall et al., 2002; Jorge et al., 2016). This technique performs one of the highest levels of post-genomic analysis, aiming to quantify compounds of intermediary metabolic pathways (Allwood et al., 2008; Büscher et al., 2009). Plant metabolome analysis is a great challenge because of the dynamic range of possible molecules of varying concentrations, which may include more than 200,000 compounds (Fiehn et al., 2000). The elucidation of this diversity is being achieved with the development and upgrading of analytical methods. Gas chromatography coupled to mass spectrometry (GC–MS) is the most widely accepted analytical method used in plant metabolomics due to its high reproducibility. One major limitation of GC–MS is its restriction in analyzing volatile and thermally stable metabolites or metabolites that can be chemically modified to produce volatile derivatives through derivatization. Liquid chromatography coupled to mass spectrometry (LC–MS) is the most important complementary technology to GC–MS, where thermolabile and high-molecular weight compounds without any derivatization can be analyzed (Jorge et al., 2016).

In plants, metabolomics has been applied to characterize genetically modified varieties and identify responses related to biotic and abiotic stresses (Carreno-Quintero et al., 2013). In plant-pathogen interaction studies, metabolomics can unravel pathways hijacked by pathogens and predict resistance mechanisms (López-Gresa et al., 2010; Allwood et al., 2012; Lee et al., 2016). In sugarcane metabolomics has been used to determine profiles related to sucrose accumulation (Bosch et al., 2003; Glassop et al., 2007); evaluate genotypes with different degrees of susceptibility to orange rust disease (Leme et al., 2014); distinguish between embryogenic and non-embryogenic callus tissue (Mahmud et al., 2015); and explore potential coproducts besides sucrose (Coutinho et al., 2016).

The present study aims to determine changes in sugarcane metabolome in response to *S. scitamineum* colonization throughout disease progression. We used a combination of gas chromatography coupled with mass spectrometry (GC-TOF-MS) and liquid chromatography coupled to electrospray ionization tandem mass spectrometry (LC-ESI-MS/MS) complementary approaches. The results suggested a reprogramming in plant metabolism very early in response to *S. scitamineum* colonization. Over disease progression, the deeper changes are mostly related to cell wall precursors, amino acids, and energetic and phenylpropanoid pathways. The metabolomics analysis helped to better characterize the biological mechanisms involved in smut disease and corroborated previous hypotheses built on transcriptomic data from the same interaction performed in independent experiments.

## Materials and methods

### Ethics statement

*Sporisorium scitamineum* SSC39 teliospores were collected as described by Taniguti et al. (2015). Experiments were performed using the smut susceptible Brazilian commercial variety of sugarcane, “RB925345”. The healthy plants used to conduct the experiments were collected from the experimental station of the Genetics Department at ESALQ-University of São Paulo, Piracicaba, São Paulo, Brazil. No special permits were necessary for the teliospores or cane collection to scientific research.

### Experimental design

*Sporisorium scitamineum* SSC39 teliospores (Taniguti et al., 2015) with viability >95% were used to inoculate single budded setts of 7-month-old plants. Setts were subjected to disinfection following three steps: thermal treatment (30 min in water bath at 52°C; 1 kg of setts per 6 L of water); 10 min in sodium hypochlorite solution (4 mL.L^−1^) and three washes in distilled water. Setts were than inoculated using the puncture method (10^6^ teliospores.mL^−1^ in saline solution; NaCl_2_ 0.85 M) (Taniguti et al., 2015).

Mock-inoculated plants were prepared only with saline solution (control plants). Inoculated and control plants were placed in greenhouse benches in a completely randomized experimental design. The experiment was conducted from February to May of 2015. Plants were irrigated during early morning hours every day and no temperature control was used. Sampling was done from buds 5 days after inoculation (DAI), and the meristematic region of the main culm at 65 DAI, 100 DAI, and 120 DAI. The last corresponded to the time immediately after whip emission. Each time point analyzed was represented by five biological replicates composed of pools of three plants for 5, 65, and 100 DAI samples. The 120 DAI replicates were represented by one plant (Supporting Information Figure S1). All samples were frozen in liquid nitrogen immediately after collection and stored at −80°C. Infected plants were compared to control samples of the same age.

### Quantification of *S. scitamineum* DNA

Real-time qPCR was used to confirm and quantify *S. scitamineum* infection in each biological replicate. CTAB method was used for DNA extraction (Doyle and Doyle, 1990). qPCRs used as target the ribosomal Intergenic Spacer region (IGS) from *S. scitamineum* genome (Peters, 2016). Reactions consisted of 100 ng of total DNA, 0.2 μM of each primer, and 1 × LuminoCt SYBR Green qPCR ReadyMix (Sigma-Aldrich), in a total volume of 12.5 μL. Cycling parameters were 95°C for 20 s, followed by 40 cycles of 95°C for 3 s and 60°C for 30 s. All reactions were performed in an ABI 7500 Fast real-time PCR detection system (Applied Biosystems) using technical duplicates. The quantity of *S. scitamineum* DNA in each sample was determined by absolute quantification based on a standard curve obtained using DNA extracted from mixed cultures of *S. scitamineum* SSC39A and SSC39B isolates. Quantifications were statistically analyzed using *t*-test (*p* < 0.05).

### GC-TOF-MS analysis and data processing

Frozen samples collected of each time point were grounded manually with liquid nitrogen using sterilized mortar and pestle. Metabolites were extracted from 25 mg of freshly ground material. Extraction solution was composed of methanol, chloroform and water (3:1:1) as described by Gullberg et al. (2004). The isotopically labeled succinic acid (D4, 98%-DLM 584-5), myristic acid (1, 2, 3-13C3, 99%-CLM 3665-0.5) and palmitic acid (1, 2, 3, 4-13C4) were used as internal standards. Metabolites extraction followed the protocol of De Vos et al. (2007) with minor modifications. Initially, 0.5 mL of cold extraction solution was added in each sample along with tungsten magnetic beads, and subjected to agitation in Vibration Mill (Retsch) for 30 s and 20 Hz. Beads were removed and samples sonicated for 15 min at 4°C and centrifuged for 10 min at 4°C, 16,000 g. The supernatant was filtered (Millex 0.22 μM filter, Millipore) and stored at −80°C.

One hundred μl (100 μL) of the organic phase was dried and derivatized as described in Roessner et al. (2001). One μL of the derivatized samples were analyzed on a Combi-PAL autosampler (Agilent Technologies GmbH, Waldbronn, Germany) coupled to an Agilent 7890 gas chromatograph coupled to a Leco Pegasus 2 time-of-flight mass spectrometer (LECO, St. Joseph, MI, USA) in split (1:40) and splitless mode described by Weckwerth et al. (2004). Chromatograms were exported from Leco ChromaTOF software (version 3.25) to R software. Peak detection, retention time alignment, and library matching were performed using Target Search R-package (Cuadros-Inostroza et al., 2009).

Metabolites were quantified by the peak intensity of a selective mass. Metabolites intensities were normalized by dividing the fresh weight of each biological replicate, followed by the sum of total ion count (TIC) and Log2 transformed. Metabolite data were normalized by dividing each raw value by the median of all measurements of the experiment for one metabolite. The significance of metabolites was tested by *t*-test (*p* < 0.05).

### LC-ESI-MS/MS analysis and data processing

Frozen samples were grounded as described before. Metabolites were extracted from 25 mg of freshly ground material following the protocol of De Vos et al. (2007) with a solution composed of 99.875% of methanol and 0.125% of formic acidand 50 pmol of quercetin as internal standard. Metabolites were also analyzed in a mass spectrometer Q-TOF Ultima-API (Waters), with ESI ionization source (Electrospray Ionization), coupled to an Acquity UPLC (Waters). For UPLC chromatographic separation 5 μL of sample was injected into a reversed phase column (C18 100 × 2.1 mm 1.7 μM Acquity-Waters). Two eluents were used as the mobile phase: A (100% water containing 0.1% formic acid) and B (100% acetonitrile containing 0.1% formic acid). The mobile phase gradient used was: 95% A and 5% B for 6 min, 25% A and 75% B for 6 min, 5% A, and 95% B for 1 min. The capillary voltage was 3 kV and cone voltage of 35 kV. The temperature in the ionization source was 150°C, and the desolvation temperature was 450°C. The nitrogen flow was 50 L.h^−1^ in the cone and 550 L.h^−1^ at the source. Data were acquired in positive and negative mode and centroid acquisition in the mass dynamic range of m/z 100 to 1,000 using MassLynx 4.1 software.

The raw data were processed in MarkerLynx v 4.1 (Waters) for alignment, noise removal, deconvolution, normalization using the quercetin *TIC* (*Total Ion Counts*) and obtaining the intensity of each possible metabolite, using 250 intensity as the lower limit (threshold). Then the intensity of each metabolite was normalized by fresh weight (mg) of the corresponding sample.

Data processing was performed with Metaboanalyst 3.0 software (Xia et al., 2015). First, data were filtered using interquartile range, log transformed and normalized using Pareto scaling. Partial least squares discriminant analysis (PLS-DA) was applied to the metabolite dataset from the two subject classes (control and infected plants) of each time analyzed. PLS-DA was validated by leave-one-out cross-validation. MetaboAnalyst 3.0 software was also used to generate the Variable Importance on Projection (VIP) scores, which identifies the best variables for discriminating between subject classes. Differential accumulation of metabolites was determined using a *t*-test between infected and control samples of the same age. *P*-values were corrected using the Benjamini-Hochberg method and a cut-off of FDR < 0.05 was applied. Top 10 VIPs released from LC-ESI-MS analysis of each comparison were selected for fragmentation. LC-ESI-MS/MS analyses were conducted in the same ionization conditions described above, and fragmentation was performed using collision energy values ranging from 10 to 40 eV. Fragmentation data was compared to the Metlin database (https://metlin.scripps.edu/index.php) to find possible metabolites, and ACD/MS Structure ID suite software was used to compare fragmentation profiles to theoretically fragmented metabolites from the database and manually checked.

### RT-qPCR

To further investigate plant responses to smut, gene expression analysis of key sugarcane genes was performed (Supporting Information Table S1). Primers were manually designed and quality verified using Gene Runner (http://www.generunner.net/) and NetPrimer (http://www.premierbiosoft.com/netprimer/) software. All RT-qPCRs were conducted on the 7500 Fast Real-Time PCR System (Applied Biosystems) using GoTaq® One-Step RT-qPCR System Kit (Promega). A reaction mixture containing 50 ng of RNA, 1 X of GoTaq® qPCRMaster Mix, 0.2 μM of each primer, 1 X μL of GoScript™ RT Mix and nuclease-free water to a final volume of 12.5 μL was prepared. Five biological and two technical replicates were used. Cycling conditions were as follows: 37°C for 15 min, 95°C for 10 min, 40 cycles of 95°C for 10 s, 60°C for 30 s, and 72°C for 30 s. Primer specificity was confirmed obtaining the dissociation curve for every reaction. Sugarcane genes encoding for polyubiquitin (Papini-Terzi, 2005) and GAPDH (Iskandar et al., 2004) were used to normalize expression signals. PCR efficiencies and Cq values were obtained using the LinReg PCR program (Ramakers et al., 2003). Relative changes in gene expression ratios were calculated by REST software (Pfaffl et al., 2002). Control samples (mock-inoculated plants) were used as calibrators. The *t*-test was used to estimate significant changes in the relative expression levels (*p* < 0.05).

### Starch staining

Starch accumulation was examined in fresh cuts of sugarcane infected plants after whip development. Samples obtained from the whip region with intense sporulation, whip base, primary meristem and stem were stained using the potassium iodide-iodine reaction (I_2_KI) for 5 min and observed under Light microscopy (Optika B-350 microscope). The images of I_2_KI stained preparations were collected using an Optikam B5 digital camera.

### Phenylalanine/tyrosine ammonia-lyase phylogeny

The accession number of amino acid sequences used in the phylogenetic analysis is presented in Supporting Information File S1. Multiple sequence alignments were constructed using ClustalW implemented in MEGA 6.06 Software (Tamura et al., 2013) with default settings. Maximum-likelihood algorithm was used to build the phylogenetic tree with the following settings: JTT model, 1000 replicates of bootstrap analyses, with the best network-network interface (NNI) topology search.

### Identification of *S. scitamineum* gene clusters involved in secondary metabolites biosynthesis

The complete genome of *S. scitamineum* SSC39B strain (Taniguti et al., 2015) was analyzed using antiSMASH 3.0 software (Weber et al., 2015) with default parameters to identify gene clusters related to secondary metabolites biosynthesis. The gene expression profiles of each identified cluster were obtained from RNAseq data of pathogen growth *in vitro* and *in planta* (5 DAI and after whip development) using the normalized number of reads (Taniguti et al., 2015).

## Results

### Pathogen growth within plant tissues

The meristem region was analyzed at four time points during sugarcane-smut interaction: two representing the limits of the colonization process (5 and 120 DAI), as studied in previous experiments (Taniguti et al., 2015; Schaker et al., 2016); and two representing intermediate steps of the infection process (65 and 100 DAI). This design was used to further explore the molecular events previously described at transcriptional and proteomic levels (Barnabas et al., 2016; Schaker et al., 2016), and narrow down time points that can reveal the host candidate molecules that potentially influence fungal sporogenesis and whip development and/or fungal molecules acting to suppress plant defenses and complete its life cycle.

**Figure 1:**
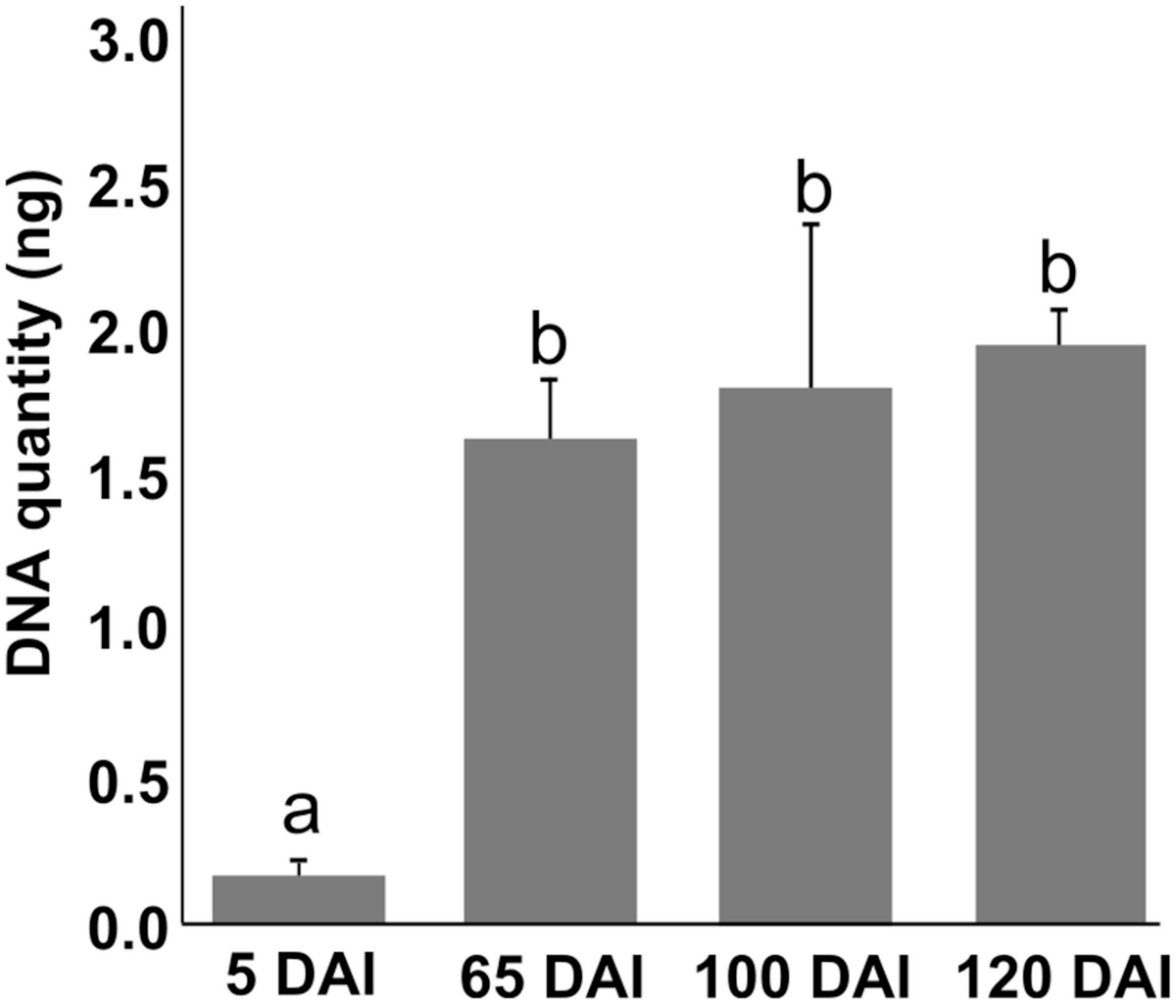
*****Sporisorium scitamineum*** DNA quantification assay in sugarcane shoot apical meristem region**. Real-time qPCR analysis in samples used to obtain metabolomic data. Quantities are relative to 100 ng of total DNA. “a” and “b” represent statistical significance (*t*-test, *p* < 0.05). Standard curves and amplification efficiency were used as described by Peters (2016).

Growing concentrations of the pathogen from 5 to 65 DAI were observed in inoculated plants using qPCR with DNA from the same samples used in metabolomic analysis. However, fungal concentration later than 65 DAI remained constant, indicating that concentrations of the pathogen over time is not determinant of whip emission (Figure 1).

### Major impacts on the primary metabolism of smut-infected plants

**Figure 2:**
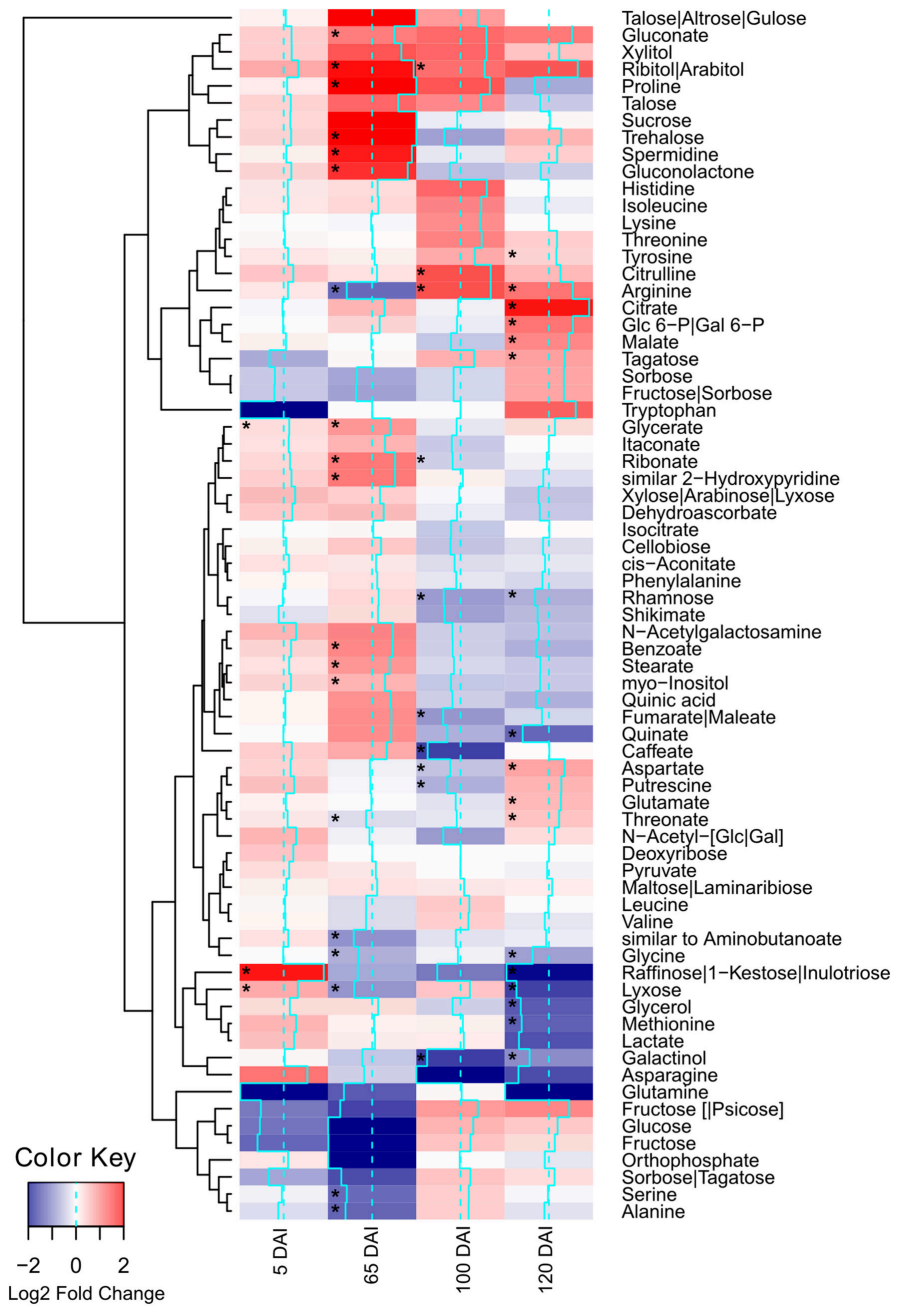
**Metabolites of sugarcane shoot apical meristem region identified in GC-TOF-MS analysis**. Heatmap was built using Log2 Fold Change (Inoculated/Control) of relativized medians using gplots (Warnes et al., 2013) for R (R Core Team, 2015). Blue scale indicates low concentration in infected samples, and red scale indicates high concentration in infected samples. Squares “^*^” marked represent metabolites showing statistical significance comparing infected vs. control (*t*-test, *p* < 0.05).

Using GC-TOF-MS, 73 primary metabolites, including organic acids, amino acids, mono- and di-saccharides, were identified in the primary sugarcane meristem (Supporting Information Table S2). Each time point analyzed presented a particular set of compounds quantitatively altered by infection as compared with healthy plants of the same age (*p* < 0.05) (Figure 2, Supporting Information Table S2). The overall picture reflected that carbon partitioning is largely affected during fungal colonization and whip development disturbing the normal source to sink dynamics throughout the course of infection. Additionally, a markedly shift in plant meristem metabolism occurs between 65 and 100 DAI.

### Early events in the sugarcane-smut affected mostly sugar accumulation

Soon after inoculation (5 DAI), the infected plants accumulated significantly (*p* < 0.05) higher levels of glycerate, lyxose and raffinose. The raffinose levels were approximately 10 times higher in infected samples (Figure 2), suggesting an important role of this sugar during pathogen recognition. However, raffinose levels significantly decreased close to sporogenesis and whip development.

### Amino acids differential accumulation

Changes in amino acid accumulation were remarkable in infected plants (Figure 2, Supporting Information Figure S2). The more accentuated changes were detected at 65 DAI when proline levels (Pro) were significantly increased and serine (Ser), alanine (Ala), arginine (Arg), and glycine (Gly) were reduced in infected plants (*p* < 0.05).

Before whip development, at 100 DAI, Arg and aspartate (Asp) levels differed in infected plants. Both amino acids were also affected after whip emission (120 DAI), i.e., Arg levels remained significantly higher in the infected samples, along with glutamate (Glu), while Asp and Gly levels were reduced. Whip emission was also characterized by increased tyrosine (Tyr) that may be related to the phenylpropanoid pathway. It was also observed reduction in methionine residues (Met) suggesting its involvement in ethylene synthesis as previously hypothesized, in which genes encoding ACC and SAM synthases were up regulated at this same time point (Schaker et al., 2016).

### Carbon partitioning

**Figure 3:**
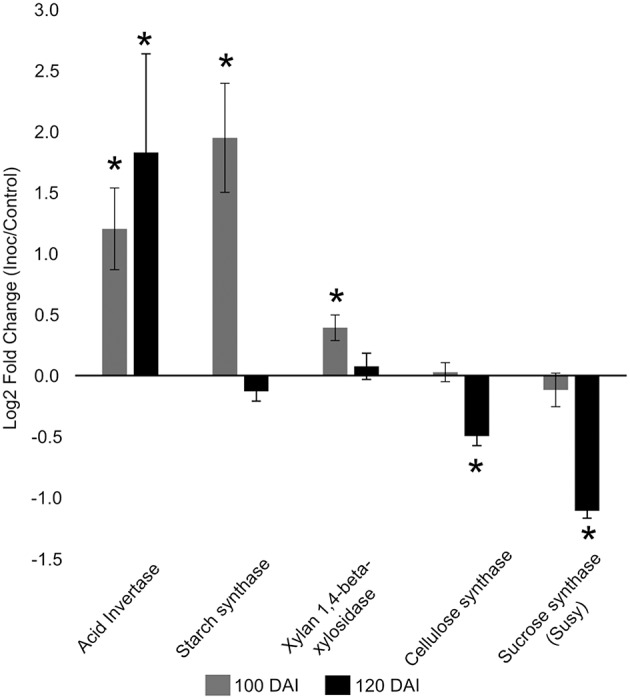
**Gene expression profiles in sugarcane shoot apical meristem region**. RT-qPCR analysis of key sugarcane genes related to smut disease. “^*^”: indicates significant changes of transcripts in infected plants compared to control ones (*t*-test, *p* = 0.05). Genes: acid invertase, starch synthase, xylan 1,4 beta xylosidase, cellulose synthase, sucrose synthase. Data are presented as Log2 Fold Change of infected/control samples.

**Figure 4:**
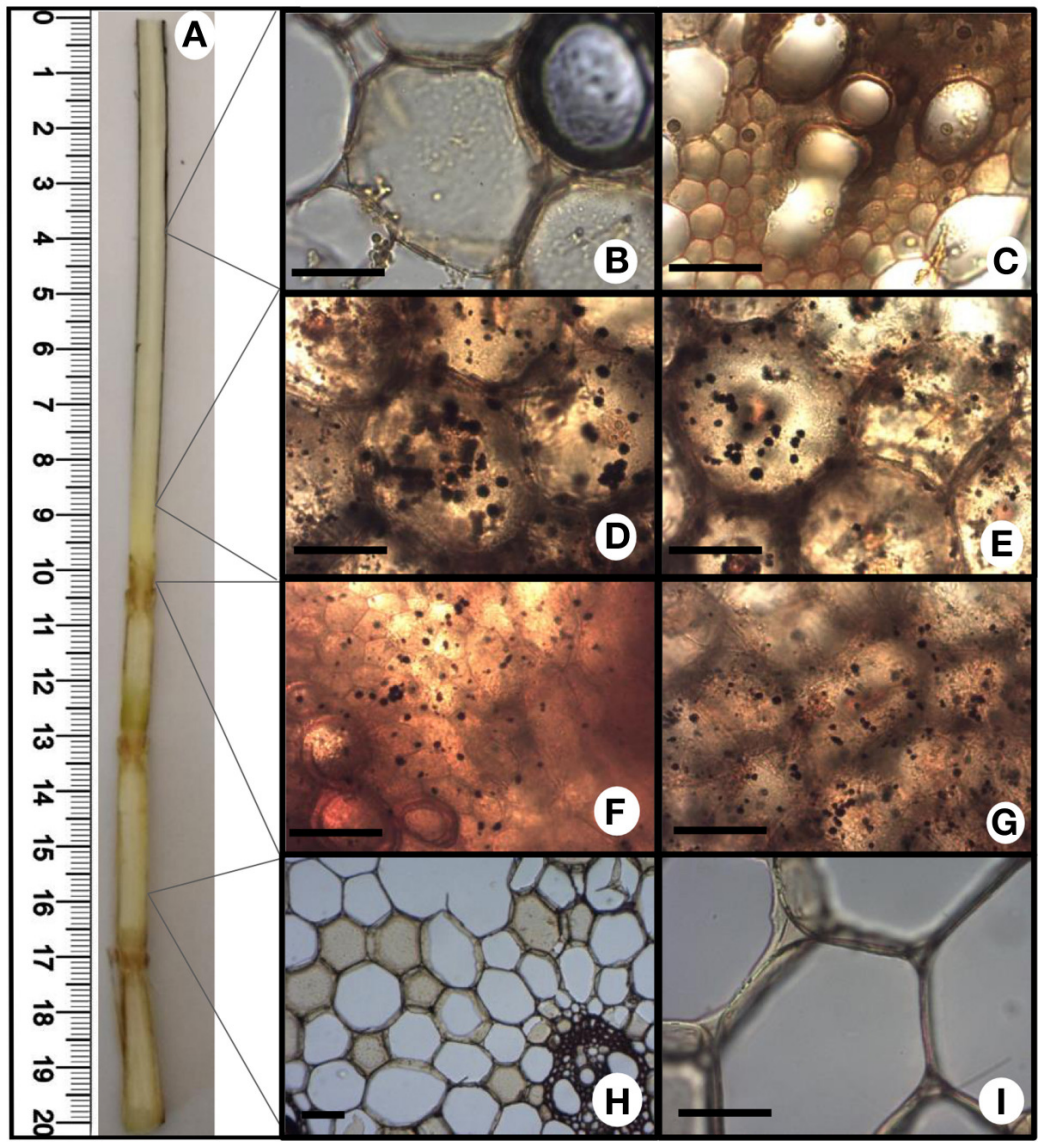
**Starch accumulation after whip emission**. Potassium iodide-iodine reaction (I_2_KI) staining was used to detect starch by light microscopy in different fresh sections of sugarcane tissues. **(A)** Internal view of sugarcane whip; **(B,C)** whip region with intense sporulation; **(D,E)** basis of whip; **(F,G)** primary meristem; **(H,I)** stem. Scale bars = 10 μm.

The increased energy requirements of infected plants was evident, even before whip emission, from the accumulation of the intermediates of glycolysis, tricarboxylic acid cycle (TCA) and pentose phosphate pathway (PPP). This metabolic response can provoke negative impacts in stem sucrose accumulation, despite whip emission or delay, even though the sucrose level was not altered in the meristem of infected plants. After whip emission, significantly increased levels of glucose 6P, malate and citrate were detected (Figure 2). These changes may also be correlated to increased gene expression of soluble acid invertase detected in 100 and 120 DAI samples (Figure 3). At 100 DAI, the increased expression of starch synthase gene (Figure 3) suggests starch accumulated after whip emission at the meristem and at the whip base as shown in Figure 4.

Changes in the levels of other carbohydrates may contribute to signaling besides differential carbon allocation. For instance, trehalose levels had a subtle increase in infected samples at 65 DAI and higher levels of ribitol/arabitol were found in infected samples at 65 and 100 DAI. Arabitol is synthesized by reduction of either arabinose or lyxose, which might explain the significantly low levels of lyxose in infected plants 65 DAI. After whip emission, raffinose was significantly reduced in infected plants together with its precursor galactinol, which exhibited reduced levels in 100 and 120 DAI samples.

### Cell wall precursors

Several metabolites related to cell wall biogenesis were identified in the GC-TOF-MS analysis, including shikimate, phenylalanine, tyrosine, xylose, rhamnose, cellobiose, and caffeate. Most of them presented a similar pattern of distribution in the late stages of the smut disease-100 and 120 DAI (Figure 2). There was a significant reduction in rhamnose and caffeate at 100 DAI, and rhamnose after whip emission, suggesting cell wall weakening may allow whip growth or that cell wall precursors are being redirected to feed whip growth.

The gene expression analysis (RT-qPCR) of the following sugarcane genes: xylan 1,4-beta-xylosidase, cellulose synthase and sucrose synthase was performed to further investigate plant responses related to whip development and cell wall constitution (Figure 3). Cell wall weakening may be related to the increased expression of hemicellulose degrading xylan 1,4-beta-xylosidase before whip development. Cellulose synthase and sucrose synthase expression did not change before whip development but showed a significant reduction (*p* < 0.05) at 120 DAI–after whip emission (Figure 3).

**Figure 5:**
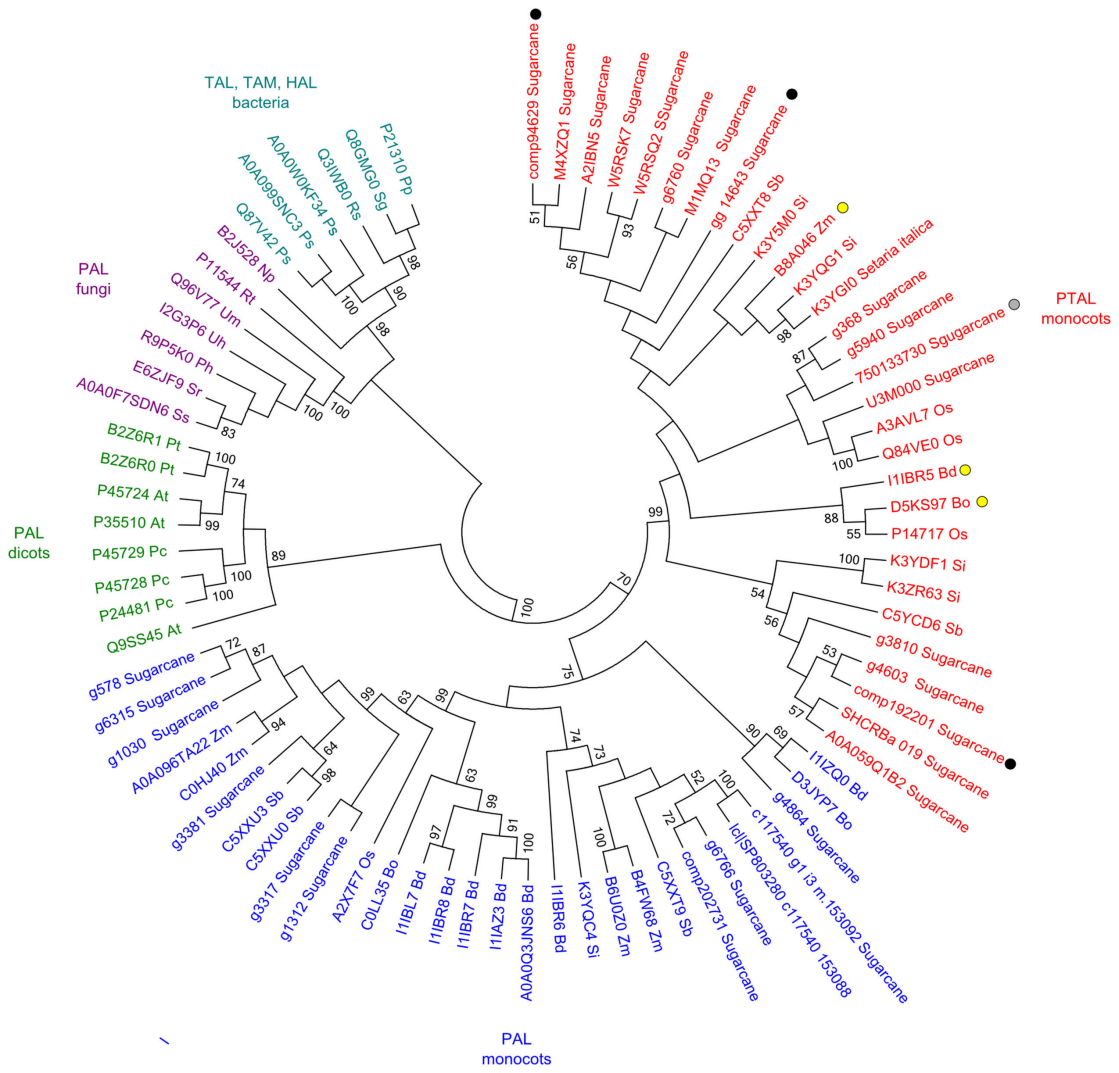
**Sequence analysis of PAL and PTAL proteins**. Phylogenetic tree of PTAL and PAL in plants and fungi, and TAL, tyrosine ammonia-mutase (TAM) and histidine ammonia-lyase (HAL) in bacteria. Multiple sequence alignments obtained in ClustalW implemented in MEGA 6.06 Software (Tamura et al., 2013) with default settings. Maximum-likelihood algorithm was used to build the phylogenetic tree with the following settings: JTT model, 1,000 replicates of bootstrap analyses, with the best network-network interface (NNI) topology search. Protein sequences were obtained from Uniprot and GenBank (Supporting Information File S1). Black circles: sugarcane genes encoding the enzymes up-regulated in plants after whip development (Schaker et al., 2016); Gray circle: sugarcane protein identified exclusively in sugarcane plants after whip development (Barnabas et al., 2016); Yellow circles: previously reported PTAL proteins (Barros et al., 2016; Maeda, 2016).

Higher levels of tyrosine in the infected samples suggested that the phenylpropanoid pathway is affected as previously detected (Barnabas et al., 2016). Although several studies inferred the responses of PAL (phenylalanine ammonia-lyase) in smut infected plants, the changes in Phe levels were not observed in our study. Considering that in grasses, lignin is partially synthesized from tyrosine by PTAL, a bifunctional phenylalanine-tyrosine ammonia-lyase, we analyzed the available amino acid sequences of sugarcane PAL and PTAL, and found that proteins encoded by genes responsive to smut in transcriptome analysis and proteins detected in proteomic assays are from the PTAL family (Figure 5), suggesting that Tyr accumulation is indeed related to increased levels of lignin in smut infected samples.

### Major responses related to secondary metabolism during smut-cane interaction

**Figure 6:**
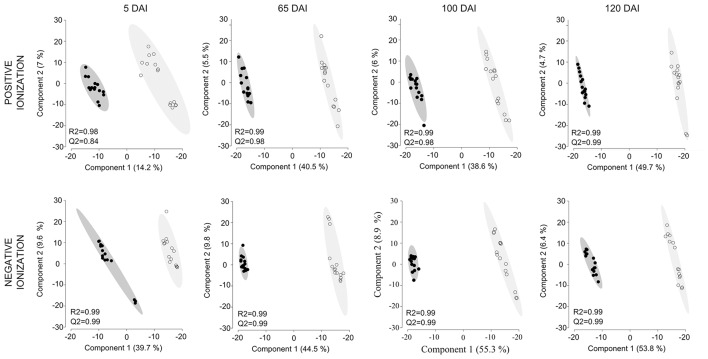
**Secondary metabolites differentiating infected and non-infected plants in sugarcane shoot apical meristem region**. PLS-DA plots obtained in Metaboanalyst software using LC-ESI-MS metabolome profile from positive and negative ionization in 5, 65, 100, and 120 DAI samples. Black dots represent biological and technical replicates of control plants, empty dots represent infected plants. Ellipses indicates the 95% confidence region. R2 and Q2 values obtained by cross-validation using three components.

Untargeted LC-ESI-MS was implemented as a complementary approach for describing the changes in secondary metabolism during the smut-sugarcane interaction. Ionization in positive mode detected 254, 216, 262, and 260 non-redundant *m/z* in samples from 5, 65, 100, and 120 DAI, respectively (Table 1), while negative ionization resulted in 290, 223, 235, and 232 non-redundant *m/z* detected for the same samples (Table 1). PLS-DA plots (Figure 6) highlighted the distinction between control and infected plants considering 95% of the level of confidence. In 5 DAI samples, 33 and 83 metabolites were quantitatively altered during the interaction (FDR ≤ 0.05) using positive and negative ionization, respectively. These numbers are increased with disease progression (Table 1), indicating that changes in secondary metabolites composition after whip emission were relevant but poorly understood.

**Table 1:** **Number of non-redundant ***m/z*** detected using LC-ESI-MS analysis and number of those differentially accumulated (FDR ≤ 0.05) comparing inoculated and control samples of the same age**.

**Sample**	**Positive Ionization**		**Negative Ionization**	
	**Total number of m/z detected**	**m/z regulated (FDR < 0.05)**	**Total number of m/z detected**	**m/z regulated (FDR < 0.05)**
5 DAI	254	33	290	83
65 DAI	216	107	223	87
100 DAI	262	113	235	137
120 DAI	260	154	232	173

**Figure 7:**
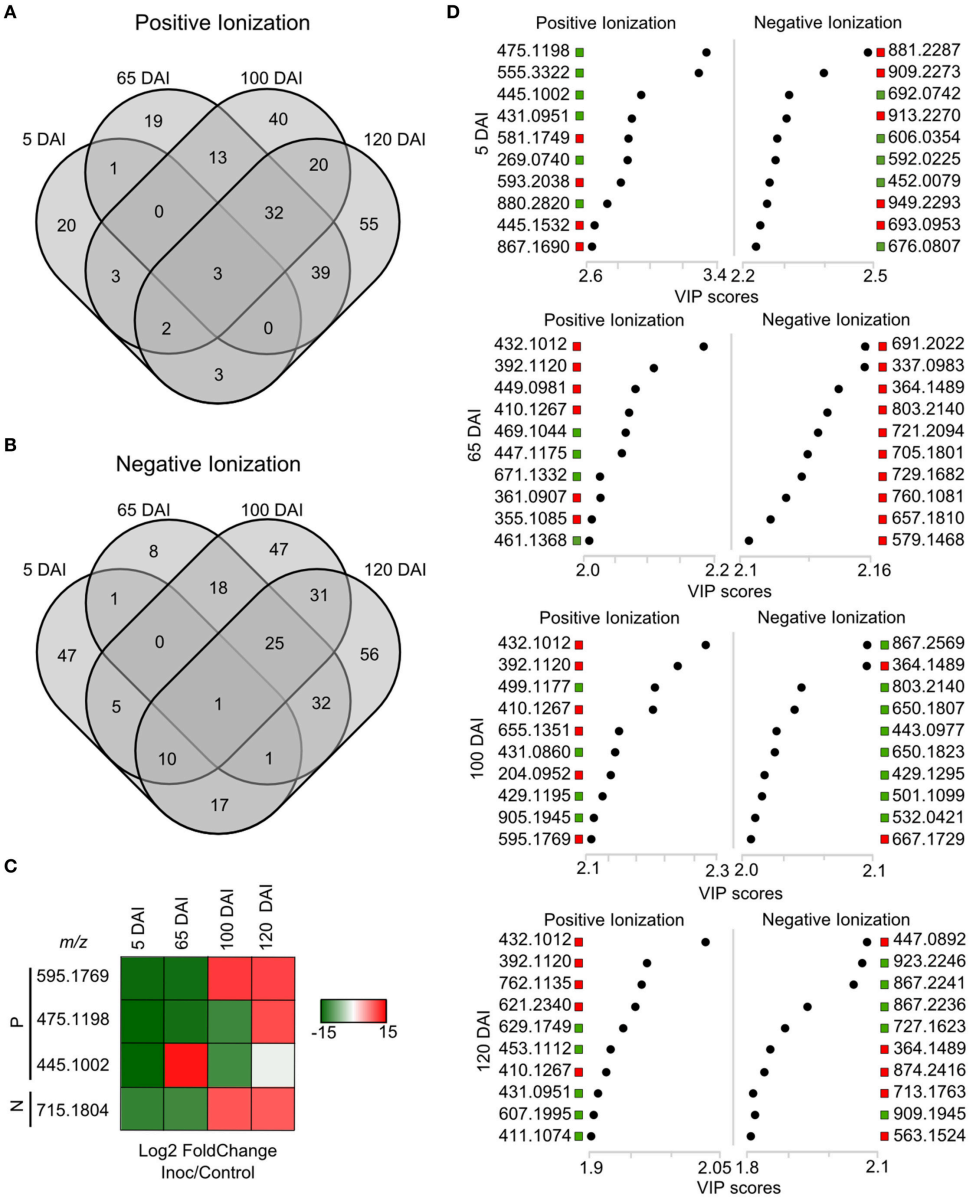
**LC-ESI-MS metabolomics analysis in sugarcane-smut interaction**. Four-way Venn diagram representing differentially accumulated (FDR ≤ 0.05) non-redundant *m/z* of each time point analyzed among infected and control plants of the same age using **(A)** positive and **(B)** negative ionizations. **(C)** Heatmap shows the dynamics of these metabolites during the progression of sugarcane smut disease. It was obtained with gplots (Warnes et al., 2013) for R (R Core Team, 2015) with Log2 Fold Change (inoculated/control) values of shared differentially accumulated *m/z* from Venn diagrams. “P”: Positive ionization, “N”: negative ionization. **(D)** Top 10 *m/z* based on VIP scores from PLS-DA for each time point analyzed in positive and negative ionization. These *m/z* were submitted to LC-ESI-MS/MS analysis to confirm their identity. The x-axis shows the correlation scores and y-axis corresponds to LC-ESI-MS *m/z*. Distribution of each *m/z* in inoculated plants compared to controls is represented as colored squares. Red squares represent those exclusively identified in inoculated samples, green squares represent *m/z* identified exclusively identified in control samples.

Using a set of differentially accumulated *m/z* (FDR < 0.05) detected in LC-ESI-MS approach, four-way Venn diagrams were constructed, which showed that four molecules (*m/z*) were common among all time points analyzed in sugarcane-smut interaction (Figures 7A–C). MS-MS fragmentation allowed the identification of two of these metabolites (Supporting Information File S2): *m/z* 475.1191, with a fragmentation pattern of apigenin 7-O-(6″-O-acetylglucoside), and *m/z* 445.1002, identified as 3′-O-methylderhamnosylmaysin. Apigenin 7-O-(6″-O-acetylglucoside) synthesis was suppressed in the infected plants during disease progression, and increased after whip development, becoming an important marker metabolite related to healthy sugarcane plants.

**Figure 8:**
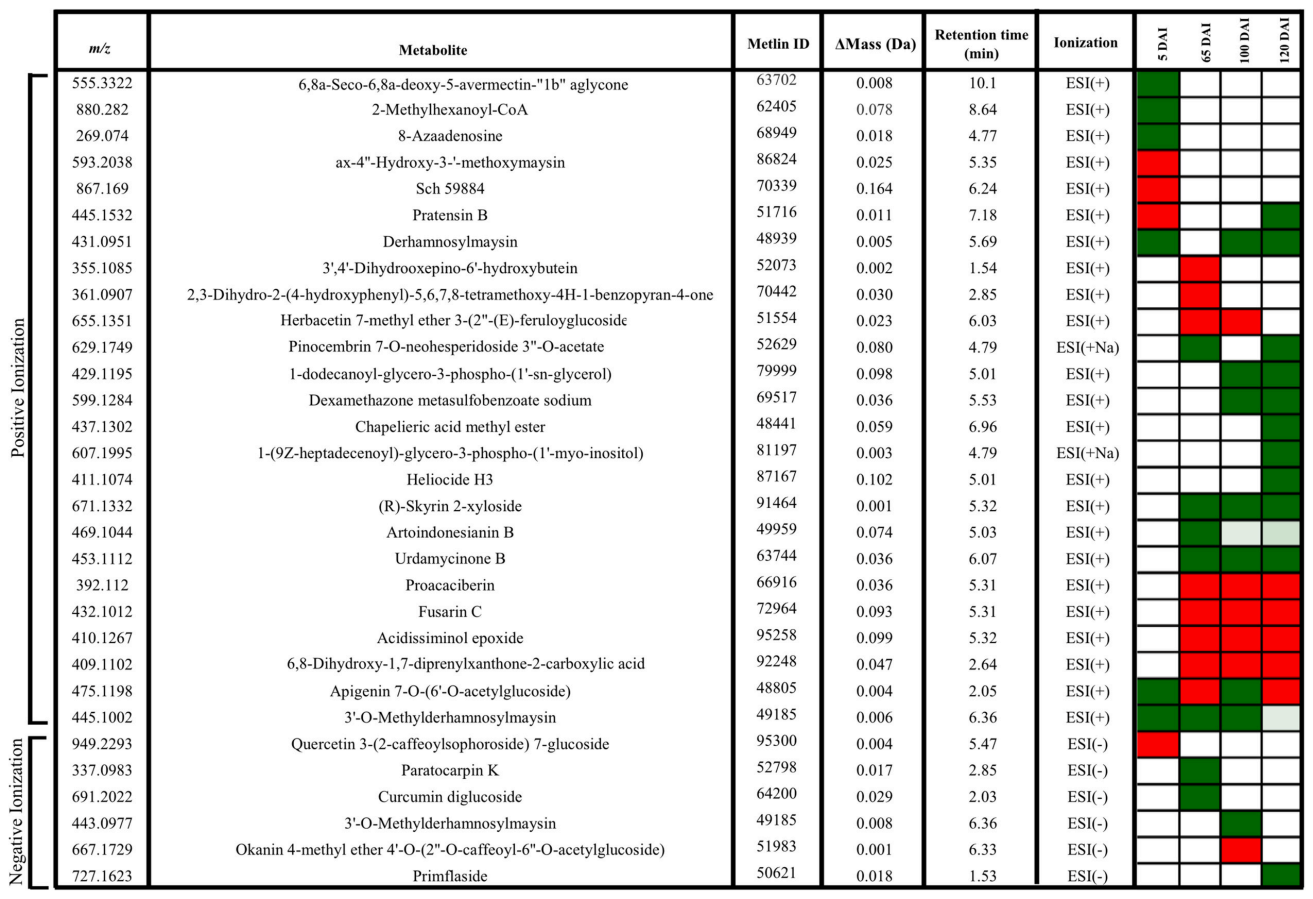
**Metabolites of sugarcane shoot apical meristem region identified in LC-ESI-MS/MS analysis**. ACD/Labs software was used to theoretical fragmentation of structures from Metlin database (https://metlin.scripps.edu/index.php). Green filled squares represent metabolites detected only in control samples; red filled squares represent metabolites identified only in infected samples.

VIP scores (Figure 7D) were used to identify metabolites that most contributed for distinguishing between control and inoculated plants of the same age. The top 10 VIPs from each comparison were fragmented leading to the identification of 25 metabolites from positive ionization and 6 from negative ionization. One metabolite was detected in both ionization methods among the VIPs (Figure 8, Supporting Informations Files S2, S3).

In early infection (5 DAI), pratensin B, ax-4′-hydroxy-3′-methoxymaysin, quercetin 3-(2-caffeoylsophoroside) 7-glucoside and a metabolite with fragmentation pattern similar to the antifungal compound *Sch59884* were detected only in the infected plants, indicating the activation of defense responses. However, the other metabolites were detected only in control samples, for example, 5-oxoavermectin, 2-methylhexanoyl-CoA and 8-azaadenosine. Similarly, the metabolites related to maysin, such as 3′-O-methylderhamnosylmaysin and derhamnosylmaysin were detected only in control samples at 5, 65, and 100 and 5, 100, and 120 days of plant growth, respectively (Figure 8).

The metabolites corresponding to *m/z* 392.1120, 432.1012, 410.1267, and 409.1102 were detected only in infected plants at 65, 100, and 120 DAI. One of them had a fragmentation pattern similar to Fusarin C, a fungal origin metabolite classified as a mycotoxin. Two other identified metabolites (*m/z* 671.1332–R-Skyrin 2-xyloside, and *m/z* 453.1112–urdamycione B) were detected only in control samples at 65, 100, and 120 days of plant growth. After whip emission, the other metabolites were detected only in control samples, including glycero-3-phospho-(1′-myo-inositol), heliocide and primflaside (Figure 8).

## Discussion

Metabolomics is recognized as a powerful tool to describe plant responses to several stimuli. Integration of GC-TOF-MS and LC-ESI-MS/MS complementary tools allowed us to identify a set of metabolites involved in several aspects of plant growth and signaling in the meristem of smutted plants. These metabolic responses were initiated shortly after inoculation and continued past whip emission.

The sugarcane-smut interaction at 5 DAI was characterized by the presence of compounds structurally similar to those with antifungal activities, and by increased levels of raffinose. Raffinose is synthesized from the conjugation of galactinol and sucrose (Sengupta et al., 2015). The enzyme galactinol synthase (GolS; EC 2.4.1.123) is a key component in galactinol production, and its overexpression is correlated with increased resistance to pathogens (Kim et al., 2008; Cho et al., 2010). External application of galactinol in tobacco leads to the expression of genes encoding PR1a, PR1b, and NtACS1, which are well-known defense-related proteins (Kim et al., 2008). Our previous sugarcane-smut transcriptomic data did not detect changes in *gol*S gene expression but instead revealed the repression of an α-galactosidase gene expression (EC 3.2.1.22) (Schaker et al., 2016). The enzyme encoded by this gene is involved in raffinose breakdown, which may be the origin of raffinose accumulation in infected plants. Studies on the differential accumulation of proteins in early smut infection also showed reduced levels of α-galactosidase in both resistant and susceptible genotypes, indicating that this is maybe general response to smut (Su et al., 2016).

Raffinose accumulation has been associated with the response to oxidative burst, which may function as a scavenger of ROS (Van den Ende, 2013; Sengupta et al., 2015) and cell membrane stabilizing compound in unfavorable conditions (Hincha et al., 2003). Sugarcane is known to promote oxidative burst in response to smut infection (Lao et al., 2008; Su et al., 2014; Peters et al., 2017). Recently, detailed analysis of ROS metabolism revealed that a sugarcane resistant genotype maintained high levels of oxygen peroxide by a SOD independent pathway potentially to activate defense responses (Peters et al., 2017). Raffinose accumulation is an attractive candidate strategy together with the antioxidant system to restrain plant cell damage. Peters et al. (2017) did not detect membrane damage in their experiments in any of the genotypes analyzed (susceptible and resistant).

**Figure 9:**
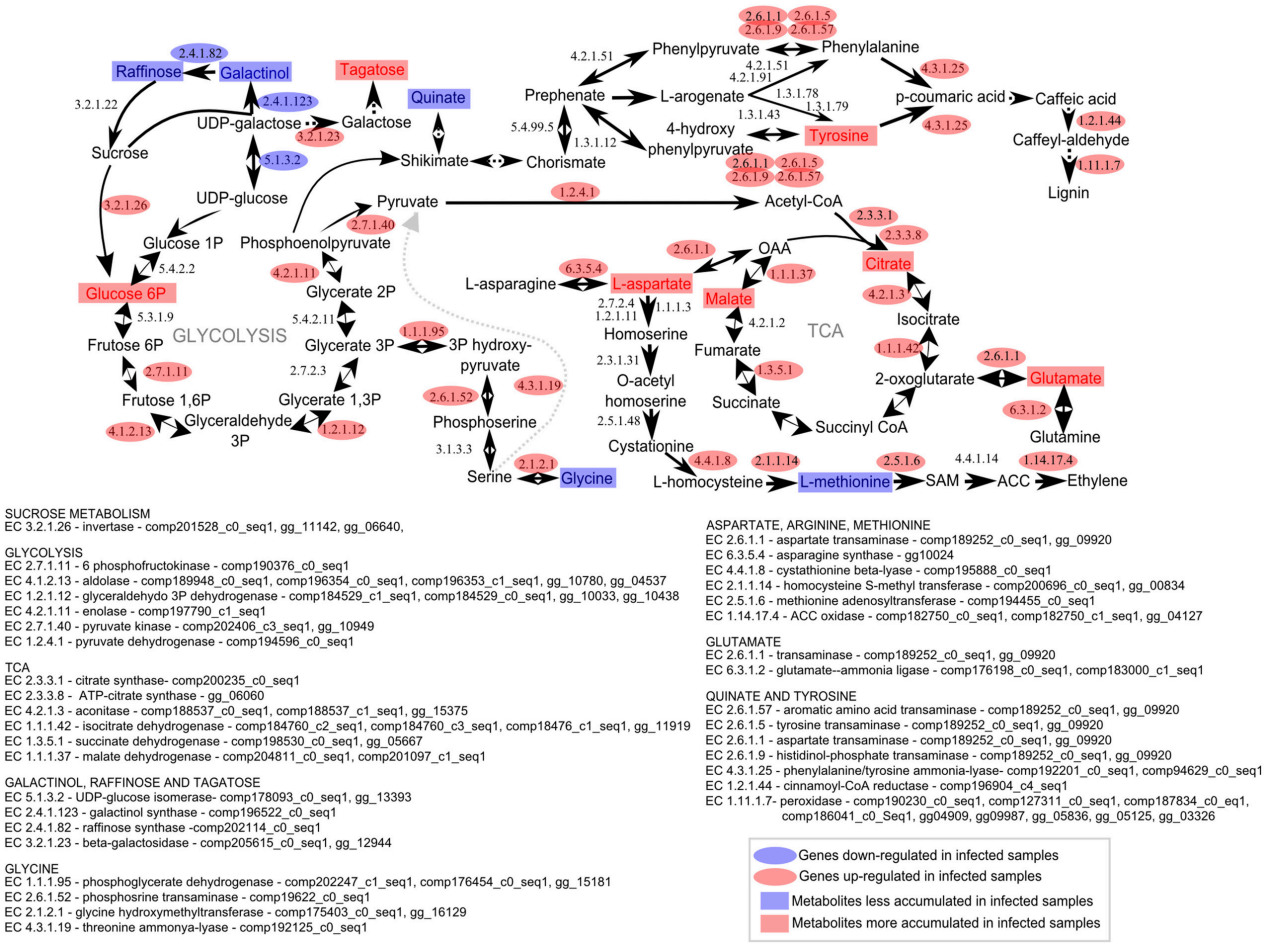
**Metabolomic and transcriptomic responses in sugarcane shoot apical meristem region related to whip emission 120 DAI of ***S. scitamineum*****. Transcriptomic data were obtained from a previous experiment using the same sugarcane genotype and experimental design (Schaker et al., 2016). Rectangles represent metabolites and ovals represent enzyme-encoded by genes detected previously (Schaker et al., 2016) within the same metabolic pathway. Red and Blue represent up or down regulation in infected plants, respectively.

We also speculated that the raffinose pattern of accumulation earlier in infected plants may impair defense signaling in this susceptible genotype. Usually, raffinose does not accumulate in meristem regions. The most abundant sugars in meristem are glucose and fructose (Glassop et al., 2007). Raffinose is often detected in more mature tissues positively associated with sucrose levels. Also, soluble sugar raffinose may accumulate system-wide suggesting their function as transportable stress signals in biotic stresses (Moghaddam and Van Den Ende, 2012). Interestingly, the intermediates of the raffinose-related biosynthesis were inhibited 100 DAI and after whip development. These results corroborated the transcriptomic analysis data (Figure 9).

Intermediates of the central carbon metabolism consist of one of the major groups of regulated metabolites throughout smut disease progression. Sugarcane meristems actively grow and accumulate higher levels of amino acids and metabolites associated with the TCA cycle as compared with stem tissues (Glassop et al., 2007). In smut-infected samples, higher levels of compounds associated with glycolysis, TCA and PPP in the meristem were detected along with increased gene expression of a soluble acid invertase. These results were also in agreement with the RNAseq and proteomic data described for whipped cane (Figure 9, Barnabas et al., 2016; Schaker et al., 2016). Up-regulation of energy-related genes is a conserved response to the demands of stresses (Less et al., 2011; Rojas et al., 2014) and may be a response to the sink imposed by whip growth in addition to sustain pathogen colonization. Increased demands of energy may consequently reduce sucrose accumulation in stems, which is one of the smut susceptible symptoms not yet investigated in the resistant genotypes colonized by the fungus. *S. scitamineum* colonization is detected even in sugarcane genotypes resistant to smut, i. e. those that do not emit a whip (Carvalho et al., 2016).

Regarding carbon storage, was detected an increased gene expression of sugarcane starch synthase at 100 DAI. We speculated that starch might be a source to feed whip in its later stages of development. RNAseq data from plants after whip development showed the up-regulation of an alpha-amylase, involved in the starch breakdown (Schaker et al., 2016), whereas starch accumulation was detected in both meristem and whip base. Whip emission probably share some components of flowering pathway (Schaker et al., 2016). Decrease in abundance of cell wall components starting at 100 DAI can be associated to an earlier sign of inflorescence initiation and shoot apical meristem enlargement, where cells cease development of secondary cell walls (Moore and Berding, 2014).

Other relevant metabolic changes in the infected samples involved accumulation of amino acids, mainly at 65 DAI. At this time point, the infected samples accumulated Pro and reduced levels of Ala, Arg, Gly, and Ser. Plants are known to increase Pro levels during stress acting as a potent osmolyte, metal chelator, antioxidant, or signaling molecule associated with the hypersensitive response (Fabro et al., 2004; Verslues and Sharma, 2010; Hayat et al., 2012; Qamar et al., 2015). Whereas, Pro accumulation may also favor fungal development by protecting hyphae against damage caused by hydrogen peroxide produced by the host (Chen and Dickman, 2005) Because there is a clear shift of sugarcane metabolism between 65 and 100 DAI, it would be interesting to test Pro as signaling for fungal sporogenesis. Increased proline levels may also be related to the reduced GABA (γ-Aminobutyric acid) levels detected infected plants 65 DAI. The GABA synthesis potentially competes for the same substrate (Verslues and Sharma, 2010).

Increase in Glu, Asp and Arg in the late stages of smut may reflect a response in the nitrogen status of infected samples. In higher plants, inorganic nitrogen is first reduced to ammonia, and then assimilated into organic form as Gln and Glu, which can be used to form Asp and Asn, and these four amino acids are used to translocate organic nitrogen from sources to sinks (Coruzzi, 2003). These results are corroborated by RNAseq data obtained before (Schaker et al., 2016). Smutted-sugarcane after whip emission showed up-regulation of genes involved in these amino acids biosynthesis, such as asparagine synthase (EC 6.3.5.4), aspartate transaminase (EC 2.6.1.1), glutamine synthetase (EC 6.3.1.2) and glutamine-dependent carbamoyl phosphate synthetase (EC 6.3.5.5), indicating increased nitrogen demands on meristem of diseased plants.

Other amino acids responsive to whip development, Tyr and Met, which were also detected in previous studies (Figure 9, Barnabas et al., 2016; Schaker et al., 2016), supported the hypothesis that increased lignin contents and ethylene imbalance are modulated by the fungus in this pathosystem. Lignin is a component of plant cell walls and was thought to be mostly produced from L-phenylalanine (Phe). Plants undergoing normal development can direct more than 30% of photosynthetically fixed carbon through the vascular system to synthesize lignin via the phenylpropanoid pathway (Maeda and Dudareva, 2012). However, in grasses nearly half of the plant's lignin is known to be synthesized through fewer steps via Tyr, using a different path of that related to Phe, leading to the formation of 4-coumarate (Barros et al., 2016). Increased levels of Tyr were detected in infected plants at 100 and 120 DAI along with the up-regulation of PTAL gene (Schaker et al., 2016) and a PTAL protein exclusively detected in whipped plants (Barnabas et al., 2016). Moreover, transcriptional and protein analysis revealed increased levels of cinnamoyl-CoA reductase, caffeic acid 3-O-methyltransferase, caffeoyl-CoA O-methyltransferase and peroxidases (Barnabas et al., 2016; Schaker et al., 2016). These data altogether indicated that Tyr accumulation might be related to phenylpropanoids pathway and lignin production, by either as a substrate for lignin formation or as a signal molecule to increase PTAL gene expression.

Met is considered as a fundamental metabolite of plant cells involved in the biosynthesis of ethylene and polyamines via SAM (S-adenosylmethionine) (Roje, 2006). This pathway is regulated by a feedback mechanism, where the levels of the first committed enzyme of Met biosynthesis, cystathionine γ-synthase (CGS), is downregulated by SAM (Chiba et al., 2003). Reduced levels of Met in samples after whip emission may be related to its conversion to SAM, which in turn negatively affects Met synthesis. The transcriptome data revealed that CGS-encoding gene is downregulated, whereas the gene for S-adenosylmethionine synthetase is up-regulated in samples after whip development (Figure 9, Schaker et al., 2016). Accordingly, the protein level of methionine synthase is reduced, and SAM increased at the same time (Barnabas et al., 2016). Furthermore, the increased expression of ACC oxidase gene indicates that SAM can be used as a precursor to ethylene biosynthesis in infected samples, a plant hormone known to be positively correlated with the activation of the phenylpropanoid pathway (Guo and Ecker, 2004).

Phenylpropanoid pathway is also the source of precursors involved in the secondary metabolism. In this study a surprising response was obtained. A compound related to maysin and its derivatives was identified at all time points analyzed by the LC-ESI-MS analysis. Maysin is recognized by its potential to confer resistance against the lepidopteran corn earworm in maize (Wiseman et al., 1984). Two metabolite precursors of its synthesis (derhamnosylmaysin and 3′-O-methylderhamnosylmaysin) were detected only in control samples at all time points, indicating that this pathway is suppressed in response to *S. scitamineum* colonization. It may be worthwhile to evaluate if this same pattern is observed in other sugarcane genotypes, and correlate it with plant resistance.

LC-ESI-MS approach also allowed the identification of a metabolite with fragmentation pattern similar to Fusarin C. This mycotoxin belongs to the class of acyl-tetramic acids, which are found in several fungi (Song et al., 2004). The fusarin gene cluster consists of nine genes (*fus1*–*fus9*) that are coexpressed under high-nitrogen and acidic pH conditions (Niehaus et al., 2013). In *S. scitamineum*, the studies related to mycotoxins are still scarce. We searched for gene clusters related to secondary metabolic pathways in the genome of *S. scitamineum* (Taniguti et al., 2015). Forty-three clusters were identified, but none of them were homologous to the *fus1-fus9* cluster. It should be further studied possible ways to produce molecules, such as the fusarin-like identified in sugarcane smutted plants (Supporting Information File S4). The detection of fungal toxins in sugarcane juices, such as aflatoxin B1 and G1 (Abdallah et al., 2016) alerts to issues concerning food safety, since sugarcane is the main source of sugar, and hosts a large diversity of potentially toxin-producing fungi.

**Figure 10:**
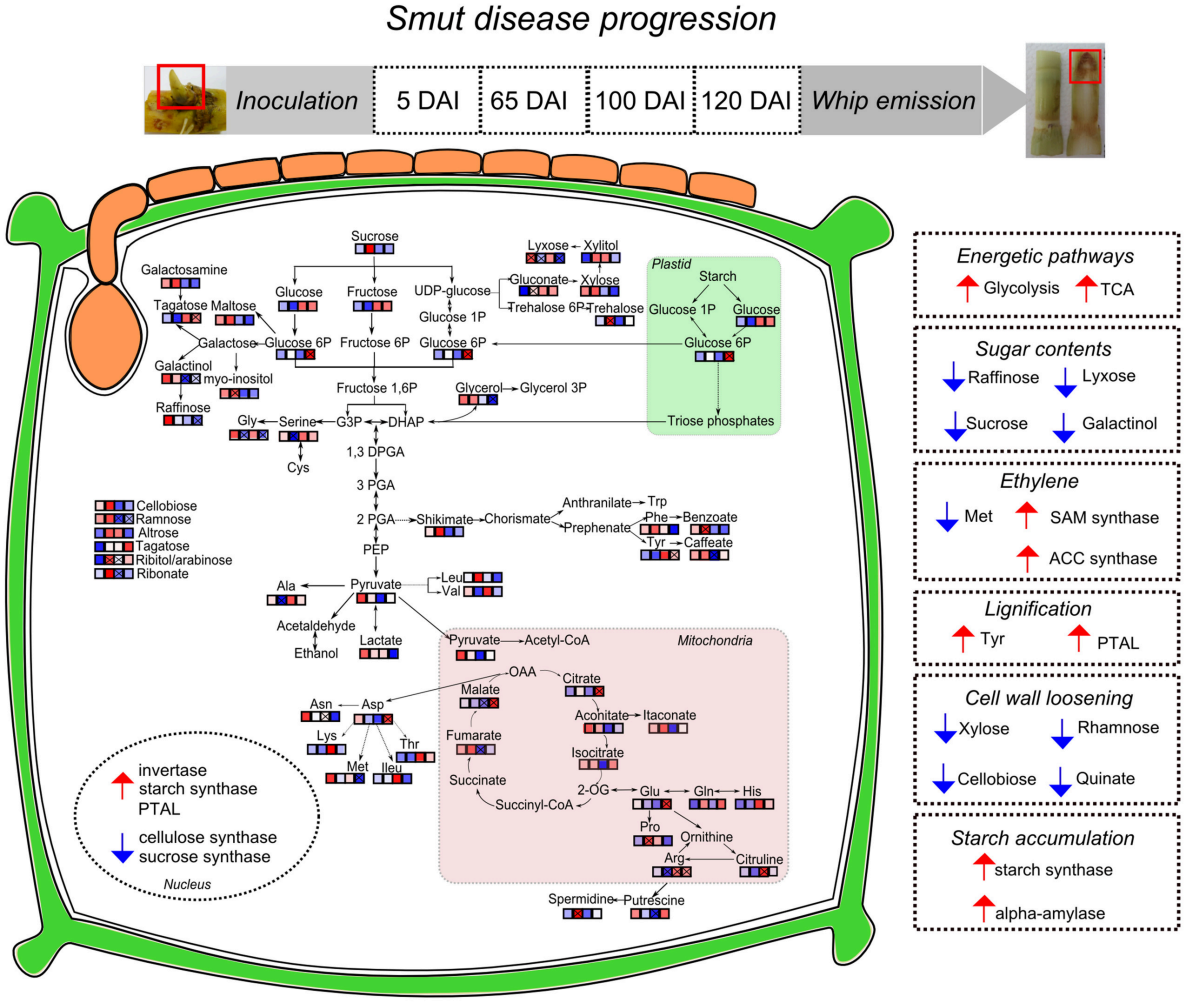
**Sugarcane responses to ***S. scitamineum*** colonization during disease progression and whip emission**. Identified metabolites in GC-MS analysis were incorporated in pathways and each square represent the sampling days after inoculation (5, 65, 100, and 120 DAI). “x” assigned represent significant regulation (*p* < 0.05), blue squares represent metabolites less accumulated and red represent more accumulated in infected samples. Nuclear compartment represents genes with differential expression in RT-qPCR analysis. Right squares in dotted lines summarize the hypotheses built on metabolomics and previous transcriptomics analysis of sugarcane-smut interaction as discussed in the main text. Smut-infected plants increase energetic demands to feed whip development, along with changes in several sugars, which may involve changes in plant signaling. Hormonal imbalance in infected plants is exemplified by ethylene biosynthesis pathway regulated in both metabolites and gene expression assays. Phenylpropanoid pathway is activated in meristems of infected plants probably through activation of a bifunctional PTAL and Tyr accumulation. Conversely, metabolites and genes related to precursors of plant cell wall are less accumulated in meristem of infected samples, indicating that cell wall loosening occurs to allow whip emission. Whip growth as well as pathogen sporulation may be fed by the starch accumulated in meristems of infected plants.

This work presented an overall view of metabolic changes within the region containing the shoot apical meristem of sugarcane smut-infected compared to non-infected plants. *S. scitamineum* initiates plant colonization in this region of sugarcane. Plants were followed throughout disease progression and metabolites examined at four time points, from early infection to whip development. The metabolomic data was integrated to previously obtained transcriptomic analysis performed in independent experiments with the same sugarcane genotype and experimental design. These complementary information were used to propose the following working model (Figure 10). Three major changes were detected during disease progression. First, there was remarkable raffinose accumulation soon after fungal infection (5DAI), which may be the signal to the subsequent sugarcane metabolic disturbances. Next, an intense shift from high to low amounts of xylose, cellobiose, rhamnose and shikimate was detected between 65 and 100 DAI. Similar response is characteristic of plants developing inflorescences and it is potentially a mechanism shared by smutted sugarcane to initiate whip development. And lastly, from 100 to 120 DAI, it was noticeable the mobilization of carbohydrates (glycolysis and TCA intermediates) and changes in amino acids contents. At this stage, demands of energy and reallocation of carbon maybe important to sustain pathogen sporulation and whip emergence (100–120 DAI). Phenylpropanoid pathways were also affected at this stage. Tyr accumulation and the activity of a bifunctional PTAL were interpreted as signs of increased lignin biosynthesis to support whip development. Differential expression of genes encoding starch synthase and alpha-amylase suggested that starch breakdown is also required for whip growth. Moreover, sugarcane secondary metabolism was affected. A number of antifungal compounds were produced supposedly to restrain pathogen growth, whereas some metabolites detected in control plants were inhibited in infected ones. Taken together, these data disclose candidate mechanisms to be further explored among genotypes with various levels of smut resistance and reveals that smut-sugarcane compatible interaction consists of a very complex mechanism involving various steps of metabolic reprogramming in response to the fungal development in plant.

## Author contributions

Conceived and designed the experiments: PS and CM. Performed the experiments: PS, LP, and TC. Analyzed the data: PS, LP, CC, and TC. Contributed reagents/materials/analysis tools: CL, CC, and CM. Wrote the paper: PS and CM. Provided expertise and editing: CC, CL, TC, and CM.

### Conflict of interest statement

The authors declare that the research was conducted in the absence of any commercial or financial relationships that could be construed as a potential conflict of interest.
