# Supplementary figures and images for: Metabolome Dynamics of Smutted Sugarcane Reveals Mechanisms Involved in Disease Progression and Whip Emission
Source: Front Plant Sci. 2017 May 31;8:882. doi: 10.3389/fpls.2017.00882 (PMC5450380; doi:10.3389/fpls.2017.00882)

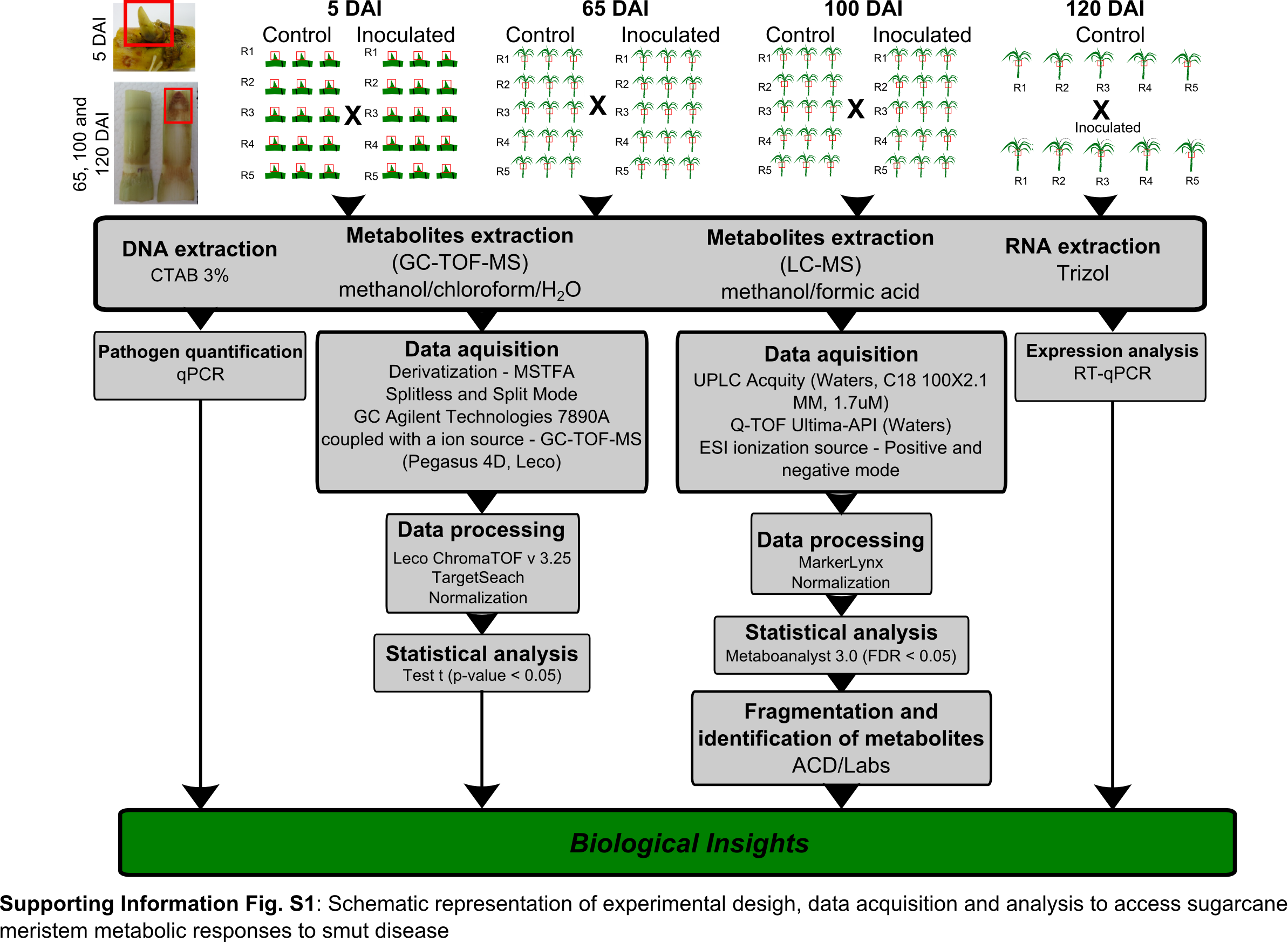

Supplement: Supplementary file 1 [file Image1.TIFF]

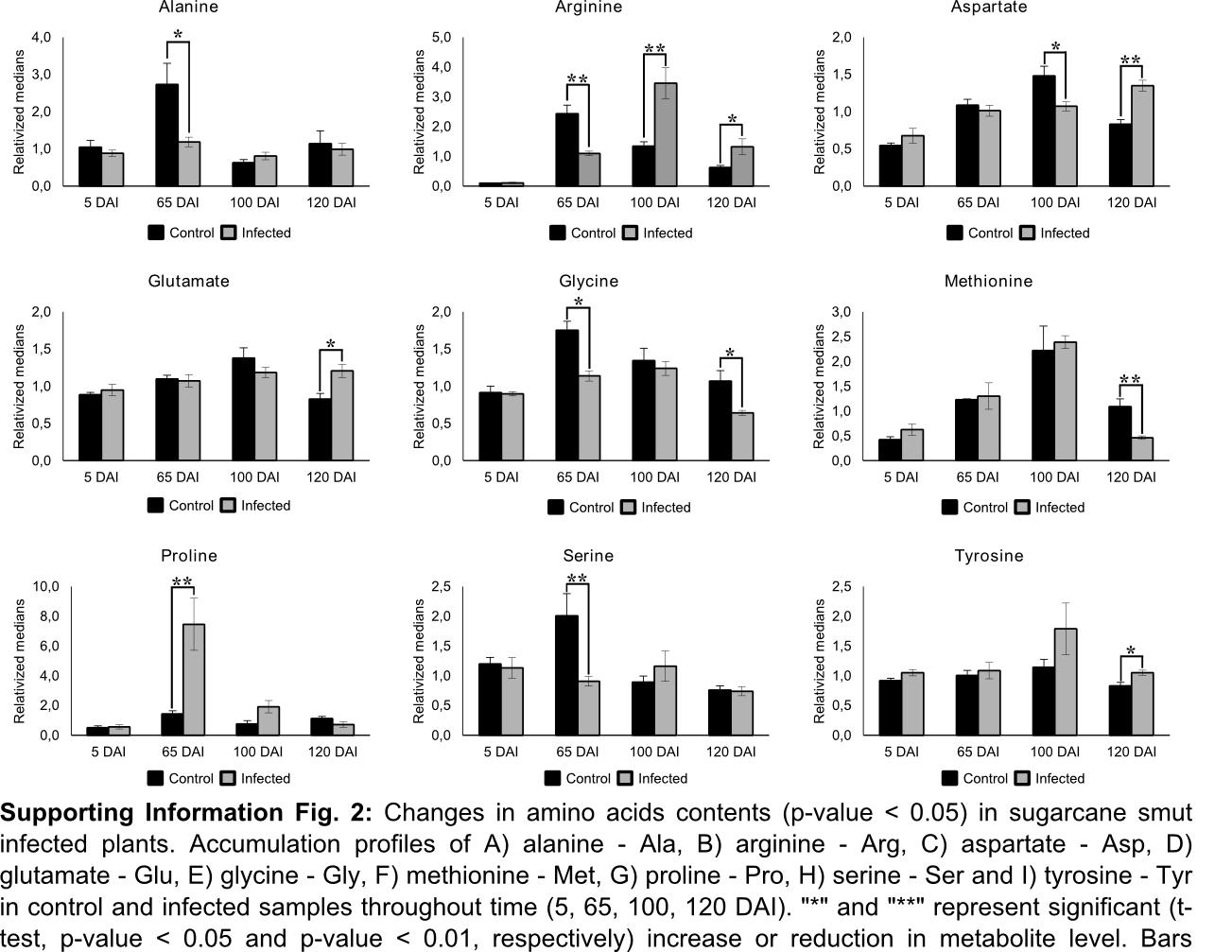

Supplement: Supplementary file 2 [file Image2.TIFF]
