# Supplementary material for: Metabolome Dynamics of Smutted Sugarcane Reveals Mechanisms Involved in Disease Progression and Whip Emission
Source: Front Plant Sci. 2017 May 31;8:882. doi: 10.3389/fpls.2017.00882 (PMC5450380; doi:10.3389/fpls.2017.00882)
Supplement: Supplementary file 3 [file Presentation1.PDF]

Supporting Information File S1. Sequences used to build the phylogenetic tree of PTAL and PAL in plants and fungi, and TAL, tyrosine ammonia-mutase (TAM) and histidine ammonia-lyase (HAL) in bacteria. Sequences were retrieved from Uniprot, Sugarcane Unigenes (Cardoso-Silva et al., 2014) and Sugarcane SP80-3280 protein sequences (http://bce.bioetanol.cnpem.br/ctbeblast/).

```
>lcl|SP803280_c117540_g1_i3_m.153092_Sugarcane_SP80-3280
FRSRQLLRLFSSSSPPAAPPPPPPSSSPPATARPQPQHSIMAGNGAIVESDPLNWGAAAAELAGSHLDEVKRMVAQARQPVVKIEGSTLRVGQ
VAAVAAAKDASGVAVELDEEARPRVKASSEWILDCIAHGGDIYGVTTGFGGTSHRRTKDGPALQVELLRHLNAGIFGTGSDGHTLPSEVVRA
AMLVRINTLLQGYSGIRFEILEAITKLLNTGVSPCLPLRGTITASGDLVPLSYIAGLITGRPNAQATTIDGRKVDAAEAFKIAGIEHGFFEL
QPKEGLAMVNGTAVGSGLASTVLFEANILAILAEVLSAVFCEVMNGKPEYTDHLTHKLKHHPGSIEAAAIMEHILDGSAFMKHAKKVNELDP
LLKPKQDRYALRTSPQWLGPQIEVIRAATKSIEREINSVNDNPLIDVSRGKALHGGNFQGTPIGVSMDNTRLALAAIGKLMFAQFSELVNDY
YNNGLPSNLSGGRNPSLDYGFKGAEIAMASYCSELQFLGNPVTNHVQSAEQHNQDVNSLGLISSRKTAEAVDILKLMTSTFLIALCQAIDLR
HIEENVKAAVKNCVTQVAKKSLSLNARGGLHNARFCEKDLQTAIDREAVFAYADDPCSPNYPLMQKLRAVLIEHALANGDAERVVETSIFAK
VAEFEQQVRAALPKEVEAARAAVESGNPLVPNRIKECRSYPLYRFVREEVGTEYLTGEKTRSPGEELNKVLVAINQRKHIDPLLECLKEWNG
EPLPLC*

>lcl|SP803280_c117540_g1_i1_m.153088_Sugarcane_SP80-3280
FRSRQLLRLFSSSSPPAAPPPPPPSSSPPATARPQPQHSIMAGNGAIVESDPLNWGAAAAELAGSHLDEVKRMVAQARQPVVKIEGSTLRVGQ
VAAVAAAKDASGVAVELDEEARPRVKASSEWILDCIAHGGDIYGVTTGFGGTSHRRTKDGPALQVELLRHLNAGIFGTGSDGHTLPSEVVRA
AMLVRINTLLQGYSGIRFEILEAITKLLNTGVSPCLPLRGTITASGDLVPLSYIAGLITGRPNAQATTIDGRKVDAAEAFKIAGIEHGFFEL
QPKEGLAMVNGTAVGSGLASTVLFEANILAILAEVLSAVFCEVMNGKPEYTDHLTHKLKHHPGSIEAAAIMEHILDGSAFMKHAKKVNELDP
LLKPKQDRYALRTSPQWLGPQIEVIRAATKSIEREINSVNDNPLIDVSRGKALHGGNFQGTPIGVSMDNTRLALAAIGKLMFAQFSELVNDY
YNNGLPSNLSGGRNPSLDYGFKGAEIAMASYCSELQFLGNPVTNHVQSAEQHNQDVNSLGLISSRKTAEAVDILKLMTSTFLIALCQAIDLR
HIEENVKAAVKNCVTQVAKKSLSLNARGGLHNARFCEKDLQTAIDREAVFAYADDPCSPNYPLMQKLRAVLIEHALANGDAERVVETSIFAK
VAEFEQQVRAALPKEVEAARAAVESGNPLVPNRIKECRSYPLYRFVREEVGTEYLTGEKTRSPGEELNKVLVAINQRKHIDPLLECLKEWNG
EPLPLC*


>lcl|scf7180000364451_g3810_Sugarcane_SP80-3280
MASNTAILESDPLNWGKAAAELTGSHLDEVKRMVAQFRDPVVKIEGSTLRVGQVAAVAAAKDASGVAVELDEEARPRVKASSEWILDCIAHG
GDIYGVTTGFGGTSHRRTKDGPALQVELLRHLNAGIFGNGSDGHTLPSEVSRAAMLVRINTLLQGYSGIRFEILEAITKLLNTGVSPCLPLR
GTITASGDLVPLSYIAGLITGRPNAQAVTVDGRKVDAAEAFKVAGIEGGFFKLNPKEGLAIVNGTSVGSALAAMVCFDANVLAVLSSVLSAV
FCEVMNGKPEYTDHLTHKLKHHPGSIESAAIMEHILDGSSFMKHAKEVNAMDPLLKPKQDRYALRTSPQWLGPQIEVIRAATKSIEREVNSV
NDNPVIDVHRGKALHGGNFQGTPIGVSMDNARLAIANIGKLMFAQFSELVNEFYNNGLTSNLAGSRNPSLDYGFKGTEIAMASYCSELQYLA
NPITNHVQSAEQHNQDVNSLGLVSARKTAEAVDILKLMSSTYMVALCQAVDLRHLEENLKSAVKNSVMAVARKVLTTSLDGDLHSARFSEKA
LLTAIDREAVYGYYDDPCSANSPLMKKIRAVLVDHALANGEAEKDASASVFSKINRFEETLREALPREMEAARVAFETGTAPIANRIKESRS
YPLYRFIRQDLGAVYLTGEKLKSPGEECNKVFLALSEGKLIDPMLECLKEWDGKPLPIC

>lcl|scf7180000358933_g4603_Sugarcane_SP80-3280
MASNTAILESDPLNWGKAAAELTGSHLDEVKRMVAQFRDPIVKIEGSTLRVGQVAAVAAAKDASGVAVELDEEARPRVKASSEWILDCIAHG
GDIYGVTTGFGGTSHRRTKDGPALQVELLRHLNAGIFGNGSDGHTLPSEVSRAAMLVRINTLLQGYSGIRFEILETITKLLNTGVSPCLPLR
GTITASGDLVPLSYIAGLITGRPNAQAVTVDGRKVDAAEAFKVAGIEGGFFKLNPKEGLAIVNGTSVGSALAAVVCFDANVLAVLSSVLSAV
FCEVMNGKPEYTDHLTHKLKHHPGSIESAAIMEHILDGSSFMKHAKEVNAMDPLLKPKQDRYALRTSPQWLGPQIEVIRAATKSIEREVNSV
NDNPVIDVHRGKALHGGNFQGTPIGVSMDNARLAIANIGKLMFAQFSELVNEFYNNGLTSNLAGSRNPSLDYGFKGTEIAMASYCSELQYLA
NPITNHVQSAEQHNQDVNSLGLVSARKTAEAVDILKLMSSTYMVALCQAVDLRHLEENLKSAVKNSVMAVARKVLTTSLDGDLHSARFSEKA
LLTAIDREAVYGYYDDPCSANSPLMKKIRAVLVDHALANGEAEKDASASVFSKINRFEETLREVLPREMEAARVAFETGTAPIANRIKESRS
YPLYRFIRQDLGAVYLTGEKLKSPGEECNKVFLALSEGKLIDPMLECLKEWDGKPLPIC


>lcl|scf7180000385419_g1312_Sugarcane_SP80-3280
MATPRADPLNWGKAAEELMGSHLDEVKRMVAEYRQPLVKIEGASLRIAQVAAVAAGAGEARVELDEYARGRVKASSDWVMNSMMNGTDSYGV
TTGFGATSHRRTKEGGALQRELIRFLNAGAFGTGTDGHVLPAEATRAAMLVRINTLLQGYSGIRFEILEAIVKLLNANVTPCLPLRGTVTAS
GDLVPLSYIAGLVTGRENSVAVAPDGSKVNAAEAFKIAGIQGGFFELQPKEGLAMVNGTAVGSGLASTVLFEANILAVLAEVLSAVFCEVMN
GKPEYTDHLTHKLKHHPGQIEAAAIMEHILEGSSYMKLAKKLGELDPLMKPKQDRYALRTSPQWLGPQIEVIRAATKSIEREINSVNDNPLI
DVARSKALHGGNFQGTPIGVSMDNTRLAIAAIGKLMFAQFSELVNDYYNNGLPSNLSGGRNPSLDYGFKGAEIAMASYCSELQFLGNPVTNH
VQSAEQHNQDVNSLGLISSRKTAEAIEILKLMSSTFLIALCQAVDLRHIEENVKSAVKSCVMMVAKKTLSTNSTGGLHVARFCEKDLLQEIE
REAVFAYADDPCSANYPLMNKLRNVLVERALANGAAEFDAETSVFAKVAQFEEELRAALPKAVEAARTAVENGTAAIPNRITECRSYPLYRF
VREELGAVYLTGEKTRSPGEELNKVLVAINQGKHIDPLLECLKEWNGEPLPIC

>lcl|scf7180000352418_g3381_Sugarcane_SP80-3280
MATPRADPLNWGKAAEELMGSHLDEVKRMVAEYRQPLVKIEGASLRIAQVAAVAAGAGEARVELDESARGRVKASSDWVMNSMMNGTDSYGV
TTGFGATSHRRTKEGGALQRELIRFLNAGAFGTGTDGHVLPAEATRAAMLVRINTLLQGYSGIRFEILEAIVKLLNANVTPCLPLRGTVTAS
GDLVPLSYIAGLVTGRENSVAVAPDGSKVNAAEAFKIAGIQGGFFELQPKEGLAMVNGTAVGSGLASTVLFEANILAVLAEVLSAVFCEVMN
GKPEYTDHLTHKLKHHPGQIEAAAIMEHILEGSSYMKLAKKLGELDPLMKPKQDRYALRTSPQWLGPQIEVIRAATKSIEREINSVNDNPLI
DVARSRALHGGNFQGTPIGVSMDNTRLAIAAIGKLMFAQFSELVNDYYNNGLPSNLSGGRNPSLDYGFKGAEIAMASYCSELQFLGNPVTNH
VQSAEQHNQDVNSLGLISSRKTAEAIEILKLMSSTFLIALCQAVDLRHIEENVKSAVKSCVMTVAKKTLSTNSTGGLHVARFCEKDLLQEIE
```

REAVFAYADDPCSANYPLMKKLRNVLVERALANGAAQFDAETSVFAKVAQFEEELRAALPKAVEAARAAVENGTAAIPNRITECRSYPLYRF
VRQELGAVYLTGEKTRSPGEELNKVLVAINQGKHIDPLLECLKEWNGEPLPIC

>lcl|scf7180000345725_g1030_Sugarcane_SP80-3280
MECENGRVAATNGDSLCMATPRADPLNWGKAAEELMGSHLDEVKRMVAEYRQPLVKIEGASLRIAQVAAVAAGAGGARVELDESARGRVKAS
SDWVMNSMMNGTDSYGVTTGFGATSHRRTKEGGALQRELIRFLNAGAFGTGTDGHVLPAEATRAAMLVRINTLLQGYSGIRFEILEAIVKLL
NANVTPCLPLRGTVTASGDLVPLSYIAGLVTGRENSVAVAPDGSKVNAAEAFKIAGIQGGFFELQPKEGLAMVNGTAVGSGLASTVLFEANI
LAILAEVLSAVFCEVMNGKPEYTDHLTHKLKHHPGQIEAAAIMEHILEGSSYMKLAKKLGELDPLMKPKQDRYALRTSPQWLGPQIEVIRAA
TKSIEREINSVNDNPLIDVARSKALHGGNFQGTPIGVSMDNTRLAIAAIGKLMFAQFSELVNDYYNNGLPSNLSGGRNPSLDYGFKGAEIAM
ASYCSELQFLGNPVTNHVQSAEQHNQDVNSLGLISSRKTAEAIETLKLMSSTFLIALCQAVDLRHIEENVKSAVKSCVMTVAKKTLSTNSTG
GLHVARFCEKDLLQEIDREAVFAYADDPCSANYPLMKKLRNVLVERALANGAAEFDAETSVFAKVAQFEEELRAALPKAVEAARAAVENGTA
VIPNRITECRSYPLYRFVREELGAVYLTGEKTRSPGEELNKVLLAINQGKHIDPLLECLKEWNGEPLPIC

>lcl|deg7180000227039_g6315_Sugarcane_SP80-3280
MECENGRVAATNGDSLCMATPRADPLNWGKAAEELMGSHLDEVKRMVAEYRQPLVKIEGASLRIAQVAAVAAGAGEARVELDESARGRVKAS
SDWVMNSMTNGTDSYGVTTGFGATSHRRTKEGGALQRELIRFLNAGAFGTGTDGHVLPAEATRAAMLVRINTLLQGYSGIRFEILEAIVKLL
NANVTPCLPLRGTVTASGDLVPLSYIAGLVTGRENSVAVAPDGSKVNAAQAFKIAGIQGGFFELQPKEGLAMVNGTAVGSGLASTVLFEANI
LAILAEVLSAVFCEVMNGKPEYTDHLTHKLKHHPGQIEAADIMEHILEGSSYMKLAKKLGELDPLMKPKQDRYALRTSPQWLGPQIEVIRAA
TKSIEREINSVNDNPLIDVARSKALHGGNFQGTPIGVSMDNTRLAIAAIGKLMFAQFSELVNDYYNNGLPSNLSGGRNPSLDYGFKGAEIAM
ASYCSELQFLGNPVTNHVQSAEQHNQDVNSLGLISSRKTAEAIEILKLMSSTFLIALCQAVDLRHIEENVKSAVKGCVMTVAKKTLSTNSTG
GLHVARFCEKDLLQEIEREAVFAYADDPCSANYPLMKKLRNVLVERALANGAAEFDAETSVFAKVAQFEEELRAALPKAVEAARAAVENGTA
AIPNRITECRSYPLYRFVREELGAVYLTGEKTRSPGEELNKVLLAINQGKHIDPLLECLKEWNGEPLPIC

>lcl|scf7180000345474_g578_Sugarcane_SP80-3280
MECENGRVAATNGDSLCMATPRTDPLNWGKAAEELMGSHLDEVKRMVAEYRQPLVKIEGASLRIAQVAAVAAGAGEARVELDESARGRVKAS
SDWVMNSMMNGTDSYGVTTGFGATSHRRTKEGGALQRELIRFLNAGAFGTGTDGHVLPAEATRAAMLVRINTLLQGYSGIRFEILEAIVKLL
NANVTPCLPLRGTVTASGDLVPLSYIAGLVTGRENSVAVAPDGSKVNAAEAFKIAGIQGGFFELQPKEGLAMVNGTAVGSGLASTVLFEANI
LAILAEVLSAVFCEVMNGKPEYTDHLTHKLKHHPGQIEAAAIMEHILEGSSYMKLAKKLGELDPLMKPKQDRYALRTSPQWLGPQIEVIRAA
TKSIEREINSVNDNPLIDVARSKALHGGNFQGTPIGVSMDNTRLAIAAIGKLMFAQFSELVNDYYNNGLPSNLSGGRNPSLDYGFKGAEIAM
ASYCSELQFLGNPVTNHVQSAEQHNQDVNSLGLISSRKTAEAIEILKLMSSTFLIALCQAVDLRHIEENVKSTVKSCVMTVAKKTLSTNSTG
GLHVARFCEKDLLQEIEREAVFAYADDPCSANYPLMKKLRNVLVERALANGAAEFDAETSVFVKVAQFEEELRAALPKAVEAARAAVENGTA
AIPNRITECRSYPLYRFVREELGAVYLTGEKTRSHGEELNKVLLAINQGKHIDPLLECLKEWNGEPLPIC

>lcl|scf7180000378773_g3317_Sugarcane_SP80-3280
MERVVKDYVVAKYVQVSNLGELMGSHLDEVKRMVAEYRQPLVKIEGASLRIAQVAAVAAGAGEARVELDESARGRVKASSDWVMNSMMNGTD
SYGVTTGFGATSHRRTKEGGALQRELIRFLNAGAFGTGTDGHVLPAEATRAAMLVRINTLLQGYSGIRFEILEAIVKLLNANVTPCLPLRGT
VTASGDLVPLSYIAGLVTGRENSVAVAPDGSKVNAAEAFKIAGIQGGFFELQPKEGLAMVNGTAVGSGLASTVLFEANILAILAEVLSAVFC
EVMNGKPEYTDHLTHKLKHHPGQIEAAAIMEHILEGSSYMKLAKKLGELDPLMKPKQDRYALRTSPQWLGPQIEVIRAATKSIEREINSVND
NPLIDVARSKALHGGNFQGTPIGVSMDNTRLAIAAIGKLMFAQFSELVNDYYNNGLPSNLSGGRNPSLDYGFKGAEIAMASYCSELQFLGNP
VTNHVQSAEQHNQDVNSLGLISSRKTAEAIEILKLMSSTFLIALCQAVDLRHIEENVKSAVKSCVMTVAKKTLSTDSTGGLHVARFCEKDLL
QEIEREAVFAYADDPCSANYPLMKKLRNVLVERALANGAAEFNAETSVFAKVAQFEEELRAALPKAVEAARTAVENGTAAIPNRITECRSYP
LYRFVREEVGAVYLTGEKTRSPGEELNKVLLAINQGKHIDPLLECLKEWNGEPLPIC

>lcl|scf7180000389185_g6760_Sugarcane_SP80-3280
MAGNGAIVESDPLNWGAAAAELAGSHLDEVKRMVAQARQPVVKIEGSTLRVGQVAAVAAAKDASGVAVELDEEARPRVKASSEWILDCIAHG
GDIYGVTTGFGGTSHRRTKDGPALQVELLRHLNAGIFGTGSDGHTLPSEVVRAAMLVRINTLLQGYSGIRFEILEAITKLLNTGVSPCLPLR
GTITASGDLVPLSYIAGLITGRPNAQATTIDGRKVDAAEAFKIAGIEGGFFKLNPKEGLAIVNGTSVGSALAATVMYDANVLAILSEVLSAV
FCEVMNGKPEYTDHLTHKLKHHPGSIEAAAIMEHILDGSAFMKHAKKVNELDPLLKPKQDRYALRTSPQWLGPQIEVIRAATKSIEREVNSV
NDNPVIDVHRGKALHGGNFQGTPIGVSMDNARLAIANIGKLMFAQFSELVNEFYNNGLTSNLAGSRNPSLDYGFKGTEIAMASYCSELQYLG
NPITNHVQSAEQHNQDVNSLGLVSARKTAEAIDILKLMSSTYIVALCQAIDLRHLEENIKTSVKNTVTQVAKKVLTMNPSGDLSSARFSEKE
LITAIDREGVFTYAEDPASGC

>lcl|scf7180000335936_g368_Sugarcane_SP80-3280
MTLLFSTFTKLATVRLHLVLSDCQAVFSNEFIYRCCQTDPCVLPCRHLNAGIFGTGSDGHTLPSEVVRAAMLVRINTLLQGYSGIRFEILEA
ITKLLNTGVSPCLPLRGTITASGDLVPLSYIAGLITGRPNAQATTIDGRKVDAAEAFKIAGIEGGFFKLNPKEGLAIVNGTSVGSALAATVM
YDANVLAVLSEVLSAVFCEVMNGKPEYTDHLTHKLKHHPGSIEAAAIMEHILDGSAFMKHAKKVNELDPLLKPKQDRYALRTSPQWLGPQIE
VIRAATKSIEREVNSVNDNPVIDVHRGKALHGGNFQGTPIGVSMDNARLAIANIGKLMFAQFSELVNEFYNNGLTSNLAGSRNPSLDYGFKG
TEIAMASYCSELQYLGNPITNHVQSAEQHNQDVNSLGLVSARKTAEAIDILKLMSSTYIVALCQAIDLRHIEENIKTSVKNTVTQVAKKVLT
MNPSGDLSSARFSEKELITAIDREGVFTYAEDPASGSLPLMQKLRAVLVDHALSSGDAEREPSVFSKITKFEEELRAVLPREVEAARVAVAE
GTAPVANRISDSRSFPLYRFVREELGCVFLTGEKLKSPGEECTKVFNGINQGKLVDPMLECLKEWDGKPLPINVVN

>lcl|deg7180000178341_g5940_Sugarcane_SP80-3280
MTLLFSTFTKLATVRLHLVLSDCQAVFSNEFIYRCCQTDPCALPCRHLNAGIFGTGSDGHTLPSEVVRAAMLVRINTLLQGYSGIRFEILES
ITKLLNTGVSPCLPLRGTITASGDLVPLSYIAGLITGRPNAQATTVDGRKVDAAEAFKIAGIEGGFFKLNPKEGLAIVNGTSVGSALAATVM
YDANVLTVLSEVLSAVFCEVMNGKPEYTDHLTHKLKHHPGSIEAAAIMEHILDGSAFMKHAKKVNELDPLLKPKQDRYALRTSPQWLGPQIE
VIRAATKSIEREVNSVNDNPVIDVHRGKALHGGNFQGTPIGVSMDNARLAIANIGKLMFAQFSELVNEFYNNGLTSNLAGSRNPSLDYGFKG
TEIAMASYCSELQYLGNPITNHVQSAEQHNQDVNSLGLVSARKTAEAIDILKLMSSTYIVALCQAIDLRHLEENIKTSVKNTVTQVAKKVLT
MNPSGDLSSARFSEKELITAIDREGVFTYAEDPASGSLPLMQKLRAVLVDHALSSGDAEREPSVFSKITKFEEELRAVLPREVEAARVAVAE
GTAPVANRIADSRSFPLYRFVREELGCVFLTGEKLKSPGEECTKVFNGINQGKLVDPMLECLKEWDGKPLPINVVN

>lcl|deg7180000273052_g4864_Sugarcane_SP80-3280

DSYGVTTGFGATSHRRTKEGGALQRELIRFLNAGAFGTGDDGHVLPAAATRAAMLVRINTLLQGYSGIRFEILETIAALLNANVTPCLPLRG
TITASGDLVPLSYIAGLVTGRPNSTAVAPDGRKVDAAEAFKIAGIQHGFFELQPKEGLAMVNGTAVGSGLASIVLFEANVLAVLAEVMSAVF
CEVMNGKPEYTDHLTHKLKHHPGQIESAAIMEHILDGSSYMMLAKKLGELDPLMKPKQDRYALRTSPQWLGPQIEVIRAATKSIEREINSVN
DNPLIDVSRGKALHGGNFQGTPIGVSMDNTRLAIAAIGKLMFAQFSELVNDFYNNGLPSNLSGGRNPSLDYGFKGAEIAMASYCSELQFLAN
PVTNHVQSAEQHNQDVNSLGLISSRKTAEAVDVLKLMSSTFLIALCQAVDLRHLEENLKSAVKSCVMTVAKKTLSTSATGTLHNARFCEKDL
LTAIDREAVFAYADDPCSANYPLMQKMRSVLVEHALANGEAERNPDTSVFAKLATFEEELRAALPREVEAARAAVENGTAAIPNRIAECRSY
PLYRFVREELGTEYLTGEKTRSPGEEVNKVFVAMNLGKHIDAVLECLKEWNGEPL

>lcl|scf7180000389185_g6766_Sugarcane_SP80-3280
MLVRINTLLQGYSGIRFEILEAIAKLLNANVTPCLPLRGTITASGDLVPLSYIAGLITGRQNSVAVAPDGRKVDAAEAFKIAGIEHGFFELQ
PKEGLAMVNGTAVGSGLASTVLFEANVLATMAEVISAVFCEVMTGKPEFTDHLTHKLKHHPGQIEAAAIMEHILEGSSYMKLAKKLGELDPL
MKPKQDRYALRTSPQWLGPQIEVIRFATKSIEREINSVNDNPLIDVSRGKALHGGNFQGTPIGVSMDNTRLALAAIGKLMFAQFSELVNDYY
NNGLPSNLSGGRNPSLDYGFKGAEIAMASYCSELQFLGNPVTNHVQSAEQHNQDVNSLGLISSRKTAEAVDILKLMTSTFLIALCQAIDLRH
IEENVKAAVKNCVTQVAKKSLSLNARGGLHNARFCEKDLQTAIDREAVFAYADDPCSPNYPLMQKLRAVLIEHALANGDAERVVETSIFAKV
AEFEQQVRAALPKEMDAARAAVESGNPLVPNRIKECRSYPLYRFVREEVGTEYLTGEKTRSPGEELNKVLVAINQRKHIDPLLECLKEWNGE
PLPLC

>comp192201_c0_seq1_Sugarcane_unigenes
EENLKSAVKNSVMAVARKVLTTSLDGDLHSARFSEKALLTAIDREAVYGYYDDPCSANSPLMKKIRAVLVDHALANGEAEKDASASVFSKIN
RFEETLREVLPREMEAARVAFETGTAPIANRIKESRSYPLYRFIRQDLGAVYLTGEKLKSPGEECNKVFLALSEGKLIDPMLECLKEWDGKP
LPIC

>SHCRBa_019_F13_F_30_1_bac_Cana
MASNTAILESDPLNWGKAAAELTGSHLDEVKRMVAQFRDPVVKIEGSTLRVGQVAAVAAAKDASGVAVELDEEARPRVKASSEWILDCIAHG
GDIYGVTTGFGGTSHRRTKDGPALQVELLRHLNAGIFGNGSDGHTLPSEVSRAAMLVRINTLLQGYSGIRFEILEAITKLLNTGVSPCLPLR
GTITASGDLVPLSYIAGLITGRPNAQAVTVDGRKVDAAEAFKVAGIEGGFFKLNPKEGLAIVNGTSVGSALAAMVCFDANVLAVLSSVLSAV
FCEVMNGKPEYTDHLTHKLKHHPGSIESAAIMEHILDGSSFMKHAKEVNAMDPLLKPKQDRYALRTSPQWLGPQIEVIRAATKSIEREVNSV
NDNPVIDVHRGKALHGGNFQGTPIGVSMDNARLAIANIGKLMFAQFSELVNEFYNNGLTSNLAGSRNPSLDYGFKGTEIAMASYCSELQYLA
NPITNHVQSAEQHNQDVNSLGLVSARKTAEAVDILKLMSSTYMVALCQAVDLRHLEENLKSAVKNSVMAVARKVLTTSLDGDLHSARFSEKA
LLTAIDREAVYGYYDDPCSANSPLMKKIRAVLVDHALANGEAEKDASASVFSKINRFEETLREVLPREMEAARVAFETGTAPIANRIKESRS
YPLYRFIRQDLGAVYLTGEKLKSPGEECNKVFLALSEGKLIDPMLECLKEWDGKPLPIC

>comp94629_c0_seq1_Sugarcane_unigenes_Cardoso-Silva et al 2014
MAGNGAIVESDPLNWGAAAAELAGSHLDEVKRMVAQARQPVVKIEGSTLRVGQVAAVAAAKDASGVAVELDEEARPRVKASSEWILDCIAHG
GDIYGVTTGF

>750133730_Saccharum_officinarum_Barnabas et al 2016
XXXKDGPALQVELLRHLNAGIFGTGSDGHTLPSEVVRAAMLVRINTLLQGYSGIRFEILEAITKLLNTGVSPCLPLRGTITASGDLVPLSYI
AGLITGRPNAQATTIDGRKVDAAEAFKIAGIEGGFFKLNPKEGLAIVNGTSVGSALAATVMYDANVLAVLSEVLSAVFCEVMNGKPEYTDHL
THKLKHHPGSIEAAAIMEHILDGSAFMKHAKKVNELDPLLKPKQDRYALRTSPQWLGPQIEVIRAATKSIEREVNSVNDNPVIDVHRGKALH
GGNFQGTPIGVSMDNARLAIANIGKLMFAQFSELVNEFX

>comp202731_c0_seq1_Sugarcane_unigenes_Cardoso-Silva et al 2014
MECENGHVAAASGNGVCLATPRAADPLNWGKAAEDLTGSHLEAVKRMVEEYRRPLVKIEGGSLTVAQVAAVAAAGEARVELDESARGRVKAS
SDWVMSSMMNGTDSYGVTTGFGATSHRRTKEGGALQRELIRFLNAGAFGTGADGHVLPAEATRAAMLVRINTLLQGYSGIRFEILEAIAKLL
NANVTPCLPLRGTITASGDLVPLSYIAGLITGRQNSVAVAPDGRKVDAAEAFKIAGIEHGFFELQPKEGLAMVNGTAVGSGLASTVLFEANV
LATMAEVISAVFCEVMTGKPEFTDHLTHKLKHHPGQIEAAAIMEHILEGSSYMKLAKKLGELDPLMKPKQDRYALRTSPQWLGPQIEVIRFA
TKSIEREINSVNDNPLIDVSRGKALHGGNFQGTPIGVSMDNTRLALAAIGKLMFAQFSELVNDYYNNGLPSNLSGGRNPSLDYGFKGAEIAM
ASYCSELQFLGNPVTNHVQSAEQHNQDVNSLGLISSRKTAEAVDILKLMTSTFLIALCQAIDLRHIEENVKAAVKNCVTQVAKKSLSLNARG
GLHNARFCEKDLQTAIDREAVFAYADDPCSPNYPLMQKLRAVLIEHALANGDAERVVETSIFAKVAEFEQQVRAALPKEVEAARAAVESGNP
LVPNRIKECRSYPLYRFVREEVGTEYLTGEKTRSPGEELNKVLVAINQRKHIDPLLECLKEWNGEPLPLC

>gg_14643_Sugarcane_unigenes_Schaker et al 2016
MASNTAILESDPLNWGKAAAELTGSHLDEVKRMVAQFRDPVVKIEGSTLRVGQVAAVAAAKDASGVAVELDEEARPRVKASSEWILDCIAHG
GDIYGVTTGFGGTSHRRTKDGPALQVELLRHLNAGIFGTGSDGHTLPSEVVRAAMLVRINTLLQGYSGIRFEILEAITKLLNTGVSPCLPLR
GTITASGDLVPLSYIAGLITGRPNAQATTIDGRKVDAAEAFKIAGIEGGFFKLNPKEGLAIVNGTSVGSALAATVMYDANVLTVLSEVLSAV
FCEVMNGKPEYTDHLTHKLKHHPGSIESAAIMEHILDGSSFMKHAKEVNAMDPLLKPKQDRYALRTSPQWLGPQIEVIRAATKSIEREVNSV
NDNPVIDVHRGKALHGGNFQGTPIGVSMDNARLAIANIGKLMFAQFSELVNEFYNNGLTSNLAGSRNPSLDYGFKGTEIAMASYCSELQYLA
NPITNHVQSAEQHNQDVNSLGLVSARKTAEAIDILKLMSSTYIVALCQAIDLRHLEENIKTSVKNTVTQVAKKVLTMNPSGDLSSARFSEKE
LITAIDREGVFTYAEDPASGSLPLMQKLRSVLVDHALSSGDAEREPSVFSKITKFEEELRAVLPREVEAARVAVAEGTAP

>Q96V77_Ustilago_maydis
MAPTADVLPPVEASTRPGLLVQPSDTKLRKASSFRTEQVVIDGYNLKIQGLVASARYGHVPVLDPSAETRKRIDDSVQSLIAKLDRGESIYG
INTGFGGSADSRTANTRALQLALLQMQQCGVLPVPSTFPTGEPSSAPFALPLTDTESSLIMPEAWVRGAIVVRLSSLMRGHSGVRWEVLDKM
QKLFLQNNVTPVVPVRSSISASGDLSPLSYVAGALAGQRGIYCFVTDGRGQRVKVTADEACRMHKITPVQYEPKEALGLLNGTAFSASVAGL
ATYEAENLASLTQLTTAMAVEALKGTDASFAPFIHEIARPHPGQIKSAKFIRALLSGSRLAEHLENEKHVLFSEDNGTLRQDRYTLRTASQW
VGPGLEDIENAKRSVDIEINSTTDNPMIDPYDGDGRIHHGGNFQAMAMTNAVEKIRLALCAMGKMTFQQMTELVNPAMNRGLPANLASTPDL
SLNFHAKGIDIALASVTSELMFLGNPVSTHVQSAEMANQAINSLALISGRQTLQAIECLSMIQAWSLYLLCQALDIRALQYKVAEQLPTLIL
ASLHSHFGEWMDETKQQEIAAQVLKSMSKRLDETSSKDLRDRLVETYQDASSVLVRYFSELPSGGGADPLRNIVKWRATGVADTEKIYRQVT
IEFLDNPYACHASHLLGKTKRAYEFVRKTLGVPMHGKENLNEFKGEFEQWNTTGGYVSVIYASIRDGELYNMLSELERDL

>A0A0F7SDN6_Sporisorium_scitamineum
MAPTADVLPAVETCARPGLLVQLCDTKIRKASSFRTEQVVIDGNNLGIQGLVASARYGHVPVLDASSATRKRIDDSVQSLIAKLDRGESIYG
INTGFGGSADSRTANTRALQLALLQMQQCGVLPVPSTFPTGEPSSAPFALPLTDTESSLIMPEAWVRGAIVVRLSSLMRGHSGVRWEVLDKM
QKLFLQNNVTPVVPVRSSISASGDLSPLSYVAGALAGQRGIYCFVTGKNGQRVKVTADEACRMHNITPVQYEPKEALGLLNGTAFSASVAGL
ATYEAEKLAALTQLTTAMAVEALKGTDASFAPFIHEIARPHPGQIKSAKFIRALLSGSKLAEHLENEKHVLFSEDNGTLRQDRYTLRTASQW
VGPGLEDIENAKRSVDIEINSTTDNPMIDPYDGDGRIHHGGNFQAMAMTNAVEKIRLALCAMGKMTFQQMTELVNPAMNRGLPANLASTPDL
SLNFHAKGIDIALASVTSELMFLGNPVSTHVQSAEMANQAINSLALISGRQTLQAVECLSMIQAWSLYLLCQALDIRALQHKVAEQLPALIL
ASLHAHFGEWMDKDKQLEIAAQVLKSMSKRLDETSSKDLRDRLVETYQDASSVLVKYFSELPSGGGADPLRNIVKWRAAGVADTEKIYRQVT
IEFLDNPHACHASHLLGKTKRAYEFVRKTLGVPMHGKENLNEFKGEFEQWNTTGGYVSVIYASIRDGELYDMLGELEADL

>R9P5K0_Pseudozyma_hubeiensis
MAPTADVLPAAEASARPGLLVQPSDTKLRKASSFRTEQVVIDGYNLKIQGLVASARYGHVPVLDSSAATRKRIDDSVQSLIAKLDRGESIYG
INTGFGGSADSRTANTRALQLALLQMQQCGVLPVPSTFPTGEPSSAPFALPLTDTESSLIMPEAWVRGAIVVRLSSLMRGHSGVRWEVLEKM
QKLFLENNVTPVVPVRSSISASGDLSPLSYVAGALAGQRGIYCFVTDQRGQRVRVTADEACRMHNITPVQYEPKEALGLLNGTAFSASVAGL
ATYEAEKLAALTQLTTAMAVEALKGTDASFAPFIHEIARPHPGQIKSAKFIRALLSGSKLALHLEDEKHVLFSEDNGTLRQDRYTLRTASQW
VGPGLEDIENAKRSVDIEINSTTDNPMIDPYDGDGRIHHGGNFQAMAMTNAVEKIRLALCAMGKMTFQQMTELVNPAMNRGLPANLASTPDL
SLNFHAKGIDIALASVTSELMFLGNPVSTHVQSAEMANQAINSLALISGRQTLQAVECLSMIQAWSLYILCQAYDIRALQHKVAEQLPALIL
GSLRVHFGEWMDEAKQQEIAVLVLKSMSKRLDETSSKDLRDRLVETYQDASSVLVKYFSELPSGGGADPLRNIVKWRAAGVLDTEAIYRNVT
VEFLDNPHACHASHLLGKTKRAYEFVRKTLGVPMHGKENLNEFRGEFEQWNTTGGYVSVIYASIRDGELYSMLGELERDL

>E6ZJF9_Sporisorium_reilianum_SRZ2
MAPTADVLPAVEASPRPGLLVQPSDTKIRKASSFRTEQVVIDGNNLKIQGLVASARYGHVPILDASPATRKRIDDSVHSLLAKLDRGESIYG
INTGFGGSADSRTANTRALQLALLQMQQCGVLPVPSSFPTGEPSSAPFALPLTDTESSLIMPEAWVRGAIVVRLSSLMRGHSGVRWEVLDKM
QRLFLENNVTPVVPVRSSISASGDLCPLSYVAGALAGQRGIYCFVTDNHGQRVKVTADEACRMHNITPVQYEPKEALGLLNGTAFSASVAGL
ATYEADKLAALTQLTTAMAVEALKGTDASFAPFIHEVARPHPGQIKSAKFIRALLSGSKLAEHLENEKHVLFSEDNGTLRQDRYTLRTASQW
VGPGLEDIENAKRSVDIEINSTTDNPMIDPYDGDGRIHHGGNFQAMAMTNAVEKIRLALCAMGKMTFQQMTELVNPAMNRGLPANLASTPDL
SLNFHAKGIDIALASVTSELMFLGNPVSTHVQSAEMANQAINSLALISGRQTLQAVECLSMIQAWSLYLLCQALDIRALQHKVAEQLPALLL
ASLHAHFGEWMDNDTQLAIAAQVLKSMSRRLDETSSKDLRDRLVETYQDASSVLVKYFSELPSGGGADPLRNIVKWRAAGVADTEKIYRDVT
IEFLDNPHACHASHLLGKTRRAYEFVRKTLGVPMHGKENLNEFKGEFEQWNTTGGYVSVIYASIRDGELYNMLSQLEGDL

>I2G3P6_Ustilago_hordei
MAPTAHVLPMPIPTETRPSLAVQPSDTKLRKASSFRTEQVVIDGNNLKIQGLVASARYGHVPVLDSSPAIRKRIDDSVQSLIAKLDRGESIY
GINTGFGGSADSRTANTRALQLALLQMQQCGVLPVPSTFPTGEPSSAPFALPLTDTETSLVMPEAWVRGAIVVRLSSLMRGHSGVRWQVLEK
MQKLFLQNNVTPVVPVRSSISASGDLSPLSYVAGALAGQKGIYCWITDAKSGQRVKVTADEACRMYAIEPVQYEPKEALGLLNGTAFSASVA
ALATYEAEKLANLTQLTTAMAVEALKGTDASFAPFIHQVARPHPGQIKSARFIRALLSGSQLAEHLENEKHVLFSEDNGTLRQDRYTLRTAS
QWVGPGLEDIENAKRSVDIEINSTTDNPMIDPYDGDGRIHHGGNFQAMAMTNAVEKIRLALCAMGKMTFQQMTELVNPAMNRGLPANLASTP
DLSLNFHAKGIDIALASVTSELMFLGNPVSTHVQSAEMANQAINSLALISGRMTLQAVECLSMIQAWSLYLLCQALDIRALQHKVAEQLPAL
ILASLNSHFGEWMDEAKQAEIAKLVLKQMSKRLDETSSKDLRDRLVETYQDASSVLVKYFSELPSGGGADPLRNIVKWRAAGVADTEKIYRD
VTVEFLDNPYACHASHLLGKTKRAYEFVRKTLGVPMHGKENLNEFKGEFSQWNTTGGYVSVIYASIRDGELYSMLSELEKDLQL

>B2J528_Nostoc_punctiforme
MNITSLQQNITRSWQIPFTNSSDSIVTVGDRNLTIDEVVNVARHGTQVRLTDNADVIRGVQASCDYINNAVETAQPIYGVTSGFGGMADVVI
SREQAAELQTNLIWFLKSGAGNKLSLADVRAAMLLRANSHLYGASGIRLELIQRIETFLNAGVTPHVYEFGSIGASGDLVPLSYITGALIGL
DPSFTVDFDGKEMDAVTALSRLGLPKLQLQPKEGLAMMNGTSVMTGIAANCVYDAKVLLALTMGVHALAIQGLYGTNQSFHPFIHQCKPHPG
QLWTADQMFSLLKDSSLVREELDGKHEYRGKDLIQDRYSLRCLAQFIGPIVDGVSEITKQIEVEMNSVTDNPLIDVENQVSYHGGNFLGQYV
GVTMDRLRYYIGLLAKHIDVQIALLVSPEFSNGLPPSLVGNSDRKVNMGLKGLQISGNSIMPLLSFYGNSLADRFPTHAEQFNQNINSQGYI
SANLTRRSVDIFQNYMAIALMFGVQAVDLRTYKMKGHYDARTCLSPNTVQLYTAVCEVVGKPLTSVRPYIWNDNEQCLDEHIARISADIAGG
GLIVQAVEHIFSSLKST

>P11544_Rhodosporidium_toruloides
MAPSLDSISHSFANGVASAKQAVNGASTNLAVAGSHLPTTQVTQVDIVEKMLAAPTDSTLELDGYSLNLGDVVSAARKGRPVRVKDSDEIRS
KIDKSVEFLRSQLSMSVYGVTTGFGGSADTRTEDAISLQKALLEHQLCGVLPSSFDSFRLGRGLENSLPLEVVRGAMTIRVNSLTRGHSAVR
LVVLEALTNFLNHGITPIVPLRGTISASGDLSPLSYIAAAISGHPDSKVHVVHEGKEKILYAREAMALFNLEPVVLGPKEGLGLVNGTAVSA
SMATLALHDAHMLSLLSQSLTAMTVEAMVGHAGSFHPFLHDVTRPHPTQIEVAGNIRKLLEGSRFAVHHEEEVKVKDDEGILRQDRYPLRTS
PQWLGPLVSDLIHAHAVLTIEAGQSTTDNPLIDVENKTSHHGGNFQAAAVANTMEKTRLGLAQIGKLNFTQLTEMLNAGMNRGLPSCLAAED
PSLSYHCKGLDIAAAAYTSELGHLANPVTTHVQPAEMANQAVNSLALISARRTTESNDVLSLLLATHLYCVLQAIDLRAIEFEFKKQFGPAI
VSLIDQHFGSAMTGSNLRDELVEKVNKTLAKRLEQTNSYDLVPRWHDAFSFAAGTVVEVLSSTSLSLAAVNAWKVAAAESAISLTRQVRETF
WSAASTSSPALSYLSPRTQILYAFVREELGVKARRGDVFLGKQEVTIGSNVSKIYEAIKSGRINNVLLKMLA

>Q87V42_Pseudomonas_syringae
MTNQVPDPIMFGERALCIEDVLALANRQAPSALQGDDEFRARIARGAQFLDSLLSKEGVIYGVTTGYGDSCVVAVPLEHVEALPRYLYTFHG
CGLGKLLDAQATRAVLAARLQSLCQGVSGVRVELLERLQAFIDQDVLPLIPEEGSVGASGDLTPLSYVAATLSGEREVMFRGERRLASDVHR
ELGWTPLVLRPKEALALMNGTAVMTGIACLAFARADYLLQLATRITAMNVVALQGNPEHFDERLFAAKPHPGQMQVAAWLRQDLAIDAPTAP
LHRLQDRYSLRCAPHVLGVLADSLNWLRSFIEIELNSANDNPIIDAEAERVLHGGHFYGGHIAFAMDSLKTLVANVADLLDRQLALLVDERY
NHGLPSNLSGASAERAMLNHGFKAVQIGTSAWTAEALKNTMPASVFSRSTECHNQDKVSMGTIAARDAIRVLELTEQVAAATLIAANQGVWL
RSKGADARPLPPALASMHAELGEDFAPVIEDRALESELRLCLKHIANRRWRLHAQ

>A0A099SNC3_Pseudomonas_syringae
MTNQVPDPIMFGERALCIEDVLALANRQAPSALQGDDEFRARIARGAQFLDSLLSKEGVIYGVTTGYGDSCVVAVPLEHVEALPRYLYTFHG
CGLGKLLDAQATRAVLAARLQSLCQGVSGVRVELLERLQAFIDQDVLPLIPEEGSVGASGDLTPLSYVAATLSGEREVMFRGERRLASDVHR
ELGWTPLVLRPKEALALMNGTAVMTGIACLAFARADYLLQLATRITAMNVVALQGNPEHFDERLFAAKPHPGQMQVAAWLRQDLAIDAPTAP

LHRLQDRYSLRCAPHVLGVLADSLNWLRSFIEIELNSANDNPIIDAEAERVLHGGHFYGGHIAFAMDSLKTLVANVADLLDRQLALLVDERY
NHGLPSNLSGASAERAMLNHGFKAVQIGTSAWTAEALKNTMPASVFSRSTECHNQDKVSMGTIAARDAIRVLELTEQVAAATLIAANQGVWL
RSKGADARPLPPALASMHAELGEDFAPVIEDRALESELRLCLKHIANRRWRLHAQ

>A0A0W0KF34_Pseudomonas_syringae
MTNQVPDPIMFGERALCIEDVLALANRQAPSALQGDDEFRARIARGAQFLDSLLSKEGVIYGVTTGYGDSCVVAVPLEHVEALPRYLYTFHG
CGLGKLLDAQATRAVLAARLQSLCQGVSGVRVELLERLQAFIDQDVLPLIPEEGSVGASGDLTPLSYVAATLSGEREVMFRGERRLASDVHR
ELGWTPLVLRPKEALALMNGTAVMTGIACLAFARADYLLQLATRITAMNVVALQGNPEHFDERLFAAKPHPGQMQVAAWLRQDLAIDAPTAP
LHRLQDRYSLRCAPHVLGVLADSLNWLRSFIEIELNSANDNPIIDAEAERVLHGGHFYGGHIAFAMDSLKTLVANVADLLDRQLALLVDERY
NHGLPSNLSGASAERAMLNHGFKAVQIGTSAWTAEALKNTMPASVFSRSTECHNQDKVSMGTIAARDAIRVLELTEQVAAATLIAANQGVWL
RSKGADARPLPPALASMHAELGEDFAPVIEDRALESELRLCLKHIANRRWRLHAQ

>Q8GMG0_Streptomyces_globisporus
MALTQVETEIVPVSVDGETLTVEAVRRVAEERATVDVPAESIAKAQKSREIFEGIAEQNIPIYGVTTGYGEMIYMQVDKSKEVELQTNLVRS
HSAGVGPLFAEDEARAIVAARLNTLAKGHSAVRPIILERLAQYLNEGITPAIPEIGSLGASGDLAPLSHVASTLIGEGYVLRDGRPVETAQV
LAERGIEPLELRFKEGLALINGTSGMTGLGSLVVGRALEQAQQAEIVTALLIEAVRGSTSPFLAEGHDIARPHEGQIDTAANMRALMRGSGL
TVEHADLRRELQKDKEAGKDVQRSEIYLQKAYSLRAIPQVVGAVRDTLYHARHKLRIELNSANDNPLFFEGKEIFHGANFHGQPIAFAMDFV
TIALTQLGVLAERQINRVLNRHLSYGLPEFLVSGDPGLHSGFAGAQYPATALVAENRTIGPASTQSVPSNGDNQDVVSMGLISARNARRVLS
NNNKILAVEYLAAAQAVDISGRFDGLSPAAKATYEAVRRLVPTLGVDRYMADDIELVADALSRGEFLRAIARETDIQLR

>P21310_Pseudomonas_putida
MTELTLKPGTLTLAQLRAIHAAPVRLQLDASAAPAIDASVACVEQIIAEDRTAYGINTGFGLLASTRIASHDLENLQRSLVLSHAAGIGAPL
DDDLVRLIMVLKINSLSRGFSGIRRKVIDALIALVNAEVYPHIPLKGSVGASGDLAPLAHMSLVLLGEGKARYKGQWLSATEALAVAGLEPL
TLAAKEGLALLNGTQASTAYALRGLFYAEDLYAAAIACGGLSVEAVLGSRSPFDARIHEARGQRGQIDTAACFRDLLGDSSEVSLSHKNCDK
VQDPYSLRCQPQVMGACLTQLRQAAEVLGIEANAVSDNPLVFAAEGDVISGGNFHAEPVAMAADNLALAIAEIGSLSERRISLMMDKHMSQL
PPFLVENGGVNSGFMIAQVTAAALASENKALSHPHSVDSLPTSANQEDHVSMAPAAGKRLWEMAENTRGVLAIEWLGACQGLDLRKGLKTSA
KLEKARQALRSEVAHYDRDRFFAPDIEKAVELLAKGSLTGLLPAGVLPSL

>Q3IWB0_Rhodobacter_sphaeroides
MLAMSPPKPAVELDRHIDLDQAHAVASGGARIVLAPPARDRCRASEARLGAVIREARHVYGLTTGFGPLANRLISGENVRTLQANLVHHLAS
GVGPVLDWTTARAMVLARLVSIAQGASGASEGTIARLIDLLNSELAPAVPSRGTVGASGDLTPLAHMVLCLQGRGDFLDRDGTRLDGAEGLR
RGRLQPLDLSHRDALALVNGTSAMTGIALVNAHACRHLGNWAVALTALLAECLRGRTEAWAAALSDLRPHPGQKDAAARLRARVDGSARVVR
HVIAERRLDAGDIGTEPEAGQDAYSLRCAPQVLGAGFDTLAWHDRVLTIELNAVTDNPVFPPDGSVPALHGGNFMGQHVALTSDALATAVTV
LAGLAERQIARLTDERLNRGLPPFLHRGPAGLNSGFMGAQVTATALLAEMRATGPASIHSISTNAANQDVVSLGTIAARLCREKIDRWAEIL
AILALCLAQAAELRCGSGLDGVSPAGKKLVQALREQFPPLETDRPLGQEIAALATHLLQQSPV

>Q9SS45_Arabidopsis_thaliana
MELCNQNNHITAVSGDPLNWNATAEALKGSHLDEVKRMVKEYRKEAVKLGGETLTIGQVAAVARGGGGSTVELAEEARAGVKASSEWVMESM
NRGTDSYGVTTGFGATSHRRTKQGGALQNELIRFLNAGIFGPGAGDTSHTLPKPTTRAAMLVRVNTLLQGYSGIRFEILEAITKLLNHEITP
CLPLRGTITASGDLVPLSYIAGLLTGRPNSKAVGPSGETLTASEAFKLAGVSSFFELQPKEGLALVNGTAVGSGLASTVLFDANILAVLSEV
MSAMFAEVMQGKPEFTDHLTHKLKHHPGQIEAAAIMEHILDGSSYVKEAQLLHEMDPLQKPKQDRYALRTSPQWLGPQIEVIRAATKMIERE
INSVNDNPLIDVSRNKALHGGNFQGTPIGVAMDNSRLAIASIGKLMFAQFSELVNDFYNNGLPSNLSGGRNPSLDYGFKGAEIAMASYCSEL
QFLANPVTNHVQSAEQHNQDVNSLGLISSRKTAEAVDILKLMSTTYLVALCQAVDLRHLEENLKKAVKSAVSQVAKRVLTVGANGELHPSRF
TERDVLQVVDREYVFSYADDPCSLTYPLMQKLRHILVDHALADPEREANSATSVFHKIGAFEAELKLLLPKEVERVRVEYEEGTSAIANRIK
ECRSYPLYRFVRDELNTELLTGENVRSPGEEFDKVFLAISDGKLIDPLLECLKEWNGAPVSIC

>P45729_Petroselinum_crispum
MAYVNGTTNGHANGNGLDLCMKKEDPLNWGVAAEALTGSHLDEVKRMVAEYRKPVVKLEGETLTISQVAAISARDDSGVKVELSEEARAGVK
ASSDWVMDSMNKGTDSYGVTTGFGATSHRRTKQGGALQKELIRFLNAGIFGSGAEAGNNTLPHSATRAAMLVRINTLLQGYSGIRFEILEAI
TKFLNHNITPCLPLRGTITASGDLVPLSYIAGLLTGRPNSKAVGPTGVTLSPEEAFKLAGVEGGFFELQPKEGLALVNGTAVGSGMASMVLF
EANILAVLAEVMSAIFAEVMQGKPEFTDHLTHKLKHHPGQIEAAAIMEHILDGSAYVKAAQKLHEMDPLQKPKQDRYALRTSPQWLGPQIEV
IRSSTKMIEREINSVNDNPLIDVSRNKAIHGGNFQGSPIGVSMDNTRLAIAAIGKLMFAQFSELVNDFYNNGLPSNLSGGRNPSLDYGFKGA
EIAMASYCSELQFLANPVTNHVQSAEQHNQDVNSLGLISSRKTSEAVEILKLMSTTFLVGLCQAIDLRHLEENLKSTVKNTVSQVAKRVLTM
GVNGELHPSRFCEKDLLRVVDREYIFAYIDDPCSATYPLMQKLRETLVEHALNNGDKERNLSTSIFQKIAAFEDELKALLPKEVETARAALE
SGNPAIPNRIKECRSYPLYKFVREELGTEYLTGEKVRSPGEEFEKVFTAMSKGEIIDPLLECLESWNGAPLPIC

>P24481_Petroselinum_crispum
MENGNGATTNGHVNGNGMDFCMKTEDPLYWGIAAEAMTGSHLDEVKKMVAEYRKPVVKLGGETLTISQVAAISARDGSGVTVELSEAARAGV
KASSDWVMDSMNKGTDSYGVTTGFGATSHRRTKQGGALQKELIRFLNAGIFGNGSDNTLPHSATRAAMLVRINTLLQGYSGIRFEILEAITK
FLNQNITPCLPLRGTITASGDLVPLSYIAGLLTGRPNSKAVGPTGVILSPEEAFKLAGVEGGFFELQPKEGLALVNGTAVGSGMASMVLFEA
NILAVLAEVMSAIFAEVMQGKPEFTDHLTHKLKHHPGQIEAAAIMEHILDGSAYVKAAQKLHEMDPLQKPKQDRYALRTSPQWLGPQIEVIR
SSTKMIEREINSVNDNPLIDVSRNKAIHGGNFQGTPIGVSMDNTRLAIAAIGKLMFAQFSELVNDFYNNGLPSNLSGGRNPSLDYGFKGAEI
AMASYCSELQFLANPVTNHVQSAEQHNQDVNSLGLISSRKTSEAVEILKLMSTTFLVGLCQAIDLRHLEENLKSTVKNTVSSVAKRVLTMGV
NGELHPSRFCEKDLLRVVDREYIFAYIDDPCSATYPLMQKLRQTLVEHALKNGDNERNLSTSIFQKIATFEDELKALLPKEVESARAALESG
NPAIPNRIEECRSYPLYKFVRKELGTEYLTGEKVTSPGEEFEKVFIAMSKGEIIDPLLECLESWNGAPLPIC

>P45728_Petroselinum_crispum
MENGNGAITNGHVNGNGMDFCMKTEDPLYWGIAAEAMTGSHLDEVKKMVAEYRKPVVKLGGETLTISQVAAISARDGSGVTVELSEAARAGV
KASSDWVMDSMNKGTDSYGVTTGFGATSHRRTKQGGALQKELIRFLNAGIFGNGSDNTLPHSATRAAMLVRINTLLQGYSGIRFEILEAITK
FLNQNITPCLPLRGTITASGDLVPLSYIAGLLTGRPNSKAVGPTGVILSPEEAFKLAGVEGGFFELQPKEGLALVNGTAVGSGMASMVLFEA
NILAVLAEVMSAIFAEVMQGKPEFTDHLTHKLKHHPGQIEAAAIMEHILDGSAYVKAAQKLHEMDPLQKPKQDRYALRTSPQWLGPQIEVIR

SSTKMIEREINSVNDNPLIDVSRNKAIHGGNFQGTPIGMSMDNTRLAIAAIGKLMFAQFSELVNDFYNNGLPSNLSGGRNPSLDYGFKGAEI
AMASYCSELQFLANPVTNHVQSAEQHNQDVNSLGLISSRKTSEAVEILKLMSTTFLVGLCQAIDLRHLEENLKSTVKNTVSSVAKRVLTMGV
NGELHPSRFCEKDLLRFVDREYIFAYIDDPCSATYPLMQKLRQTLVEHALKNGDNERNMNTSIFQKIATFEDELKALLPKEVESARAALESG
NPAIPNRIEECRSYPLYKFVRKELGIEYLTGEKVTSPGEEFDKVFIAMSKGEIIDPLLECLESWNGAPLPIC

>P35510_Arabidopsis_thaliana
MEINGAHKSNGGGVDAMLCGGDIKTKNMVINAEDPLNWGAAAEQMKGSHLDEVKRMVAEFRKPVVNLGGETLTIGQVAAISTIGNSVKVELS
ETARAGVNASSDWVMESMNKGTDSYGVTTGFGATSHRRTKNGVALQKELIRFLNAGIFGSTKETSHTLPHSATRAAMLVRINTLLQGFSGIR
FEILEAITSFLNNNITPSLPLRGTITASGDLVPLSYIAGLLTGRPNSKATGPNGEALTAEEAFKLAGISSGFFDLQPKEGLALVNGTAVGSG
MASMVLFETNVLSVLAEILSAVFAEVMSGKPEFTDHLTHRLKHHPGQIEAAAIMEHILDGSSYMKLAQKLHEMDPLQKPKQDRYALRTSPQW
LGPQIEVIRYATKSIEREINSVNDNPLIDVSRNKAIHGGNFQGTPIGVSMDNTRLAIAAIGKLMFAQFSELVNDFYNNGLPSNLTASRNPSL
DYGFKGAEIAMASYCSELQYLANPVTSHVQSAEQHNQDVNSLGLISSRKTSEAVDILKLMSTTFLVAICQAVDLRHLEENLRQTVKNTVSQV
AKKVLTTGVNGELHPSRFCEKDLLKVVDREQVYTYADDPCSATYPLIQKLRQVIVDHALINGESEKNAVTSIFHKIGAFEEELKAVLPKEVE
AARAAYDNGTSAIPNRIKECRSYPLYRFVREELGTELLTGEKVTSPGEEFDKVFTAICEGKIIDPMMECLNEWNGAPIPIC

>P45724_Arabidopsis_thaliana
MDQIEAMLCGGGEKTKVAVTTKTLADPLNWGLAADQMKGSHLDEVKKMVEEYRRPVVNLGGETLTIGQVAAISTVGGSVKVELAETSRAGVK
ASSDWVMESMNKGTDSYGVTTGFGATSHRRTKNGTALQTELIRFLNAGIFGNTKETCHTLPQSATRAAMLVRVNTLLQGYSGIRFEILEAIT
SLLNHNISPSLPLRGTITASGDLVPLSYIAGLLTGRPNSKATGPDGESLTAKEAFEKAGISTGFFDLQPKEGLALVNGTAVGSGMASMVLFE
ANVQAVLAEVLSAIFAEVMSGKPEFTDHLTHRLKHHPGQIEAAAIMEHILDGSSYMKLAQKVHEMDPLQKPKQDRYALRTSPQWLGPQIEVI
RQATKSIEREINSVNDNPLIDVSRNKAIHGGNFQGTPIGVSMDNTRLAIAAIGKLMFAQFSELVNDFYNNGLPSNLTASSNPSLDYGFKGAE
IAMASYCSELQYLANPVTSHVQSAEQHNQDVNSLGLISSRKTSEAVDILKLMSTTFLVGICQAVDLRHLEENLRQTVKNTVSQVAKKVLTTG
INGELHPSRFCEKDLLKVVDREQVFTYVDDPCSATYPLMQRLRQVIVDHALSNGETEKNAVTSIFQKIGAFEEELKAVLPKEVEAARAAYGN
GTAPIPNRIKECRSYPLYRFVREELGTKLLTGEKVVSPGEEFDKVFTAMCEGKLIDPLMDCLKEWNGAPIPIC

>B2Z6R0_Populus_trichocarpa
METVTKNGYQNGSLESLCVNQLDPLSWGVAAEAMKGSHLDEVKRMVADYRKPVVKLGGETLTIAQVASIAGHDTGDVKVELSESARPGVKAS
SDWVMDSMDKGTDSYGVTTGFGATSHRRTKQGGALQKELIRFLNAGIFGNGTETCHTLPHSATRAAMLVRINTLLQGYSGIRFEILEAITRL
LNNNITPCLPLRGTITASGDLVPLSYIAGLLTGRPNSKATGPTGEVLDAAEAFKAAGIESGFFELQPKEGLALVNGTAVGSGLASMVLFETN
VLAVLSELLSAIFAEVMNGKPEFTDHLTHKLKHHPGQIEAAAIMEHILDGSAYMKAAKKLHETDPLQKPKQDRYALRTSPQWLGPQIEVIRF
STKSIEREINSVNDNPLIDVSRNKAIHGGNFQGTPIGVSMDNVRLAIASIGKLLFAQFSELVNDFYNNGLPSNLTASRNPSLDYGFKGAEIA
MASYCSELQYLANPVTTHVQSAEQHNQDVNSLGLISSRKTAEAVDILKLMSTTFLVALCQAIDLRHLEENLKSAVKNTVSQVSKRVLTTGAN
GELHPSRFCEKELLKVVDREYVFAYVDDPCSATYPLMQKLRQVFVDHALENGENEKNFSTSVFQKIEAFEEELKALLPKEVESARAAYDSGN
SAIDNKIKECRSYPLYKFVREELGTVLLTGEKVQSPGEEFDKVFTAMCQGKIIDPMLECLGEWNGSPLPIC

>B2Z6R1_Populus_trichocarpa
METITKNGYQNGSSESLCTQRDPLSWGVAAEAMKGSHLDEVKRMVAEYRKPVVNLAGQTLTIAQVASIAGHDASNVKVELSESARPRVKASS
DWVMDSMDKGTDSYGVTTGFGATSHRRTKQGGALQKELIRFLNAGIFGNGTETCHTLPHSATRAAMLVRINTLLQGYSGIRFEILEAITKLL
NNNITPCLPLRGTITASGDLVPLSYIAGLLTGRPNSKATGPNGEVLDAVEAFKAAGIDSGFFELQPKEGLALVNGTAVGSGLASMVLFETNV
LAVLSELISAIFAEVMNGKPEFTDHLTHKLKHHPGQIEAAAIMEHILDGSAYMKAAKKLHEMDPLQKPKQDRYALRTSPQWLGPQIEVIRFS
TKSIEREINSVNDNPLIDVSRNKALHGGNFQGTPIGVSMDNVRLAIASIGKLLFAQFSELVNDFYNNGLPSNLTASRNPSLDYGFKGAEIAM
ASYCSELQYLANPVTSHVQSAEQHNQDVNSLGLISSRKTAESVDILKLMSTTFLVALCQAIDLRHLEENLRSAVKNTVSHVSKRVLTTGANG
ELHPSRFCEKELLKVVDREDVFAYADDPCSATYPLMQKLRQVLVDHALANGENEKNTSTSVFQKITAFEEELKALLPKEVESARAAYDSGNS
AIENKIKECRSYPLYKFVREELGTGLLTGEKVRSPGEEFDKVFTAMCQGKIIDPMLECLGEWNGAPLPIC

>A3AVL7_Oryza_sativa_japonica
MASQTADAHGVRRERPAVVGQGGAGDDGEPPGRGEAHGGAVTGGRGEDRGVQPPRRPGGRRLRGQGRLRRGGRAGRGGPPPRQGQQRVGSST
ASPTAATSTASPPASAGTSNRRTKDGQALQVELLRHLNAGIFGNGSDGNSLPSEVSRAAMLVRINTLLQGYSGIRFEILEAITKLINTGVSP
CLPLRGTITASGDLVPLSYIAGLITGRPNAQAVTVDGKKVDAAEAFKIAGIQGGFFRLEPKEGLAIVNGTSVGSALAAMVLYDANVLAVLSE
VLSAVFCEVMNGKPEYTDHLTHKLKHHPGSIEAAAIMEHILAGSAFMPHAQKVNEVDPLLKPKQDRYALRTSPQWLGPQIEVIRAATKSIER
EVNSVNDNPVIDVHRGKALHGGNFQGTPIGVSMDNTRLAIANIGKLMFAQFSELVNEFYNNGLTSNLAGSRNPSLDYGFKGTEIAMASYCSE
LQFLANPVTNHVQSAEQHNQDVNSLGLVSARKTAEAVDILKLMSSTYLVALCQAVDLRHLEENLKSAVKNCVTTVAKKVLTTGPAGGLHSAR
FSEKALLTAIDREAVYSYADDPCSANYPLMTKIRAVLVEHALANGPAEKDDGSSVFSKITAFEEELREALPREMEAARVAFETGTAPITNRI
KESRSFPLYRFVREELGCVYLTGEKLKSPGEECNKVFLAISERKLIDPMLECLKEWNGEPLPIC

>Q84VE0_Oryza_sativa_japonica
MVAQSREAVVKIEGSSLRVGQVAAVSAAKDASGVVVELDEEARPRVKASSEWILNCIAHGGDIYGVTTGFGGTSHRRTKDGQALQVELLRHL
NAGIFGNGSDGNSLPSEVSRAAMLVRINTLLQGYSGIRFEILEAITKLINTGVSPCLPLRGTITASGDLVPLSYIAGLITGRPNAQAVTVDG
KKVDAAEAFKIAGIQGGFFRLEPKEGLAIVNGTSVGSALAAMVLYDANVLAVLSEVLSAVFCEVMNGKPEYTDHLTHKLKHHPGSIEAAAIM
EHILAGSAFMPHAQKVNEVDPLLKPKQDRYALRTSPQWLGPQIEVIRAATKSIEREVNSVNDNPVIDVHRGKALHGGNFQGTPIGVSMDNTR
LAIANIGKLMFAQFSELVNEFYNNGLTSNLAGSRNPSLDYGFKGTEIAMASYCSELQFLANPVTNHVQSAEQHNQDVNSLGLVSARKTAEAV
DILKLMSSTYLVALCQAVDLRHLEENLKSAVKNCVTTVAKKVLTTGPAGGLHSARFSEKALLTAIDREAVYSYADDPCSANYPLMTKIRAVL
VEHALANGPAEKDDGSSVFSKITAFEEELREALPREMEAARVAFETGTAPITNRIKESRSFPLYRFVREELGCVYLTGEKLKSPGEECNKVF
LAISERKLIDPMLECLKEWNGEPLPIC

>K3YDF1_Setaria_italica
MACSTAIVTSDPLNWGKAAAELTGSHLDEVRRMVAQSREPVVRVDGSRLHVGQVAAVAAAKDASGVAVELDEEARLRVKASSEWVLSCIENG
GDIYGVTTGFGGNSHRRTKDGHALQVELLRYLNAGIFGTGSDGHTLPSQVSRAAMLVRINALMQGYSGIRFEILEAIAKLINTGVSPCLPLR
GSITASGDLVPLSYIAGLITGRPNAQAVTVDGRKVDAAEAFKVAGIEGGFFKLNPKEGLAMVNGTSVGSALAAMVCFDANVLAVLAVVLSAV
FCEVMNGKPEYADHLTHKLKHHPGSIEAAAIMEHILDGSSLMKHAKEVNAMDPLLKPKQDQYALRTSPQWLGPQIEVIRAATKAIEREINSV
SDNPVIDVHRGKALHGGNFQGTPIGVSMDNARLAVASIGRLMFAQFTELVIDFYNNGLPSNLAGSRNLSLDFGLKGAEIAMASYCSELQYLA

NPVTNHVQSAEQHTQDVNSLGLISARKTAEAVEILKLMSSTFMIALCQAVDLRHLEENLKSAVKNCVKTVALKVLTTSPDGEHCSARFSEKA
LLAAIDRKAVYSYYDDPCSASSSLMMTIRAVLVDHALANGEAENEARAPIFSKITKFEEELREALPREMEKTRVAFETGTAPIGNRIKESRS
YPLYRFIREDLGAVYLTGEKLKSAGEECNKVFLALSEGKLIDPMLGCLKEWNGEPLPIC

>K3ZR63_Setaria_italica
MACNTAIVTSDPLNWGKAAAELTGSHLDEVRRMVAQSREPIVRVDGSRLHVGKVAAVAAAKDASGVAVELDEEARLRVRSSSEWVLSCIENG
GDIYGVTTGFGGNSHRRTKDGHALQVELLRYLNAGIFGTGSDGHTLPSQVSRAAMLVRINALMQGYSGIRFEILEAIAKLINTGVSPCLPLR
GSITASGDLVPLSYIAGLITGRPNAQAVTVDGRKVDAAEAFKVAGIKGGFFKLNPKEGLAMVNGTSVGSALAAMVCFDANVLAVLAVVLSAV
FCEVMNGKPEYADHLTHKLKHHPGSIEAAAIMEHILDGSSLMKHAKEVNAMDPLLKPKQDRYALRTSPQWLGPQIEVIRAATKAIEREINSV
SDNPVIDVNRGKALHGGNFQGTPIGVSMDNARLAVASIGRLMFAQFTELVIDFYNNGLPSNLAGSRNLSLDFGLKGVEIAMASYCSELQYLA
NPVTNHVQSAEQHTQDVNSLGLISARKTAEAVEILKLMSSTFMIALCQAVDLRHLEENLKSAVKNCVKTVALKVLTTSPDGEHCSARFSEKT
LLAAIDRKAVYSYCDDPCSASSSLMMTIRAVLVDHALANGEAENEARAPIFSKITKFEEELREALPREMEKTRVAFETGTAPIGNRIKESRS
YPLYRFIREDLGAVYLTGEKLKSAGEECNKVFLALSEGKLIDPMLGCLKEWNGEPLPIC

>I1IBR5_Brachypodium_distachyon
MAGNGAISEKDPLNWGAAAAELTGSHLDEVKRMVAQFREPVVKIEGASLRVGQVAAVAQAKDAAGVSVELDEEARPRVKASSEWILSCLAAG
GDIYGVTTGFGGTSHRRTKDGPALQVELLRHLNAGIFGTGSDGHSLPAEVTRAAMLVRINTLLQGYSGIRFEILEAITKLLNTGVSPCLPLR
GTITASGDLVPLSYIAGLITGRPNAQATTADGRKVDAAEAFKVAGIEGGFFTLNPKEGLAIVNGTSVGSALAATVLFDCNVLAVLSEVLSAV
FCEVMNGKPEFTDHLTHKLKHHPGSIEAAAIMEHILAGSSFMSHAKKVNEIDPQLKPKQDRYALRTSPQWLGPQIEVIRSATKSIEREVNSV
NDNPVIDVHRGKALHGGNFQGTPIGVSMDNTRLAIANIGKLMFAQFSELVNEFYNNGLTSNLAGSRNPSLDYGFKGTEIAMASYCSELQYLA
NPVTNHVQSAEQHNQDVNSLGLVSARKTAEAVDILKLMSSTYMVALCQAVDLRHLEENIKASVKNCVTQVSKKVLTMNPTGDLSSARFSEKS
LLTAIDREAVFSYADDACSANYPLMQKLRAVLVDHALTSSGVDNAGESEATVFSKINKFEEELRAALPREIEAARVAFEKGTAPIPNLIKDS
RSFPLYRFVREELGCVYLTGEKLLSPGEECNKVFIGISQGKLIDPMLECLKEWNGEPLPINVV

>D5KS97_Bambusa_oldhamii
MAGNGPIVKDDPLNWGAAAAELTGSHFDEVKRMVAQFREPVIKIEGASLRVGQVAAVAQAKDVSGVAVELDEEARPRVKASSEWILNCLAHG
GDIYGVTTGFGGTSHRRTKDGPALQVELLRHLNAGIFGTGTDGHTLPSEVTRAAMLVRINTLLQGYSGIRFEILEAITKLINTGVTPCLPLR
GTITASGDLVPLSYIAGLITGRPNAQAVAPDGRKVDAAEAFKIAGIEGGFFKLNPKEGLAIVNGTSVGSALAATVLYDCNVLAVLSEVLSAV
FCEVMNGKPEYTDHLTHKLKHHPGSIEAAAIMEHILAGSSFMSHAKKVNEMDPLLKPKQDRYALRTSPQWLGPQIEVIRAATKSIEREVNSV
NDNPVIDVHRGKALHGGNFQGTPIGVSMDNTRLAIANIGKLMFAQFSELVNEFYNNGLTSNLAGSRNPSLDYGFKGTEIAMASYCSELQYLA
NPITNHVQSAEQHNQDVNSLGLVSARKTAEAVDILKLMSSTYMVALCQAVDLRHLEENIKSSVKNCVTQVAKKVLTMNPTGDLSSARFSEKN
LLTAIDREAVFTYADDPCSANYPLMQKLRAVLVDHALTSGDAEREPSVFSKITKFEEELRSALPREIEAARVAVADGTAPIANRIKESRSFP
VYRFVREELGCVYLTGEKLKSPGEECNKVFIGISQGKLIDPMLECLKEWNGEPLPIN

>P14717_Oryza_sativa_japonica
MAGNGPINKEDPLNWGAAAAEMAGSHLDEVKRMVAQFREPLVKIQGATLRVGQVAAVAQAKDAAGVAVELDEEARPRVKASSEWILNCIAHG
GDIYGVTTGFGGTSHRRTKDGPALQVELLRHLNAGIFGTGSDGHTLPSETVRAAMLVRINTLLQGYSGIRFEILEAITKLLNTGVTPCLPLR
GTITASGDLVPLSYIAGLITGRPNAQAISPDGRKVDAAEAFKLAGIEGGFFTLNPKEGLAIVNGTSVGSALAATVMFDANILAVLSEVLSAV
FCEVMNGKPEYTDHLTHKLKHHPGSIEAAAIMEHILAGSSFMSHAKKVNEMDPLLKPKQDRYALRTSPQWLGPQIEVIRAATKSIEREVNSV
NDNPVIDVHRGKALHGGNFQGTPIGVSMDNARLAIANIGKLMFAQFSELVNEFYNNGLTSNLAGSRNPSLDYGFKGTEIAMASYCSELQYLA
NPITNHVQSAEQHNQDVNSLGLVSARKTLEAVDILKLMTSTYIVALCQAVDLRHLEENIKSSVKNCVTQVAKKVLTMNPTGDLSSARFSEKN
LLTAIDREAVFSYADDPCSANYPLMQKLRAVLVEHALTSGDAEPEASVFSKITKFEEELRSALPREIEAARVAVANGTAPVANRIVESRSFP
LYRFVREELGCVFLTGEKLKSPGEECNKVFLGISQGKLIDPMLDCLKEWNGEPLPIN

>K3Y5M0_Setaria_italica
MASNTAILESDPLNWGKAAAEMAGSHLDEVKRMVAQFREPLVKIEGSSLRVGQVAAVAAAKDASGVAVELDEDARPRVKASSEWILDCIAHG
GDIYGVTTGFGGTSHRRTKDGPALQVELLRHLNAGIFGNGSDGHTLPSEVSRAAMLVRINTLLQGYSGIRFEILEAITKLINTGVSPCLPLR
GTITASGDLVPLSYIAGLITGRPNAQAVTVDGRKVDAAEAFKVAGIEGGFFKLNPKEGLAIVNGTSVGSALGAMVCFDANVLAVLSEVLSAV
FCEVMNGKPEYTDHLTHKLKHHPGSIEAAAIMEHILEGSSFMKHAKKVNELDPLLKPKQDRYALRTSPQWLGPQIEVIRAATKSIEREVNSV
NDNPVIDVHRGKALHGGNFQGTPIGVSMDNARLAIANIGKLMFAQFSELVNEFYNNGLTSNLAGSRNPSLDYGFKGTEIAMASYCSELQYLG
NPITNHVQSAEQHNQDVNSLGLVSARKTAEAIDILKLMSSTYMVALCQAIDLRHLEENIKTSVKNTVTQVAKKVLTMNPVGELSSARFSEKD
IITAIDREGVFTYAEDAASASLPLMQKLRAVLVDHALSSGDAEREPSVFSKITKFEEELRAVLPREVEAARVAVAEGTAPVANRIKDSRSFP
VYRFVREELGCVFLTGEKLKSPGEECNKVFIGISQGKLIDPMLECLKEWDGKPLPIC

>K3YQG1_Setaria_italica
MAGNGLIVENDPLNWGAAAAELAGSHLDEVKRMVAQARQPVVKIEGSTLRVGQVAAVASARDASGVAVELDEEARPRVKASSEWILDCIAHG
GDIYGVTTGFGGTSHRRTKDGPALQVELLRHLNAGIFGTGSDGHTLPSEVTRAAMLVRINTLLQGYSGIRFEILEAITKLLNSGVSPCLPLR
GTITASGDLVPLSYIAGLITGRPNAQAVTADGRKVDAAEAFKIAGIEGGFFKLNPKEGLAIVNGTSVGSALAATVLYDANVLAVLSEVLSAV
FCEVMNGKPEYTDHLTHKLKHHPGSIEAAAIMEHILEGSEFMKHAKKVNELDPLLKPKQDRYALRTSPQWLGPQIEVIRAATKSIEREVNSV
NDNPVIDVHRGKALHGGNFQGTPIGVSMDNARLAIANIGKLMFAQFSELVNEFYNNGLTSNLAGSRNPSLDYGFKGTEIAMASYCSELQYLG
NPITNHVQSAEQHNQDVNSLGLVSARKTAEAIDILKLMSSTYMVALCQAIDLRHLEENIKTSVKNTVTQVAKKVLTMNPTGDLSSARFSEKD
LITAIDREGVFTYAEDAASASLPLMQKLRAVLVDHAFSSGDEPSMFSKITKFEEELRAVLPQEVEAARVAVAEGTAAVENRIKDSRSFPLYR
FVREELGCVFLTGEKLKSPGEECNKVFIGISQGKLIDPMLECLKEWDGKPLPIN

>K3YGI0_Setaria_italica
MAGNGLIVENDPLNWGAAAAELAGSHLDEVKRMVAQARQPVVKIEGSTLRVGQVAAVAAAKDASGVAVELDEEARPRVKASSEWILDCIAHG
GDIYGVTTGFGGTSHRRTKDGPALQVELLRHLNAGIFGAGSDGHTLPSEVTRAAMLVRINTLLQGYSGIRFEILEAITKLLNSGVSPCLPLR
GTITASGDLVPLSYIAGLITGRPNAQAVTVDGRKVDAAEAFKVAGIEGGFFRLNPKEGLAIVNGTSVGSALAATVLYDANLLAVLSEVLSAV
FCEVMNGKPEYTDHLTHKLKHHPGSIEAAAIMEHILEGSAFMKHAKKVNELDPLLKPKQDRYALRTSPQWLGPQIEVIRAATKSIEREVNSV
NDNPVIDVHRGKALHGGNFQGTPIGVSMDNARLAIANIGKLMFAQFSELVNEFYNNGLTSNLAGSRNPSLDYGFKGTEIAMASYCSELQYLG
NPITNHVQSAEQHNQDVNSLGLVSARKTAEAIDILKLMSSTYIVALCQAIDLRHLEENIKTSVKNTVTQVAKKVLTMNPAGELSSARFSEKD

LLTAIDREGVFTYAEDAASASLPLMQKLRAVLVDHAFSSGDEPSVFSKITKFEEELRAVLPQEVEAARVAVAEGTAAVENRIKDSRSFPLYR
FVREELGCVFLTGEKLKSPGEECNKVFVGISQGKLVDPMLECLKEWDGKPLPIN

>B8A046_Zea_mays
MAGNGAIVESDPLNWGAAAAELAGSHLDEVKRMVAQARQPVVKIEGSTLRVGQVAAVASAKDASGVAVELDEEARPRVKASSEWILDCIAHG
GDIYGVTTGFGGTSHRRTKDGPALQVELLRHLNAGIFGTGSDGHTLPSEVTRAAMLVRINTLLQGYSGIRFEILEAITKLLNTGVSPCLPLR
GTITASGDLVPLSYIAGLITGRPNAQAVTVDGRKVDAAEAFKIAGIEGGFFKLNPKEGLAIVNGTSVGSALAATVMYDANVLAVLSEVLSAV
FCEVMNGKPEYTDHLTHKLKHHPGSIEAAAIMEHILDGSSFMKQAKKLNELDPLLKPKQDRYALRTSPQWLGPQIEVIRAATKSIEREVNSV
NDNPVIDVHRGKALHGGNFQGTPIGVSMDNARLAIANIGKLMFAQFSELVNEFYNNGLTSNLAGSRNPSLDYGFKGTEIAMASYCSELQYLG
NPITNHVQSAEQHNQDVNSLGLVSARKTAEAIDILKLMSSTYIVALCQAVDLRHLEENIKASVKNTVTQVAKKVLTMNPSGELSSARFSEKE
LISAIDREAVFTYAEDAASGSLPLMQKLRAVLVDHALSSGDAEREPSVFSKITRFEEELRAVLPQEVEAARVAVAEGTAPVANRIADSRSFP
LYRFVREELGCVFLTGERLKSPGEECNKVFVGISQGKLVDPMLECLKEWDGKPLPINVK

>I1IZQ0_Brachypodium_distachyon
MEYENGHAATYGDGLCVAAPLAPRADPLNWGKAAEELSGSHLDAVKRMVEEYRRPVVKMEGASLTIAQVAAVAAGAEARVELDESARGRVKE
SSDWVMNSMMNGTDSYGVTTGFGATSHRRTKEGGALQRELIRFLNAGAFGTGEDGHVLPAAATRAAMLVRVNTLLQGYSGIRFEILETIATL
LNANVTPCLPLRGTITASGDLVPLSYIAGLVTGRPNSVATAPDGRKVNAAEAFKIAGIQHGFFELQPKEGLAMVNGTAVGSGLASMVLFEAN
ILGVLAEVLSAVFCEVMNGKPEFTDHLTHKLKHHPGQIEAAAIMEHILEGSSYMMLAKKLGELDPLMKPKQDRYALRTSPQWLGPQIEVIRA
ATKSIEREINSVNDNPLIDVSRGKAIHGGNFQGTPIGVSMDNTRLAIAAIGKLMFAQFSELVNDFYNNGLPSNLSGGRNPSLDYGFKGAEIA
MASYCSELQFLGNPVTNHVQSAEQHNQDVNSLGLISSRKTAEAIDILKLMSSTFLVALCQAIDLRHLEENVRSAVKNCVTTVARKTLSTNVN
GHLHNARFCEKDLLLTIDREAVFAYADDPCSANYPLMQKMRAVLVEHALANGEAERDVETSVFAKLAAFEQELRAVLPKEVEAARAAVENGT
ATKQNRIAECRSYPLYRFVREELGTEYLTGEKTRSPGEEVDKVFVAMNQGKHIDALLECLKEWNGEPLPLC

>D3JYP7_Bambusa_oldhamii
MECENGHVAANVSDLCMAKPPRADPLNWGKAAEELSGSHLDAVKRMVDEYRRPVVRIEGASLTIAQVAAVAAGEARVELDESARGRVKESS
DWVMNSMMNGTDSYGVTTGFGATSHRRTKEGGALQRELIRFLNAGALDTGDDGHVLPAAATRAAMLVRINTLLQGYSGIRFEILETIAALLN
ANVTPCLPLRGTITASGDLVPLSYIAGLVTGRPNSVAVAPDGRKVNAADAFKIAGIQHGFFELQPKEGLAMVNGTAVGSGPASMVLFEANIL
GVLAEVLSAVFCEVMNGKPEYTDHLTHKLKHYPGQIEAAAIMEHTLEGSSYMMLAKKLGELDPLMKPKQDRYALRTSPQWLGPQIEVIRAAT
KSIEREINSVNDNPLIDVSRGKALHGGNFQGTPIGVSMDNTRLAIAAIGKLMFAQFSELVNDLYNNGLPSNLSGGRNPSLDYGFKGAEIAMA
SYCSELQFLANPVTNHVQSAEQHNQDVNSLGLISSRKTGEATDILKLMSSTFLIALCQAIDLRHLEENVKNAVKNCVTTVARKTLSTSATGD
LHNARFCEKDLLKAIDREAVFAYADDPCSANYPLMQKMRAVLVEHALANGEAESNVDTSVFAKVATFEEELRAMLPREVEAARAAVENGTAA
KQNGITECRSYPLYRFVREELGTEYLTGEKARSPGEEVNKVFVALNQGKHIDALLECLKEWNGEPLPIC

>I1IBR6_Brachypodium_distachyon
MECENGLVGSLNGEGLCMSAPPRAAADPLNWAKTAEELAGSHLEEVKKMVAQFRMPLVKIEGATLGIAQVAAVAAGAGEARVELDESARGRV
KESSDWVMNSMMNGTDSYGVTTGFGATSHRRTKEGGALQRELIRFLNAGAFGTGTDGHVLPAEATRAAMLVRINTLLQGYSGIRFEILEAIT
KLLNANVTPCLPLRGTITASGDLVPLSYIAGLITGRQNSVAVAPDGSKVSAAEAFKIAGIEHGFFELQPKEGLAMVNGTAVGSGLASTVLFE
ANIQAILAEVLSAVFCEVMTGKPEFTDHLTHKLKHHPGQIEAAAIMEHILEGSSYMKEAKKQGELDPLMKPKQDRYALRTSPQWLGPQIEVI
RFATKSIEREINSVNDNPLIDVSRGKAIHGGNFQGTPIGVSMDNTRLAIAAIGKLMFAQFSELVNDFYNNGLPSNLSGGRNPSLDYGFKGAE
IAMASYCSELQFLGNPVTNHVQSAEQHNQDVNSLGLISSRKTAEAIDILKIMSSTFLIALCQAIDLRHLEENMKTAVRNCVMQVAKKTLSMN
AMGGLHIARFCEKDLLTAIDREAVFAYADDPCSPNYPLMQKLRAVLIEHALANGDGERALETSIFAKVAEFEQNLRAALPKEVEAARASVEN
GTPLAPNRIKDCRSYPLYRFVREELGTEYLTGEKTRSPGEELNKVLVAMNQRKHIDPLLECLKEWNGEPLPLC

>B4FW68_Zea_mays
MESEAGLLVRSSLNGEGLCMPAPRADPLNWGKAAEGLSGSHLDEVKRMVAEFRDPLVKIQGASLSVAQVAAVAVGAGGGEARVELDESARER
VRASSDWVMGSMMNGTDSYGVTTGFGATSHRRTKEGGALQRELIRFLNAGAFGTGADGHVLPAEATRAAMLVRVNTLLQGYSGIRFEILEAI
AKLLNANVTPCLPLRGTITASGDLVPLSYIAGLITGRQNSVAVDPDGRKVGAAEAFKIAGIEHGFFELQPKEGLAMVNGTAVGSGLASTVLF
EANVLAVLAEVISAVFCEVMTGKPEFTDHLTHKLKHHPGQIEAAAVMEHILEGSSYMKLAKRLGELDPLMKPKQDRYALRTSPQWLGPQIEV
IRFATKSIEREINSVNDNPLIDVSRGKALHGGNFQGTPIGVSMDNTRLALAAIGKLMFAQFSELVNDYYNNGLPSNLSGGRNPSLDYGFKGA
EIAMASYCSELQFLGNPVTNHVQSAEQHNQDVNSLGLISSRKTAEAIDILKLMSSTFLIALCQAIDLRHLEENVKAAVKNCVTQVAKKSLSL
NARGGLHNARFCEKDLQTAIDREAVFAYADDPCSPNYALMQKLRAVLVEHALANGDAERDVDTSIFAKVAEFEQQVRAALPKEVEAARAAVE
NGSPLVPNRIKECRSYPLYRFVREEVGTKYLTGEKTRSPGEELNKVLVAINQRKHIDPLLECLKEWNGEPLPLC

>B6U0Z0_Zea_mays
MESEAGLLVRSSLNGEGLCMPAPRADPLNWGKAAEGLSGSHLDEVKRMVAEFRDPLVKIQGASLSVAQVAAVAVGAGGGEARVELDESARER
VRASSDWVMGSMMNGTDSYGVTTGFGATSHRRTKEGGALQRELIRFLNAGAFGTGADGHVLPAEATRAAMLVRVNTLLQGYSGIRFEILEAI
AKLLNANVTPCLPLRGTITASGDLVPLSYIAGLITGRQNSMAVAPDGRKVGAAEAFKIAGIEHGFFELQPKEGLAMVNGTAVGSGLASTVLF
EANVLAVLAEVISAVFCEVMTGKPEFTDHLTHKLKHHPGQIEAAAVMEHILEGSSYMKLAKRLGELDPLMKPKQDRYALRTSPQWLGPQIEV
IRFATKSIEREINSVNDNPLIDVSRGKALHGGNFQGTPIGVSMDNTRLALAAIGKLMFAQFSELVNDYYNNGLPSNLSGGRNPSLDYGFKGA
EIAMASYCSELQFLGDPVTNHVQSAEQHNQDVNSLGLISSRKTAEAIDILKLMSSTFLIALCQAIDLRHLEENVKAAVKNCVTQVAKKSLSL
NARGGLHNARFCEKDLQTAIDREAVFAYADDPCSPNYALMQKLRAVLVEHALANGDAERDVDTSIFAKVAEFEQQVRAALPKEVEAARAAVE
NGSPLVPNRIKECRSYPLYRFVREEVGTEYLTGEKTRSPGEELNKVLVAINQRKHIDPLLECLKEWNGEPLPLC

>K3YQC4_Setaria_italica
MECETGLVRSLHGDGLCMSAQAAAPRADPLNWGKAAEDLSGSHLDEVKRMVAEFREPLVKIQGASLSIAQVAAVAAGAGEARVELDESARER
VKASSDWVMNSMMNGTDSYGVTTGFGATSHRRTKEGGALQRELIRFLNAGAFGTGADGHVLPAEATRAAMLVRINTLLQGYSGIRFEILETI
AKLLNANVTPCLPLRGTITASGDLVPLSYIAGLITGRQNSVAVAPDGRKVDAAEAFKIAGIEHGFFELQPKEGLAMVNGTAVGSGLASTVLF
EANVLAIMAEVISAVFCEVMTGKPEFTDHLTHKLKHHPGQIEAAAIMEHILEGSSYMKLAKKLGELDPLMKPKQDRYALRTSPQWLGPQIEV
IRFATKSIEREINSVNDNPLIDVSRGKALHGGNFQGTPIGVSMDNTRLALAAIGKLMFAQFSELVNDFYNNGLPSNLSGGRNPSLDYGFKGA
EIAMASYCSELQFLGNPVTNHVQSAEQHNQDVNSLGLISSRKTAEAIEILKLMTSTFLIALCQAIDLRHLEENMKAAVKNCVTQVAKKTLSM

NAMGGLHIARFCEKDLQTAIDREAVFAYADDPCSPNYPLMQKLRAVLIEHALANGDAERVVETSIFAKVAEFEQQVRAALPKEVEAARAAVE
SGRPMVPNRIKECRSYPLYRFVREELGAEYLTGEKTRSPGEELNKVLVAINERKHIDPLLECLKEWNGEPLPLC

>C0LL35_Bambusa_oldhamii
MECENGQVASNGNGLCMATPRADPLNWGKAAEELMGSHLEEVKRMVAEYRQPVVKIEGASLRIAQVAAVAAGAGEAKVQLDDSARGRVKESS
DWVMNSMMNGTDSYGVTTGFGATSHRRTKEGGALQRELIRFLNAGAFGTGSDGHVLAAEATRAAMLVRINTLLQGYSGIRFEILEAIAKLLN
ANVTPCLPLRGTITASGDLVPLSYIAGLVTGRENSVAVAPDGRKVNAAEAFKIAGIQGGFFELQPKEGLAMVNGTAVGSGLASTVLFEANIL
AILAEVLSAVFCEVMNGKPEYTDHLTHKLKHHPGQIEAAAIMEHILEGSSYMKLAKKLGELDPLMKPKQDRYALRTSPQWLGPQIEVIRAAT
KSIEREINSVNDNPLIDVSRGKALHGGNFQGTPIGVSMDNTRLAIAAVGKLMFAQFSELVNDFYNNGLPSNLSGGRNPSLDYGFKGAEIAMA
SYCSELQFLGNPVTNHVQSAEQHNQDVNSLGLISSRKTAEAIDILKIMSSTFLVALCQAIDLRHIEENVKSAVKSCVMTVAKKTLSTNSTGD
LHVARFCEKDLLKEIDREAVFAYADDPCSPNYPLMKKMRNVLVERALANGMAEFNAETSVFAKVAQFEEELRATLPRAVEAARAAVENGTAA
TPNRITECRSYPLYRFVREELGTAYLTGEKTRSPGEELNKVLLAINQGKHIDPLLECLKEWNGEPLPIN

>A2X7F7_Oryza_sativa_indica
MECENGRVSANGMSGLCVAAPRADPLNWGKATEEMTGSHLDEVKRMVAEYRQPLVKIEGASLRIAQVAAVAAAGEARVELDESARERVKASS
DWVMNSMMNGTDSYGVTTGFGATSHRRTKEGGALQRELIRFLNAGAFGTGTDGHVLPAEATRAAMLVRINTLLQGYSGIRFEILEAIAKLLN
ANVTPCLPLRGTITASGDLVPLSYIAGLVTGRENAVAVAPDGSKVNAAEAFKIAGIQGGFFELQPKEGLAMVNGTAVGSGLASTVLFEANIL
AILAEVLSAVFCEVMNGKPEYTDHLTHKLKHHPGQIEAAAIMEHILEGSSYMKHAKKLGELDPLMKPKQDRYALRTSPQWLGPQIEVIRAAT
KSIEREINSVNDNPLIDVSRGKALHGGNFQGTPIGVSMDNTRLAIAAIGKLMFAQFSELVNDFYNNGLPSNLSGGRNPSLDYGFKGAEIAMA
SYCSELQFLGNPVTNHVQSAEQHNQDVNSLGLISSRKTAEAIDILKLMSSTFLIALCQAVDLRHIEENVKSAVKSCVMTVAKKTLSTNSTGD
LHVARFCEKDLLKEIDREAVFAYADDPCSHNYPLMKKLRNVLVERALANGAAEFNADTSVFAKVAQFEEELRATLPGAIEAARAAVENGTAA
IPSRITECRSYPLYRFVREELGTKYLTGEKTRSPGEELNKVLVAINEGKHIDPLLECLKEWNGEPLPIC

>C0HJ40_Zea_mays
MECENGRGVAATNSDSLCMATPRADPLNWGKAAEELMGSHLDEVKRMVAEYRQPLVKIEGASLSIAQVAAVATGAGEARVELDESARSRVKA
SSDWVMTSMMNGTDSYGVTTGFGATSHRRTKEGGALQRELIRFLNAGAFGTGADGHVLPAETTRAAMLVRINTLLQGYSGIRFEILEAIVKL
LNANVTPCLPLRGTVTASGDLVPLSYIAGLVTGRENSVAVAPDGSKVNAAEAFKIAGIQGGFFELQPKEGLAMVNGTAVGSGLASTVLFEAN
ILAILAEVLSAVFCEVMNGKPEYTDHLTHKLKHHPGQIEAAAIMEHILEGSSYMKLAKKLGELDPLMKPKQDRYALRTSPQWLGPQIEVIRA
ATKSIEREINSVNDNPLIDVARSKALHGGNFQGTPIGVSMDNTRLAIAAIGKLMFAQFSELVNDYYNNGLPSNLSGGRNPSLDYGFKGAEIA
MASYCSELQFLGNPVTNHVQSAEQHNQDVNSLGLISSRKTAEAIEILKLMSSTFLIALCQAVDLRHIEENVKSAVKSCVMTVAKKTLSTNST
GGLHVARFCEKDLLQEIEREAVFAYADDPCSANYPLMKKLRNVLVERALANGAAEFNAETSVFAKVAQFEEDLRAALPKAVEAARAAVENGT
AGIPNRIAECRSYPLYRFVREELGAVYLTGEKTRSPGEELNKVLVAINQGKHIDPLLECLKEWNGEPLPIC

>A0A096TA22_Zea_mays
MACDSPCMATPRADPLNWGKAAEELMGSHLDEVKRMVAEYRQPLVKIEGASLSIAQVAAVATGVGEARVELDESARSRVKASSDWVMSSMMN
GTDSYGVTTGFGATSHRRTKEGGALQRELIRFLNAGAFGTGTDGHVLPAEATRAAMLVRINTLLQGYSGIRFEILEAIVKLLNANVTPCLPL
RGTVTASGDLVPLSYIAGLVTGRENSVAVAPDGSKVNAAEAFKIAGIQGGFFELQPKEGLAMVNGTAVGSGLASTVLFEANILAILAEVLSA
VFCEVMNGKPEYTDHLTHKLKHHPGQIEAAAIMEHILEGSSYMKLAKKLGELDPLMKPKQDRYALRTSPQWLGPQIEVIRAATKSIEREINS
VNDNPLIDVARSKALHGGNFQGTPIGVSMDNTRLAIAAIGKLMFAQFSELVNDYYNNGLPSNLSGGRNPSLDYGFKGAEIAMASYCSELQFL
GNSVTNHVQSAEQHNQDVNSLGLISSRKTAEAIEILKIMSSTFLIALCQAVDLRHIEENVKSAVKSCVMTVAKKTLSTDSTGGLHVARFCEK
DLLQEIEREAVFAYADDPCSANYPLMKKLRNVLVERALANGAAEFNAETSVFAKVAQFEEDLRAALPKAVEAARAAVENGTAAIPNRITDCR
SYPLYRFVREELGAVYLTGEKTRSPGEELNKVLVAINQGKHIDPLLECLKEWNGEPLPIC

>I1IBR8_Brachypodium_distachyon
MECENGQFAANGTGLCMATPRADPLNWGKAAEELTGSHLDEVKRMVAEYRKPVVTIEGATLSIAKVAAVAAAGEAKVELDESARERVKASSD
WVMNSMANGVDSYGVTTGFGATSHRRTKEGGALQRELIRFLNAGAFGTGSDGHVLPAGATRAAMLVRINTLLQGYSGIRFEILEAIAKLLNA
NVTPCLPLRGTITASGDLVPLSYIAGLVTGRENSVAVTPDGRKVNAAEAFKIAGIHGGFFELQPKEGLAMVNGTAVGSGLASTVLFEANILA
VLAEVISAVFCEVMNGKPEFTDHLTHKLKHHPGQIEAAAIMEHILEGSSYMKLAKKLGDLDPLMKPKQDRYALRTSPQWLGPQIEVIRAATK
SIEREINSVNDNPLIDVSRGKAIHGGNFQGTPIGVSMDNTRLALAAIGKLMFAQFSELVNDFYNNGLPSNLSGSRNPSLDYGFKGAEIAMAS
YCSELQFLGNPVTNHVQSAEQHNQDVNSLGLISSRKTAEAVEILKLMSSTFLVALCQAIDLRHIEENVKSAVKSCVMTVAKRTLSTNSTGGL
HVARFCEKDLLQEIEREAVFAYADDPCSANYPLMKKLRGVLVERALSNGKAEFNAETSVFAKVAQFEEELRTALPKAVEAARSAVESGTAAT
PNRIKECRSYPLYRFVREELGTAYLTGEKTRSPGEELNKVLVAINQGKHIDPLLECLKEWNGEPLPIC

>I1IBL7_Brachypodium_distachyon
MECENGQFAANGTGLCMATPSADPLNWGKAAEELTGSHLDEVKRMVAEYRKPVVTIEGATLSIAKVAAVAAAGEAKVELDESARERIKASSD
WVMNSMMNGTDSYGVTTGFGATSHRRTKEGGALQRELIRFLNAGAFGTGSDGHVLPAGATRAAMLVRINTLLQGYSGIRFEILEAIAKLLNA
NVTPCLPLRGTITASGDLVPLSYIAGLVTGRENSVAVTPDGRKVNAAEAFKLAGIHGGFFELQPKEGLAMVNGTAVGSGLASTVLFEANILA
VLAEVISAVFCEVMNGKPEFTDHLTHKLKHHPGQIEAAAIMEHILEGSSYMKLAKKLGDLDPLMKPKQDRYALRTSPQWLGPQIEVIRAATK
SIEREINSVNDNPLIDVSRGKAIHGGNFQGTPIGVSMDNTRLALAAIGKLMFAQFSELVNDFYNNGLPSNLSGSRNPSLDYGFKGAEIAMAS
YCSELQFLGNPVTNHVQSAEQHNQDVNSLGLISSRKTAEAVEILKLMSSTFLVALCQAIDLRHIEENVKIAVKSCVMTVAKKTLSTNSTGGL
HVARFCEKDLLQEIEREAVFAYADDPCSANYPLMKKLRSVLVERALSNGMAEFNAETSVFAKVAQFEEELRTVLPKAVEAARAAVESGTAAT
PNRIKECRSYPLYRFVREELGTAYLTGEKTRSPGEELNKVLVAINQGKHIDPLLECLKEWNGEPLPIC

>I1IBR7_Brachypodium_distachyon
MARENARVAAANGICTAIQHADPLNWGKAAEELTGSHLDEVKRMVVEYREPVVTIEGASLSIAKVAAVAAAGEAKVELDESARERVKASSDW
VMNSMANGVDSYGVTTGFGATSHRRTKEGGALQRELIRFLNAGAFGTGSDGHVLPAGATRAAMLVRINTLLQGYSGIRFEILEAIAKLLNAN
VTPCLPLRGTITASGDLVPLSYIAGLVTGRENSVAVAPDGRKVNAAEAFKIAGIHGGFFELQPKEGLAMVNGTAVGSGLASTVLFEANILAV
LAEVISAVFCEVMNGKPEFTDHLTHKLKHHPGQIEAAAIMEHILEGSSYMKLAKKQGELDPLMKPKQDRYALRTSPQWLGPQIEVIRAATKS
IEREINSVNDNPLIDVSRGKAIHGGNFQGTPIGVSMDNTRLALAAIGKLMFAQFSELVNDLYNNGLPSNLSGGRNPSLDYGLKGAEIAMASY
CSELQFLGNPVTNHVQSAEQHNQDVNSLGLISSRKTAEAIEILKLMSSTFLVALCQAIDLRHIEENVKSAVKSCVMTVAKKTLSTNSTGDLH

VSRFCEKDMLQEIDREAVFAYADDPCNHNYPLMKKLRGVLVESALANGVAEYNVETSVFAKVAQFEEELRAALPKAVEAARAAVESGTAATP
NRITECRSYPLYRFVREELGTVFLTGEKTRSPGEELNKVLMAINQGKHIDPLLECLKEWNGEPLPIC

>A0A0Q3JNS6_Brachypodium_distachyon
MTMASKNVHVSADGYLILCPATSQHADPLNWGKAAEALTGSHLEEVKRMVAEYRQPVVTIEGASLSIAKVAAVAAAGEAQVQLDESARERVK
ASSDWVMDSMANGVDSYGVTTGFGATSHRRTKEGGALQRELIRFLNAGAFGTGSDGHVLPAGATRAAMLVRINTLLQGYSGIRFEILEAIAK
LLNANVTPCLPLRGTITASGDLVPLSYIAGLVTGRENSVAVAPDGSKVNAAEAFKIAGIHGGFFELQPKEGLAMVNGTAVGSGLASTVLFDA
NVLAVMAEVISAVFCEVMNGKPEFTDHLTHKLKHHPGQIEAAAIMEHILEGSSYMKLAKKLGDLDPLMKPKQDRYALRTSPQWLGPQIEVIR
AATKSIEREINSVNDNPLIDVSRGKAIHGGNFQGTPIGVSMDNTRLALAAIGKLMFAQFSELVNDFYNNGLPSNLSGGRNPSLDYGFKGAEI
AMASYCSELQFLGNPVTNHVQSAEQHNQDVNSLGLISARKTAEAIEILKLMTSTFLVALCQAIDLRHIEENVKSAVTSCVRAVAKKTLSTNS
AGGLHVARFSEKDLIQEIDREAVFAYADDPCNPNYPLMKKLRGVLVERALANGVAEFDAETSVFAKVARFEEELRAALPVAVEAARAAVESG
TAEAPNRIAECRSYPLYRFVRQELGTVYLTGEKTRSPGEELNKVLVAINQGKHIDPLLECLKEWNGEPLPIC

>I1IAZ3_BRADI_Brachypodium_distachyon
MACENGQVAANGICTAIQHADPLNWGKAAEALTGSHLEEVKRMVAEYRQPVVTIEGASLSIAKVAAVAAAGEAQVQLDESARERVKASSDWV
MDSMANGVDSYGVTTGFGATSHRRTKEGGALQRELIRFLNAGAFGTGSDGHVLPAGATRAAMLVRINTLLQGYSGIRFEILEAIAKLLNANV
TPCLPLRGTITASGDLVPLSYIAGLVTGRENSVAVAPDGSKVNAAEAFKIAGIHGGFFELQPKEGLAMVNGTAVGSGLASTVLFDANVLAVM
AEVISAVFCEVMNGKPEFTDHLTHKLKHHPGQIEAAAIMEHILEGSSYMKLAKKLGDLDPLMKPKQDRYALRTSPQWLGPQIEVIRAATKSI
EREINSVNDNPLIDVSRGKAIHGGNFQGTPIGVSMDNTRLALAAIGKLMFAQFSELVNDFYNNGLPSNLSGGRNPSLDYGFKGAEIAMASYC
SELQFLGNPVTNHVQSAEQHNQDVNSLGLISARKTAEAIEILKLMTSTFLVALCQAIDLRHIEENVKSAVTSCVRAVAKKTLSTNSAGGLHV
ARFSEKDLIQEIDREAVFAYADDPCNPNYPLMKKLRGVLVERALANGVAEFDAETSVFAKVARFEEELRAALPVAVEAARAAVESGTAEAPN
RIAECRSYPLYRFVRQELGTVYLTGEKTRSPGEELNKVLVAINQGKHIDPLLECLKEWNGEPLPIC

>A0A059Q1B2_Saccharum_hybrid_cultivar_R570
MASNTAILESDPLNWGKAAAELTGSHLDEVKRMVAQFRDPVVKIEGSTLRVGQVAAVAAAKDASGVAVELDEEARPRVKASSEWILDCIAHG
GDIYGVTTGFGGTSHRRTKDGPALQVELLRHLNAGIFGNGSDGHTLPSEVSRAAMLVRINTLLQGYSGIRFEILEAITKLLNTGVSPCLPLR
GTITASGDLVPLSYIAGLITGRPNAQAVTVDGRKVDAAEAFKVAGIEGGFFKLNPKEGLAIVNGTSVGSALAAMVCFDANVLAVLSSVLSAV
FCEVMNGKPEYTDHLTHKLKHHPGSIESAAIMEHILDGSSFMKHAKEVNAMDPLLKPKQDRYALRTSPQWLGPQIEVIRAATKSIEREVNSV
NDNPVIDVHRGKALHGGNFQGTPIGVSMDNARLAIANIGKLMFAQFSELVNEFYNNGLTSNLAGSRNPSLDYGFKGTEIAMASYCSELQYLA
NPITNHVQSAEQHNQDVNSLGLVSARKTAEAVDILKLMSSTYMVALCQAVDLRHLEENLKSAVKNSVMAVARKVLTTSLDGDLHSARFSEKA
LLTAIDREAVYGYYDDPCSANSPLMKKIRAVLVDHALANGEAEKDASASVFSKINRFEETLREVLPREMEAARVAFETGTAPIANRIKESRS
YPLYRFIRQDLGAVYLTGEKLKSPGEECNKVFLALSEGKLIDPMLECLKEWDGKPLPIC

>U3M000_Saccharum_hybrid_cultivar_Co_93009
GKPEYTDHLTHKLKHHPGSIEAAAIMEHILDGSAFMKHAKKVNELDPLLKPKQDRYALRTSPQWLGPQIEVIRAATKSIEREVNSVNDNPVI
DVHRGKALHGGNFQGTPIGVSMDNARLAIANIGKLMFAQFSELVNEFYNNGLTSNLAGSRNPSLDYGFKGTEIAMASYCSELQYLGNPITNH
VQSAEQHNQDVNSLGLVSARKTAEAIDILKLMSSTYIVALCQAIDLR

>M4XZQ1_Saccharum_hybrid_cultivar_CP69-1062
MAGNGAIVESDPLNWGAAAAELAGSHLDEVKRMVAQARQPVVKIEGSTLRVGQVAAVAAAKDASGVAVELDEEARPRVKASSEWILDCIAHG
GDIYGVTTGFGGTSHRRTKDGPALQVELLRHLNAGIFGTGSDGHTLPSEVVRAAMLVRINTLLQRYSGIPFEILEAITKLLNTGVSPCLPLR
GTITASRDLVPLSYIAGLITGRPNAQATTVDGRKVDAAEAFKIAGIEGGFFKLNPKEGLAIVNGSSVGSALAATVMYDAYVLTVLSEVLSAV
LCEVMNRMPEYTDHLTHKLKHHPGSIEAAAIMEHILDGSAFMKHAKKVNELDPLIKPKQDRYALRTSPQWLGPQIEVIRAATKSIEREVNSV
NDNPVMDVHRGKALHGGNFQGTPIGVSMDNARLAIANIGKLMFAQFSELVNEFYNNGLTSNLAGSRNPSLDYGLKGTENAMASYCSELQYLG
NPITNHVQSAEQLNQDVNSFGLVSARKTAEAIDILKLMSSTYIVALCQAIDLRHLEENIKTSVKNRVTQVAKKVLTMNPSGDLSSARFSEKE
LITAIDREGVFTYSEDPASGSLPLMQKLRSVLVDHALSSGDAGTGPLRVLQDHQIRGGAPRGAGPGGGRRPASPWAEGTAPGRNRNWDSRSF
PLYRFVREELGCVFVTGEKLKSPGEECSKVFNGISQGKLVDPKLECLKEWDGKPLPINVVN

>W5RSK7_Saccharum_hybrid_cultivar_HSF-240
MAGNGAIVESDPLNWGAAAAELAGSHLDEVKRMVAQARQPVVKIEGSTLRVGQVAAVAAAKDASGVAVELDEEARPRVKASSEWILDCIAHG
GDIYGVTTGFGGTSHRRTKDGPALQVELLRHLNAGIFGTGTDGHTLPSEVSRAAMLVRINTLLQGYSGIRFEILEDITKLLNTGVSPCLPLR
GTITASGDLVPLSYIAGLITGRPNAQAVTVDGRKVDAAEAFKVAGIEGGFFKLNPKEGLAIVNGTSVGSPLAAMVCFDANVLAVLSSVLSAV
FCEVMNGKPEYTDHLTHKLKHHPGSIESAAIMEHILDGSSFMKHAKEVNAMDPLLKPKQDRYALRTSPQWLGPQIEVIRAATKSIEREVNSV
NDNPVIDVHRGKALHGGNFQGTPIGVSMDNARLAIANIGKLMFAQFSELVNEFYNNGLTSNLAGSRNPSLDYGFKGTEIAMASYCSELQYLA
NPITNHVQSAEQHNQDVNSLGLVSARKTAEAVDILKLMSSTYMVALCQAVDLRHLEENLKTSVKNTVTQVAKKVLTMNPSGELSSARFSEKE
LISAIEREDVCTHAEDPASVSLPLMQKLRTVLVDHALSSSDAGTGALRVLQDHHVRGGAPRGAAPGGGRRPALPWAEGTAPGRNRSWDSRSF
PLYRFVREELGCVFLTGEKLKSPGEECTKVFPGISQGKLVDPMLECLKEWDGKPLPIN

>A2IBN5_Saccharum_officinarum
MAGNGAIVESDPLNWGAAAAELAGSHLDEVKRMVAQARQPVVKIEGSTLRVGQVAAVAAAKDASGVAVELDEEARPRVKASSEWILDCIAHG
GDIYGVTTGFGGTSHRRTKDGPALQVELLRHLNAGIFGTGSDGHTLPSEVVRAAMLVRINTLLQGYSGIRFEILEAITKLLNTGVSPCLPLR
GTITASGDLVPLSYIAGLITGRPNAQATTVDGRKVDAAEAFKIAGIEGGFFKLNPKEGLAIVNGTSVGSALAATVMYDANVLTVLSEVLSAV
FCEVMNGKPEYTDHLTHKLKHHPGSIEAAAIMEHILDGSAFMKHAKKVNELDPLLKPKQDRYALRTSPQWLGPQIEVIRAATKSIEREVNSV
NDNPVIDVHRGKALHGGNFQGTPIGVSMDNARLAIANIGKLMFAQFSELVNEFYNNGLTSNLAGSRNPSLDYGFKGTEIAMASYCSELQYLG
NPITNHVQSAEQHNQDVNSLGLVSARKTAEAIDILKLMSSTYIVALCQAIDLRHLEENIKTSVKNTVTQVAKKVLTMNPSGDLSSARFSEKE
LITAIDREGVFTYAEDPASGSLPLMQKLRSVLVDHALSSGDAGTGALRVLQDHQFRGGAPRGAGPGGGRRPASPWAEGTAPGRNRNWDSRSF
PLYRFVREELGCVFLTGEKLKSPGEECTKVFNGISQGKLVDPMLECLKEWDGKPLPINVVN

>W5RSQ2_Saccharum_hybrid_cultivar_Co1148
MAGNGAIVESDPLNWGAAAAELAGSHLDEVKRMVAQARQPVVKIEGSTLRVGQVAAVAAAKDASGVAVELDEEARPRVKASSEWILDCIAHG
GDIYGVTTGFGGTSHRRTKDGPALQVELLRHLNAGIFGTGSDGHTLPSEVVRAATLVRINTLLQGYSGIRFEILEAITKLLNTGVSPCLPLR

GTITASGDLVPLSYVAGLITGRPNAQATTIGGRKVDAAGAFKIAGIEGGFFKLNPKEGLAIVNGTSVGSALAATVMYDANVLAVLSEVLSAV
FCEVMNGKPEYTDHLTHKLKHHPGSIEAAAIMEHILDGSSFMKHAKEVNAMDPLLKPKQDRYALRTSPQWLGPQIEVIRAATKSIEREVNSV
NDNPVIDVHRGKALHGGNFQGTPIGVSMDNARLAIANIGKLMFAQFSELVNEFYNNGLTSNLAGSRNPSLDYGFKGTEIAMASYCSELQYLA
NPITNHVLGAEQQDQDVNSLGLVSARKTAEAVDILKLMSSTYMVALCQAVDLRHLEENLKTSVKNTVTQVAKKVLTMNPSGDLSSARFSEKE
LISAIDREAVFTYAEDPASGSLPLMQKLRPVLVDHALSSGDAGTGALRVLQDHQVRGGAPRGAGPGGGRRPALPWAEGTAPGANRTWDSRSF
PLYRFVREELGCVFLTGEKLKSPGEECNKVFPGISQGKLVDPMLECLKEWDGKPLPINVVNC

>M1MQ13_Saccharum hybrid cultivar ROC22 PE=2 SV=1
MAGNGAIVESDPLNWGAAAAELAGSHLDEVKRMVAQARQPVVKIEGSTLRVGQVAAVAAAKDASGVAVELDEEARPRVKASSEWILDCIAHG
GDIYGVTTGFGGTSHRRTKDGPALQVELLRHLNAGIFGTGSDGHTLPSEVVRAAMLVRINTLLQGYSGIRFEILEAITKLLNTGVSPCLPLR
GAITASGDLVPLSYIAGLITGRPNAQATTIDGRKVDAAEAFKIAGIEGGFFKLNPKEGLAIVNGTSVGSALAATVMYDANVLAILSEVLSAV
FCEVMNGKPEYTDHLTHKLKHHPGSIEAAAIMEHILDGSAFMKHAKKVNELDPLLKPKQDRYALRTSPQWLGPQIEVIRAATKSIEREVNSV
NDNPVIDVHRGKALHGGNFQGTPIGVSMDNARLAIANIGKLMFAQFSELVNEFYNNGLTSNLAGSRNPSLDYGFKGTEIAMASYCSELQYLG
NPITNHVQSAEQHNQDVNSLGLVSARKTAEAIDILKLMSSTYIVALCQAIDLRHLEENIKTSVKNTVTQVAKKVLTMNPSGDLSSARFSEKE
LITAIDREGVFTYAEDPASGSLPLMQKLRSVLVDHALSSGDAEREPSVFSKITRFEEELRAVLPREVEAARVAVAEGTAPVANRIADSRSFP
LYRFVREELGCVFLTGEKLKSPGEECTKVFNGISQGKLVDPMLECLKEWDGKPLPINIVN

>C5XXU0_Sorghum_bicolor
MACENGRVAATNGDGLCMATPRADPLNWGKAAEELMGSHLDEVKRMVAEYRQPLVKIEGASLRIAQVAAVAAGAGEARVELDESARGRVKAS
SDWVMNSMMNGTDSYGVTTGFGATSHRRTKEGGALQRELIRFLNAGAFGTGTDGHVLPAEATRAAMLVRINTLLQGYSGIRFEILEAIVKLL
NANVTPCLPLRGTVTASGDLVPLSYIAGLVTGRENSVAVAPDGTKVNAAEAFKIAGIQGGFFELQPKEGLAMVNGTAVGSGLASTVLFEANV
LAILAEVLSAVFCEVMNGKPEYTDHLTHKLKHHPGQIEAAAIMEHILEGSSYMKLAKKLGELDPLMKPKQDRYALRTSPQWLGPQIEVIRAA
TKSIEREINSVNDNPLIDVARSKALHGGNFQGTPIGVSMDNTRLAIAAIGKLMFAQFSELVNDYYNNGLPSNLSGGRNPSLDYGFKGAEIAM
ASYCSELQFLGNPVTNHVQSAEQHNQDVNSLGLISSRKTAEAIEILKLMSSTFLIALCQAVDLRHIEENVKSAVKSCVMTVAKKTLSTNSTG
GLHVARFCEKDLLQEIEREAVFAYADDPCSANYPLMKKLRNVLVERALANGAAEFDAETSVFAKVAQFEEELRAALPKAVEAARAAVENGTA
AIPNRITECRSYPLYRFVRQEVGAVYLTGEKTRSPGEELNKVLVAINQGKHIDPLLECLKEWNGEPLPIC

>C5XXT9_Sorghum_bicolor
MECETGLVRSLNGDGLCMSAQAAPRGADPLNWGKAAEDLSGSHLEEVKRMVAEFRDPVVKIQGASLSIAQVAAVAAGAGEARVELDESARER
VKASSDWVMSSMMNGTDSYGVTTGFGATSHRRTKEGGALQRELIRFLNAGAFGTGADGHVLPAEATRAAMLVRINTLLQGYSGIRFEILEAI
AKLLNANVTPCLPLRGTITASGDLVPLSYIAGLITGRQNSVAVAPDGRKVDAAEAFKIAGIEHGFFELQPKEGLAMVNGTAVGSGLASTVLF
EANVLAIMAEVISAVFCEVMTGKPEFTDHLTHKLKHHPGQIEAAAIMEHILEGSSYMKLAKKLGELDPLMKPKQDRYALRTSPQWLGPQIEV
IRFATKSIEREINSVNDNPLIDVSRGKALHGGNFQGTPIGVSMDNTRLALAAIGKLMFAQFSELVNDYYNNGLPSNLSGGRNPSLDYGFKGA
EIAMASYCSELQFLGNPVTNHVQSAEQHNQDVNSLGLISSRKTAEAIDILKLMTSTFLIALCQAIDLRHIEENVKAAVKNCVTQVAKKSLSL
NARGGLHNARFCEKDLQTAIDREAVFAYADDPCSPNYPLMQKLRAVLIEHALANGDAERVVETSIFAKVAEFEQQVRAALPKEVEAARAAVE
SGNPLVPNRIKECRSYPLYRFVREEVGTQYLTGEKTRSPGEELNKVLVAINQRKHVDPLLECLKEWNGEPLPLC

>C5XXT8_Sorghum_bicolor
MAGNGAIVESDPLNWGAAAAELSGSHLDEVKRMVAQARQPVVKIEGSTLRVGQVAAVASAKDASGVAVELDEEARPRVKASSEWILDCIAHG
GDIYGVTTGFGGTSHRRTKDGPALQVELLRHLNAGIFGTGSDGHTLPSEVVRAAMLVRINTLLQGYSGIRFEILEAITKLLNTGVSPCLPLR
GTITASGDLVPLSYIAGLITGRPNAQATTVDGRKVDAAEAFKIAGIEGGFFKLNPKEGLAIVNGTSVGSALAATVMYDANVLAVLSEVLSAI
FCEVMNGKPEYTDHLTHKLKHHPGSIEAAAIMEHILDGSAFMKHAKKVNELDPLLKPKQDRYALRTSPQWLGPQIEVLRAATKSIEREVNSV
NDNPVIDVHRGKALHGGNFQGTPIGVSMDNARLAIANIGKLMFAQFSELVNEFYNNGLTSNLAGSRNPSLDYGFKGTEIAMASYCSELQYLG
NPITNHVQSAEQHNQDVNSLGLVSARKTAEAIDILKLMSSTYIVALCQAIDLRHLEENIKTSVKNTVTQVAKKVLTMNPSGDLSSARFSEKE
LITAIDREGVFTYAEDPASASLPLMTKLRAVLVDHALSSGDAEREPSVFSKITKFEEELRAVLPREVEAARVAVAEGTAPVANRIADSRSFP
LYRFVREELGCVFLTGEKLKSPGEECTKVFNGINQGKLVDPMLECLKEWDGKPLPINVVN

>C5YCD6_Sorghum_bicolor
MASNTAILESDPLSWGKAAAELTGSHLDEVKRMVAQFRDPVVKIEGSTLRVGQVAAVAAAKDASGVAVELDEEARPRVKASSEWILDCIAHG
GDIYGVTTGFGGTSHRRTKDGPALQVELLRHLNAGIFGNGSDGHTLPSEVSRAAMLVRINTLLQGYSGIRFEILEAITKLLNTGVSPCLPLR
GTITASGDLVPLSYIAGLITGRPNAQAVTVDGRKVDAAEAFKVAGIQGGFFKLNPKEGLAIVNGTSVGSALAAMVCFDANVLAVLSSVLSAV
FCEVMNGKPEYTDHLTHKLKHHPGSIESAAIMEHILDGSSFMKHAKEVNAMDPLLKPKQDRYALRTSPQWLGPQIEVIRAATKSIEREVNSV
NDNPVIDVHRGKALHGGNFQGTPIGVSMDNARLAIANIGKLMFAQFSELVNEFYNNGLTSNLAGSRNPSLDYGFKGTEIAMASYCSELQYLA
NPITNHVQSAEQHNQDVNSLGLVSARKTAEAVDILKLMSSTYMVALCQAVDLRHLEENLKSAVKNCVMAAARKVLTTSLDGDLHSARFSEKA
LLTAIDREAVYGYYDDPCSANSPLMKKIRAVLVDHALASGEAEKDASASVFSKINRFEEALREALPREMEAARVAFETGTAPIGNRIKDSRS
YPLYRFIRQDLGAVYLTGEKLKSPGEECNKVFLALSEGKLIDPMLECLKEWDGKPLPIC

>C5XXU3_Sorghum_bicolor
MACDNGRVAATNGDGLCMATPRADPLNWGKAAEELMGSHLDEVKRMVAEYRQPLVKIEGASLRIAQVAAVAAGAGEARVELDESARGRVKAS
SDWVMNSMMNGTDSYGVTTGFGATSHRRTKEGGALQRELIRFLNAGAFGTGTDGHVLPAEATRAAMLVRINTLLQGYSGIRFEILEAIVKLL
NANVTPCLPLRGTVTASGDLVPLSYIAGLVTGRENSVAVAPDGTKVNAAEAFKIAGIQGGFFELQPKEGLAMVNGTAVGSGLASTVLFEANV
LAILAEVLSAVFCEVMNGKPEYTDHLTHKLKHHPGQIEAAAIMEHILEGSSYMKLAKKLGELDPLMKPKQDRYALRTSPQWLGPQIEVIRAA
TKSIEREINSVNDNPLIDVARSKALHGGNFQGTPIGVSMDNTRLAIAAIGKLMFAQFSELVNDYYNNGLPSNLSGGRNPSLDYGFKGAEIAM
ASYCSELQFLGNPVTNHVQSAEQHNQDVNSLGLISSRKTAEAIEILKLMSSTFLIALCQAVDLRHIEENVKSAVKSCVMTVAKKTLSTNSTG
GLHVARFCEKDLLQEIEREAVFAYADDPCSANYPLMKKLRNVLVERALANGAAEFDAETSVFAKVAQFEEELRAALPKAVEAARAAVENGTA
AIPNRITECRSYPLYRFVRQEVGAVYLTGEKTRSPGEELNKVLVAINQGKHIDPLLECLKEWNGEPLPIC
