# Supplementary material for: Metabolome Dynamics of Smutted Sugarcane Reveals Mechanisms Involved in Disease Progression and Whip Emission
Source: Front Plant Sci. 2017 May 31;8:882. doi: 10.3389/fpls.2017.00882 (PMC5450380; doi:10.3389/fpls.2017.00882)
Supplement: Supplementary file 4 [file Presentation2.PDF]

Supporting Information File S2. Fragmentation patterns of selected metabolites identified as responsive to smut pathogen in LC-ESI-MS/MS positive ionization mode. Metabolites were using ACD/Labs software to theoretical fragmentation of structures from Metlin database (https://metlin.scripps.edu/index.php).

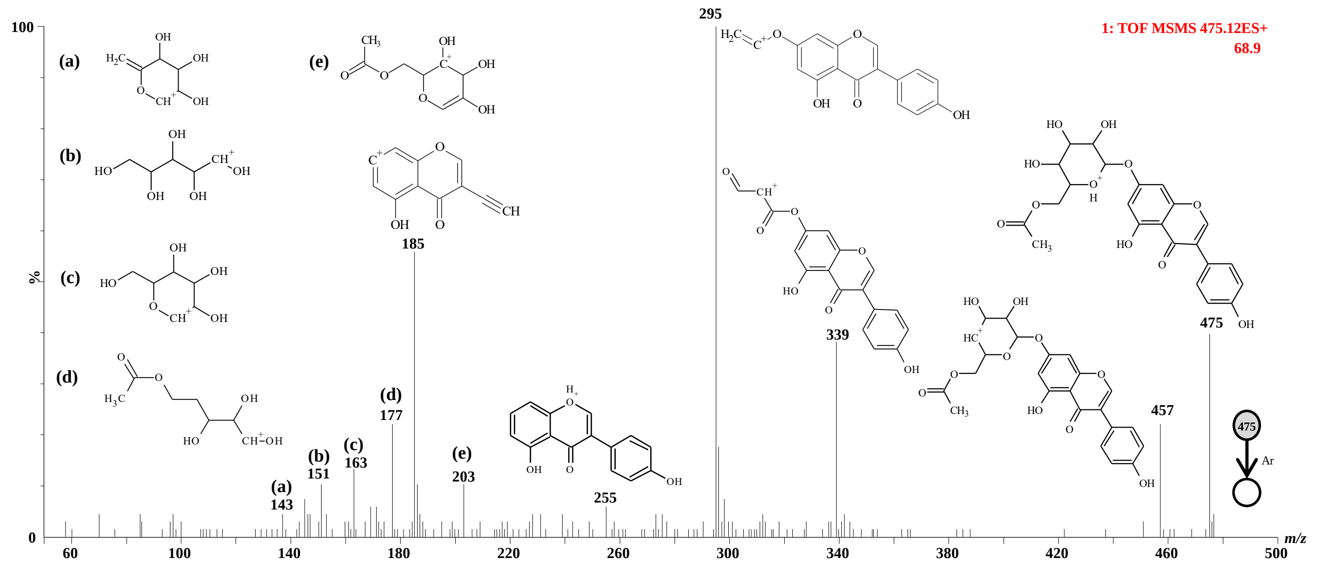

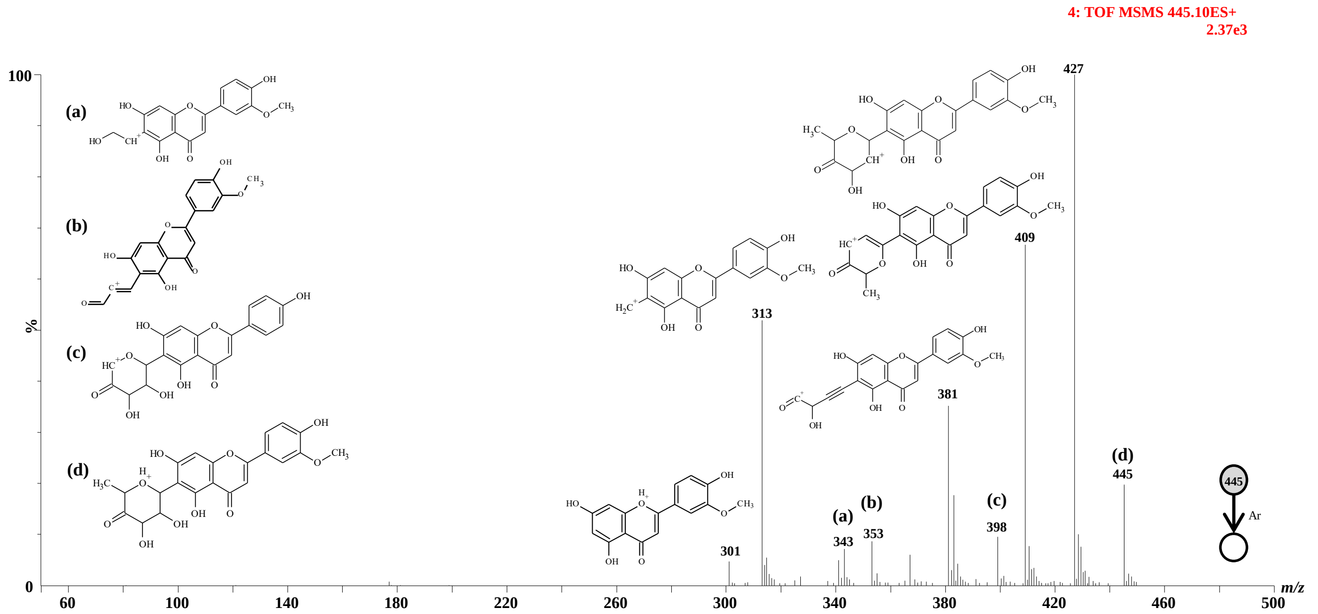

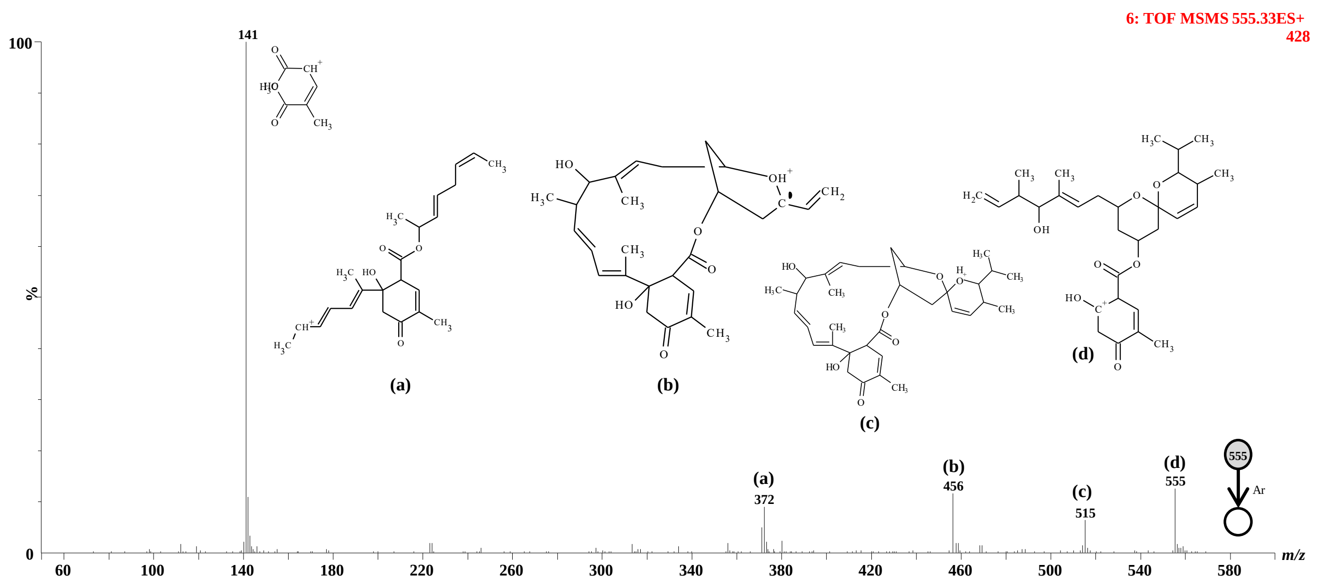

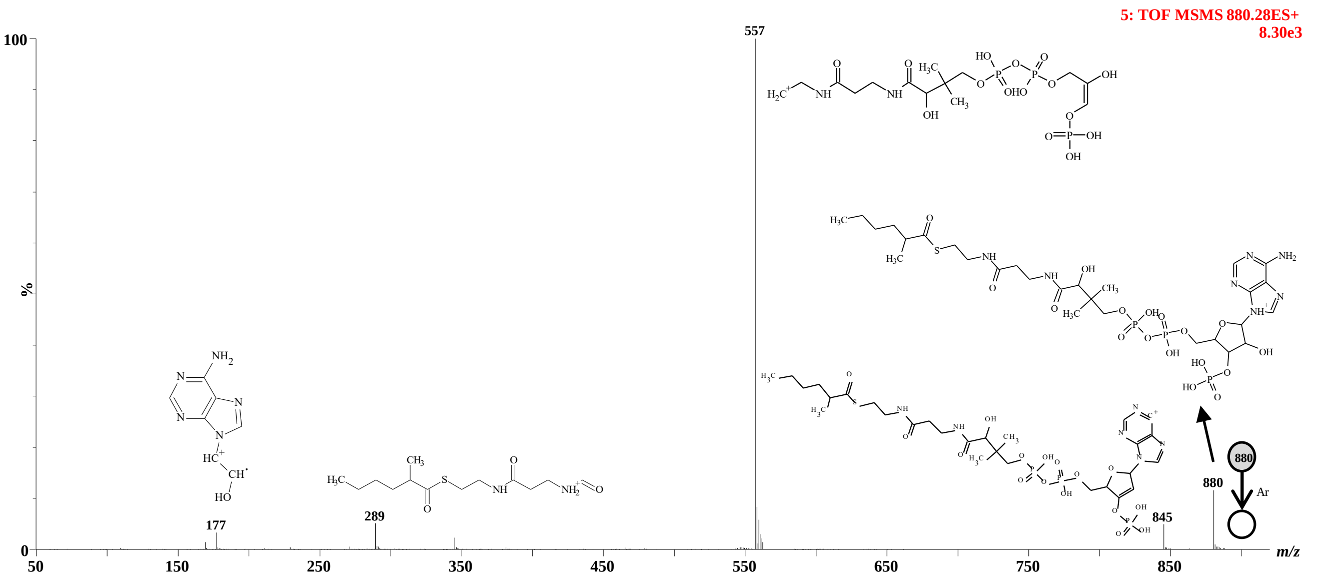

5: TOF MSMS 880.28ES+
8.30e3
100
%
0
557
177
289
845
880
m/z
50
150
250
350
450
550
650
750
850
Ar

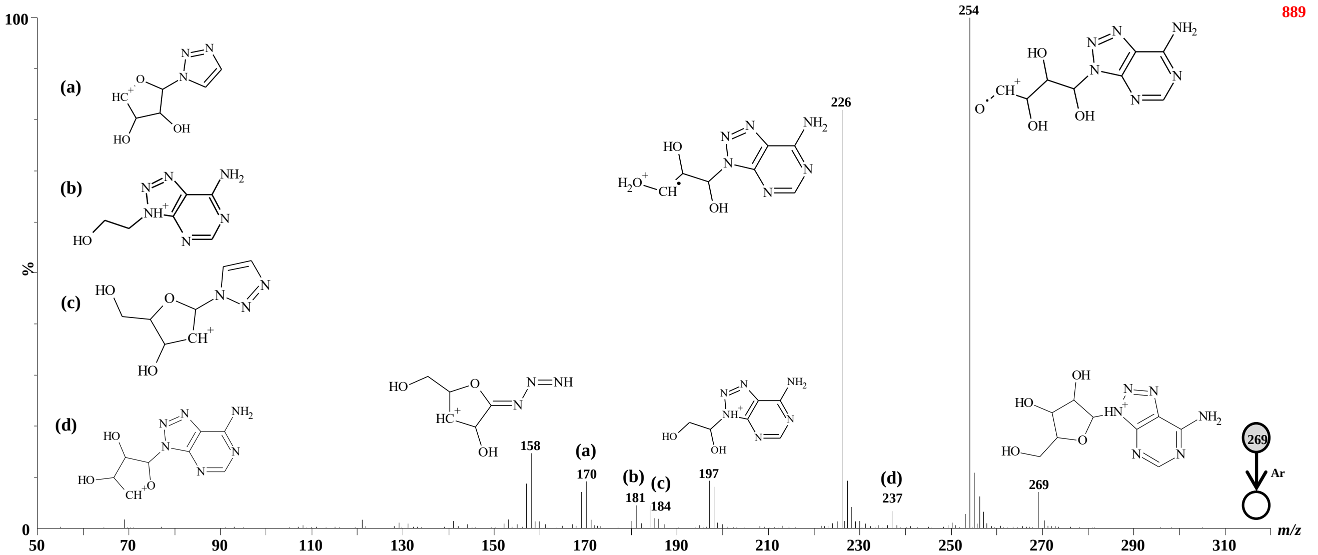

2: TOF MSMS 269.07ES+
889
100
%
0
254
226
158
(a) 170
(b) 181
(c) 184
197
(d) 237
269
(a)
(b)
(c)
(d)
m/z
50
70
90
110
130
150
170
190
210
230
250
270
290
310
Ar

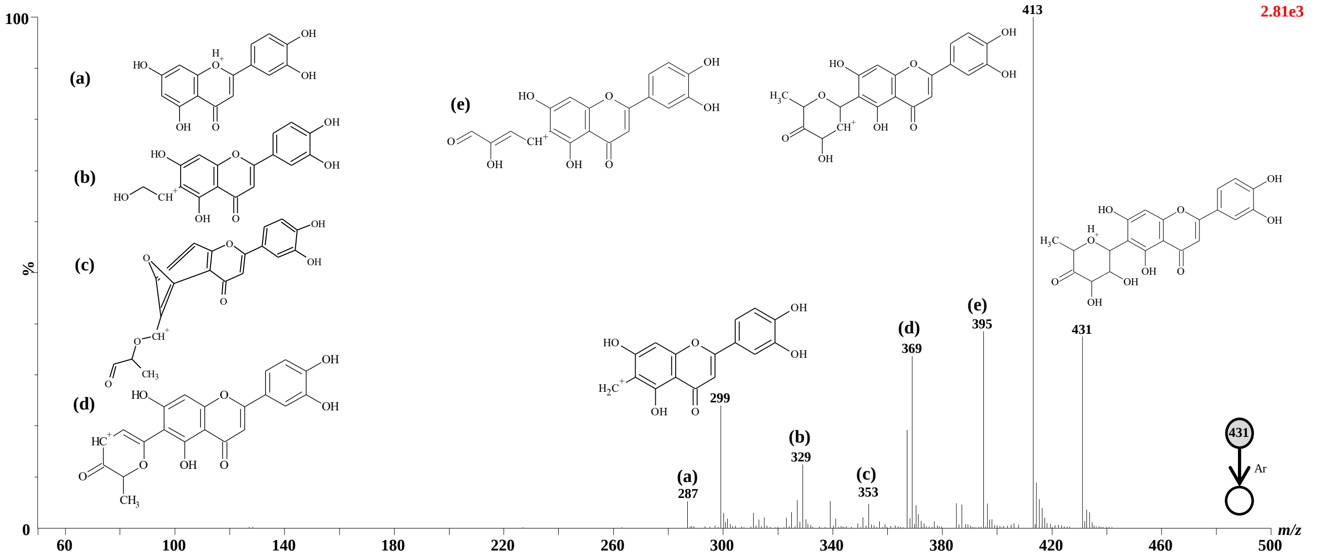

3: TOF MSMS 431.10ES+
2.81e3
100
%
0
413
(e) 395
431
(d) 369
299
(b) 329
(a) 287
(c) 353
(a)
(b)
(c)
(d)
(e)
m/z
60
100
140
180
220
260
300
340
380
420
460
500
Ar

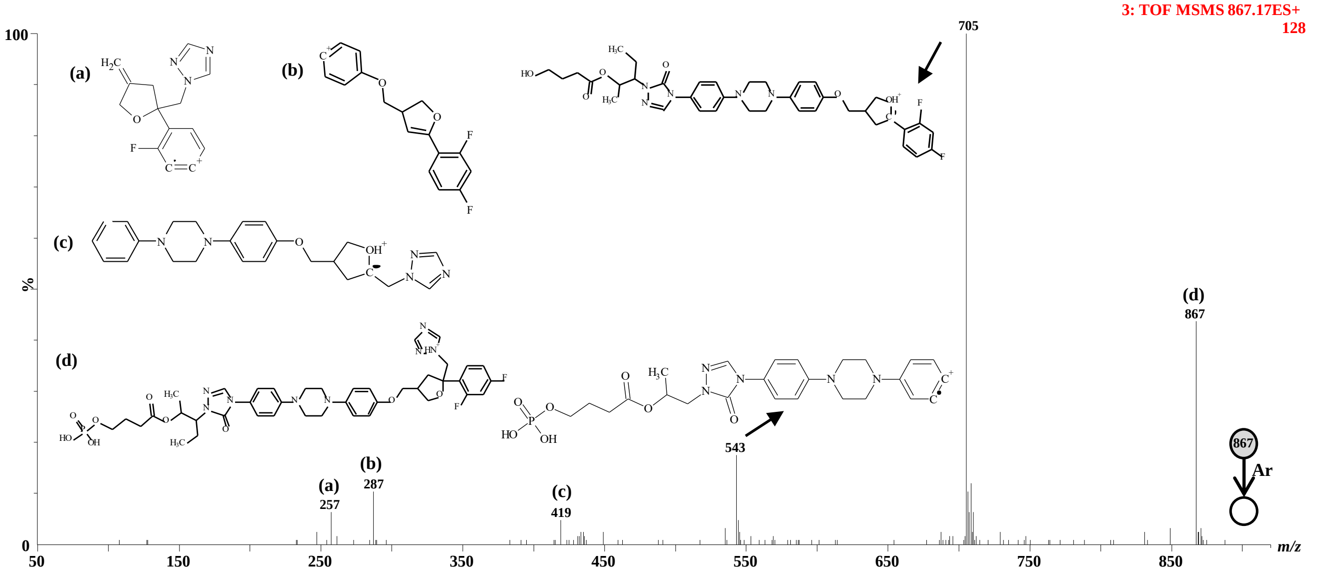
3: TOF MSMS 867.17ES+
128
100
%
0
(a)
(b)
(c)
(d)
(a) 257
(b) 287
(c) 419
543
705
(d) 867
867
Ar
m/z
50
150
250
350
450
550
650
750
850

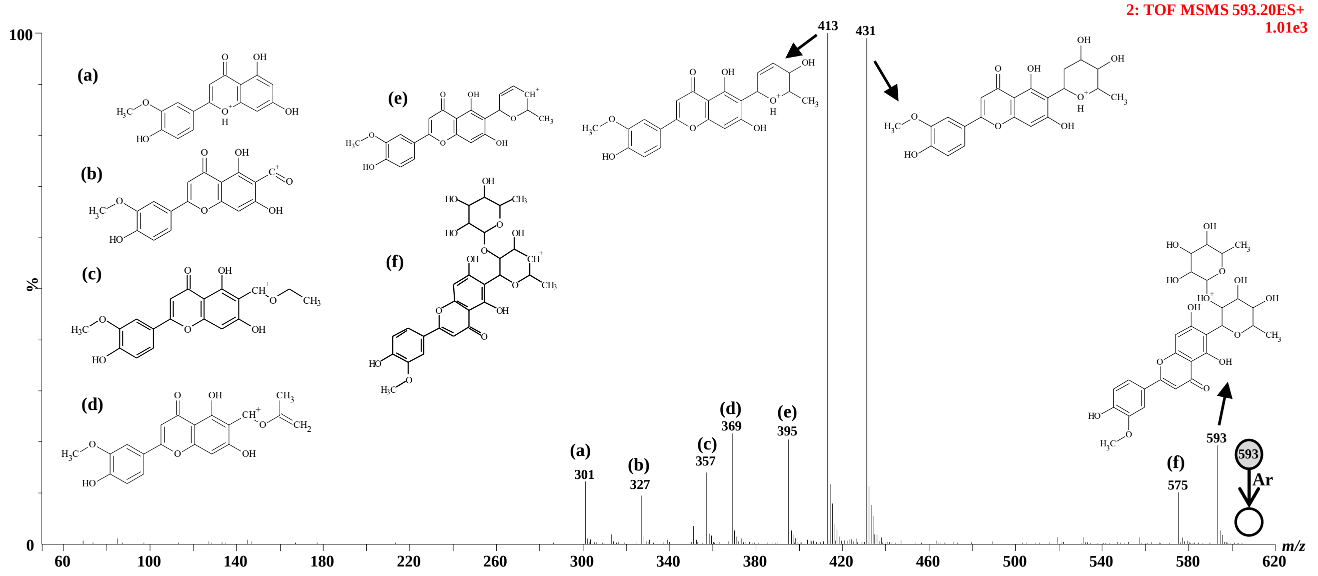
2: TOF MSMS 593.20ES+
1.01e3
100
%
0
(a)
(b)
(c)
(d)
(e)
(f)
(a) 301
(b) 327
(c) 357
(d) 369
(e) 395
413
431
(f) 575
593
593
Ar
m/z
60
100
140
180
220
260
300
340
380
420
460
500
540
580
620

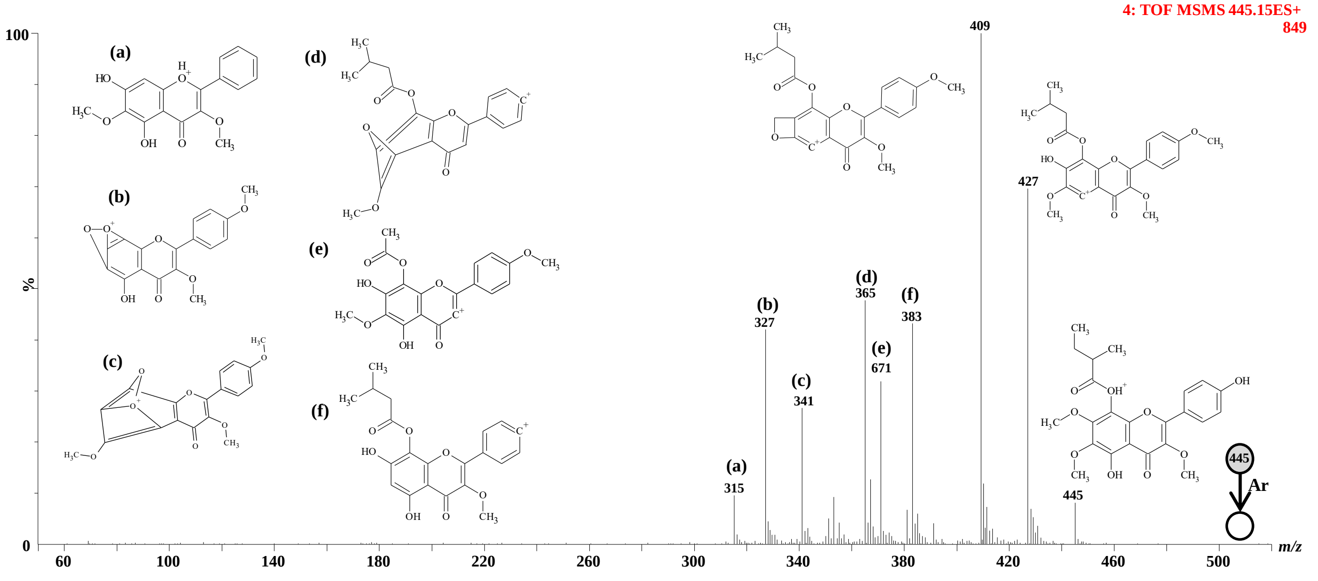
4: TOF MSMS 445.15ES+
849
100
%
0
(a)
(b)
(c)
(d)
(e)
(f)
(a) 315
(b) 327
(c) 341
(d) 365
(e) 671
(f) 383
409
427
445
445
Ar
m/z
60
100
140
180
220
260
300
340
380
420
460
500

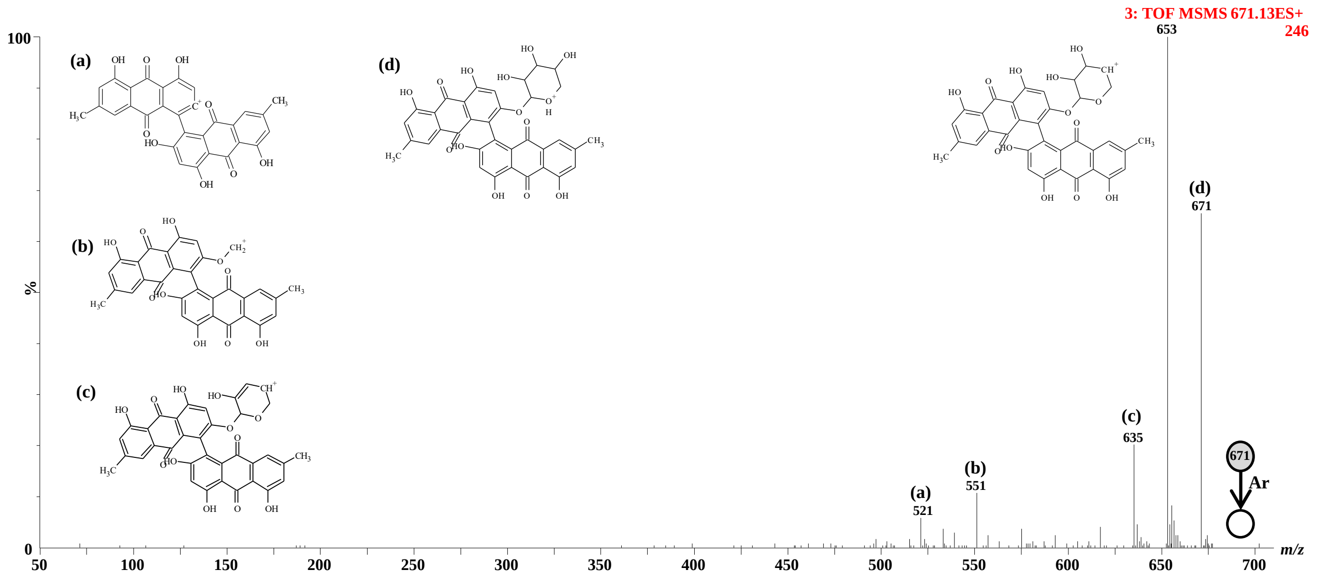
3: TOF MSMS 671.13ES+
246
653
(d)
671
(c)
635
(b)
551
(a)
521
671
Ar
m/z
%

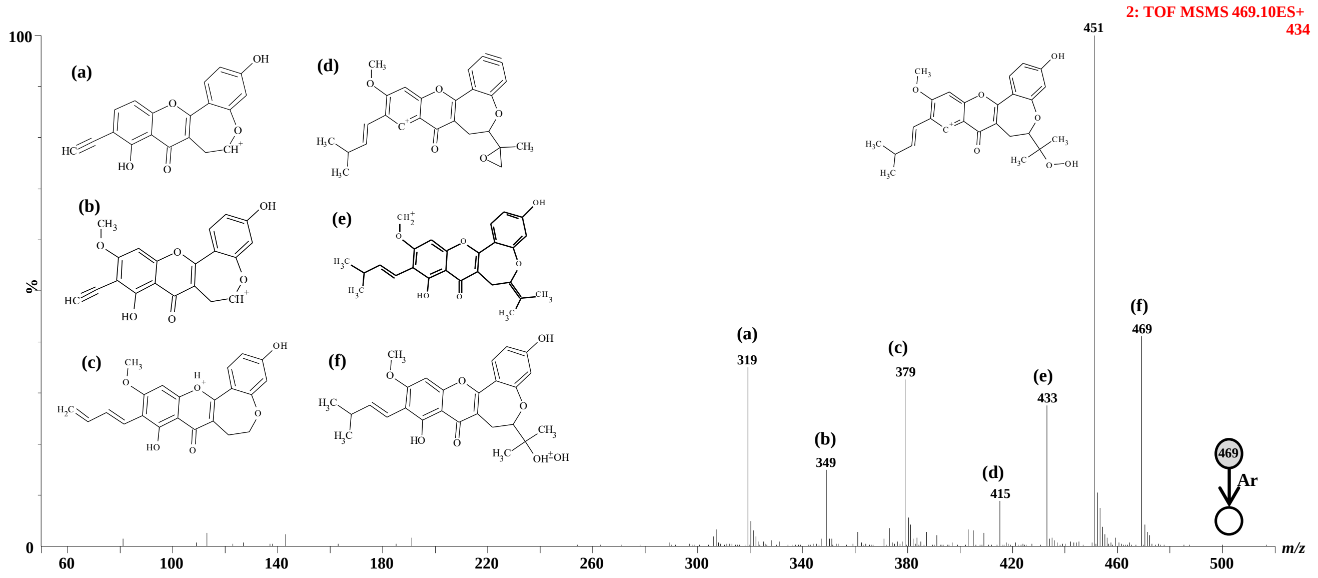
2: TOF MSMS 469.10ES+
434
451
(a)
319
(b)
349
(c)
379
(d)
415
(e)
433
(f)
469
469
Ar
m/z
%

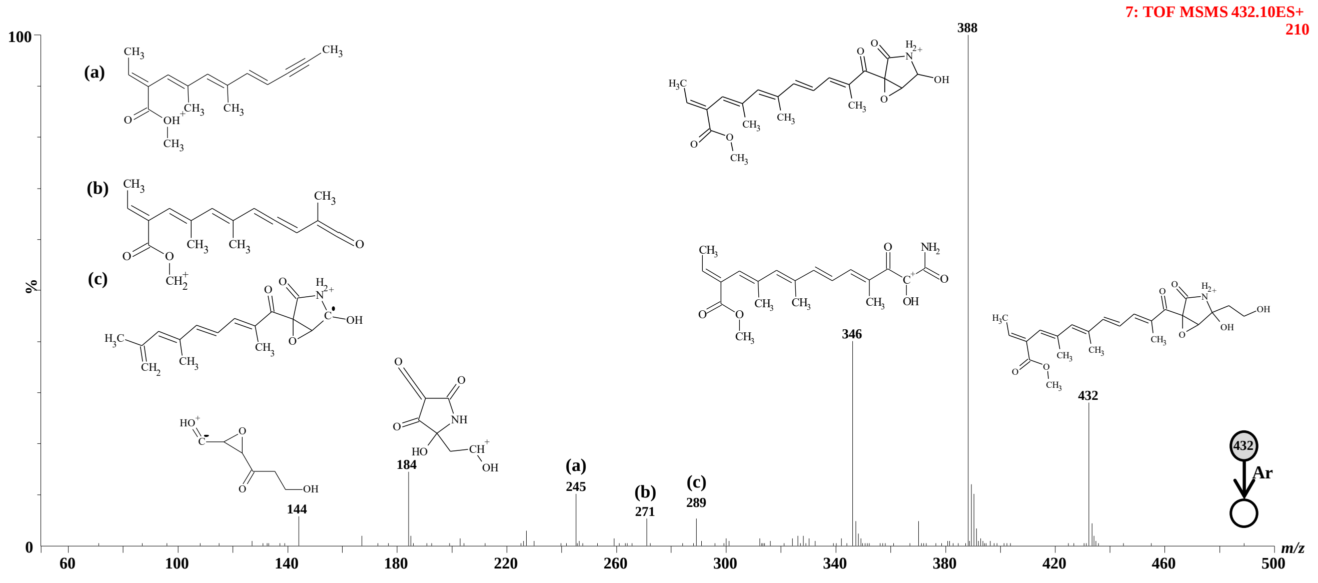
7: TOF MSMS 432.10ES+
210
388
346
432
144
184
(a)
245
(b)
271
(c)
289
432
Ar
m/z
%

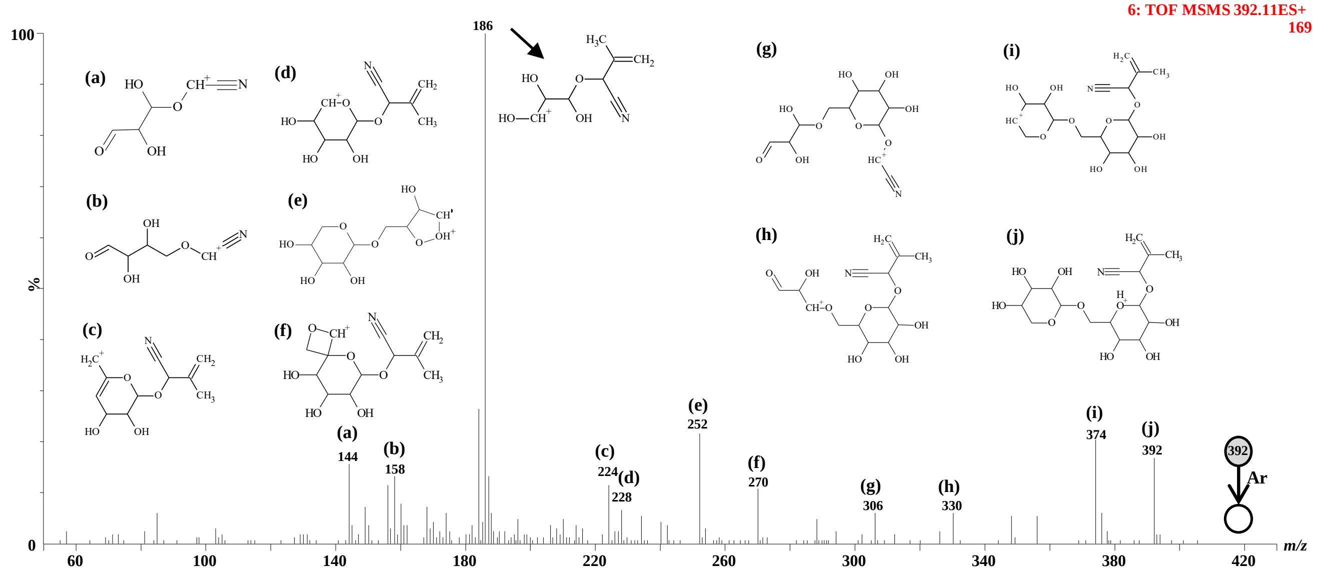
6: TOF MSMS 392.11ES+
169
100
%
0
186
(a) 144
(b) 158
(c) 224
(d) 228
(e) 252
(f) 270
(g) 306
(h) 330
(i) 374
(j) 392
392
Ar
m/z
60
100
140
180
220
260
300
340
380
420

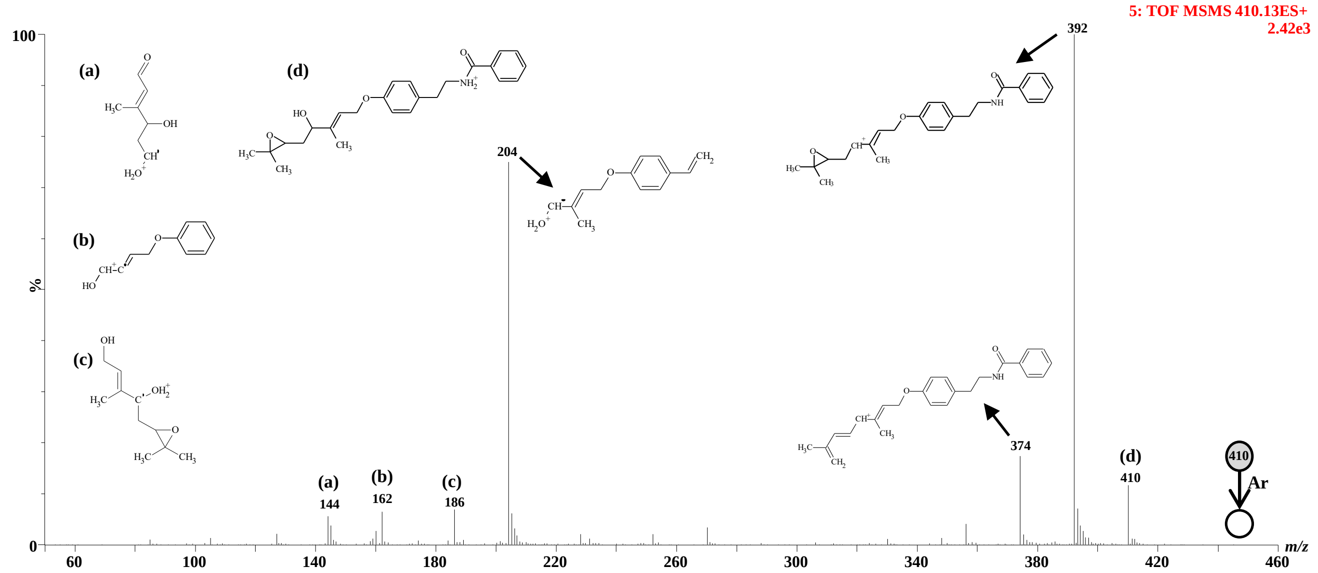
5: TOF MSMS 410.13ES+
2.42e3
100
%
0
204
392
374
(a) 144
(b) 162
(c) 186
(d) 410
410
Ar
m/z
60
100
140
180
220
260
300
340
380
420
460

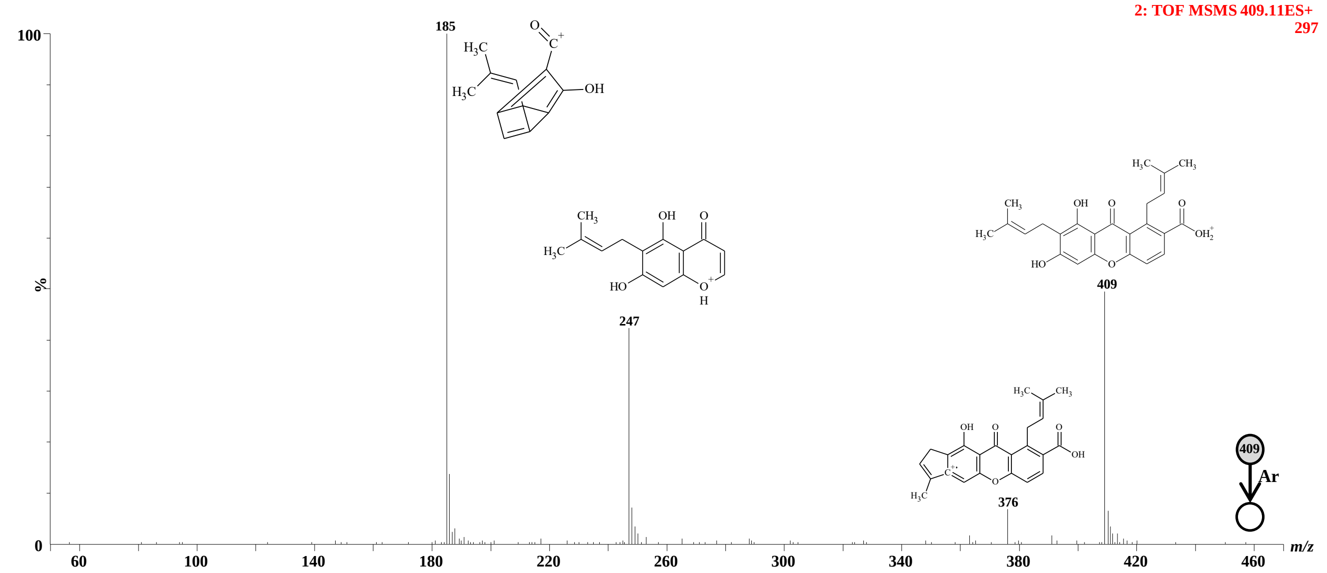
2: TOF MSMS 409.11ES+
297
100
%
0
185
247
409
376
409
Ar
m/z
60
100
140
180
220
260
300
340
380
420
460

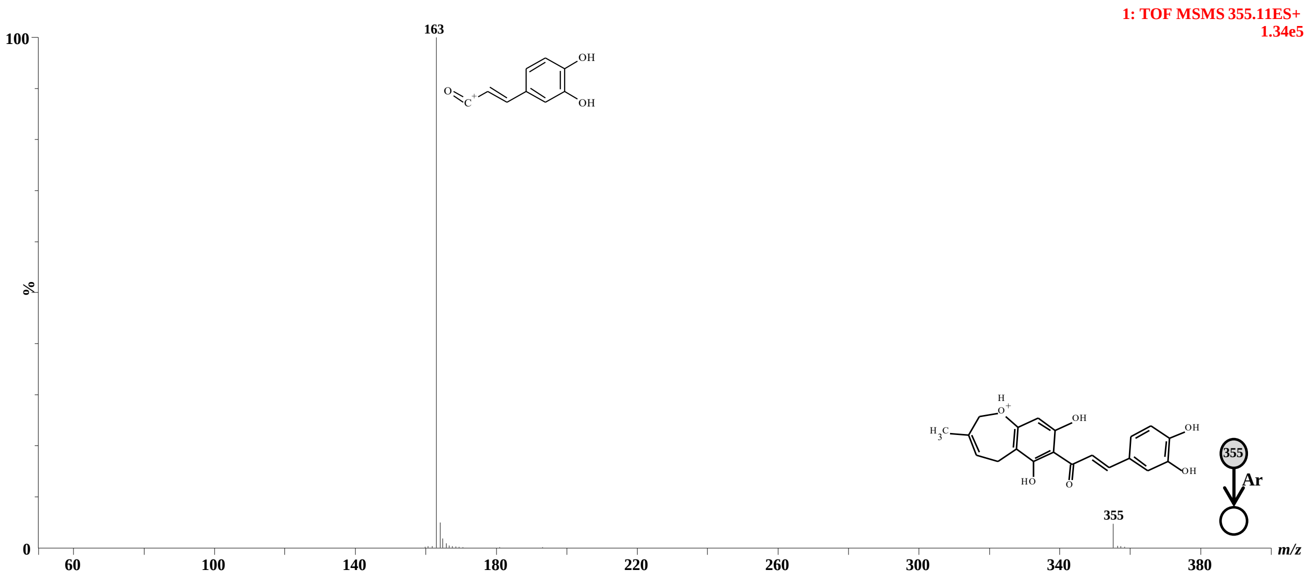
1: TOF MSMS 355.11ES+
1.34e5
163
355
%
m/z
Ar
355

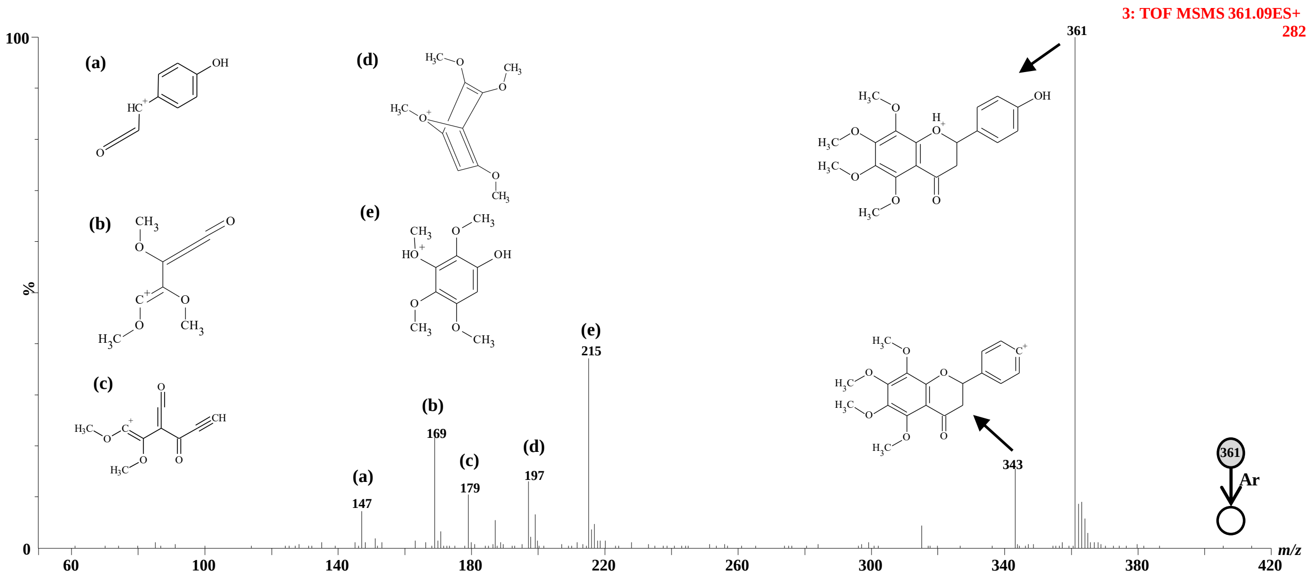
3: TOF MSMS 361.09ES+
282
(a) 147
(b) 169
(c) 179
(d) 197
(e) 215
343
361
%
m/z
Ar
361

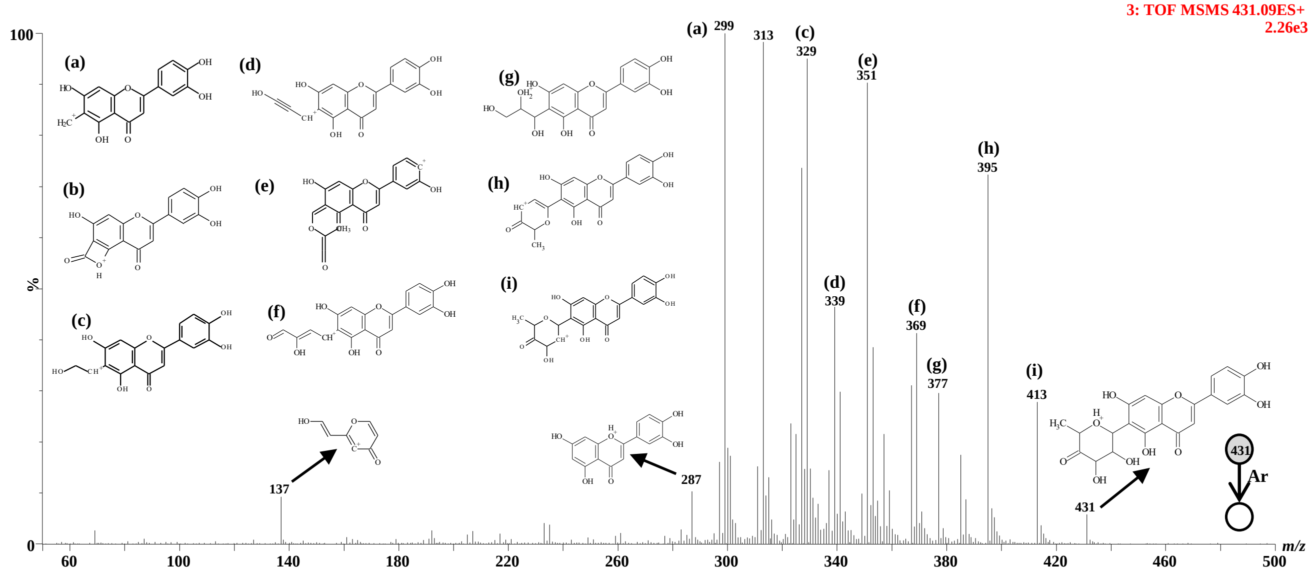
3: TOF MSMS 431.09ES+
2.26e3
(a) 299
313
(c) 329
(e) 351
(d) 339
(f) 369
(g) 377
(h) 395
(i) 413
137
287
431
%
m/z
Ar
431

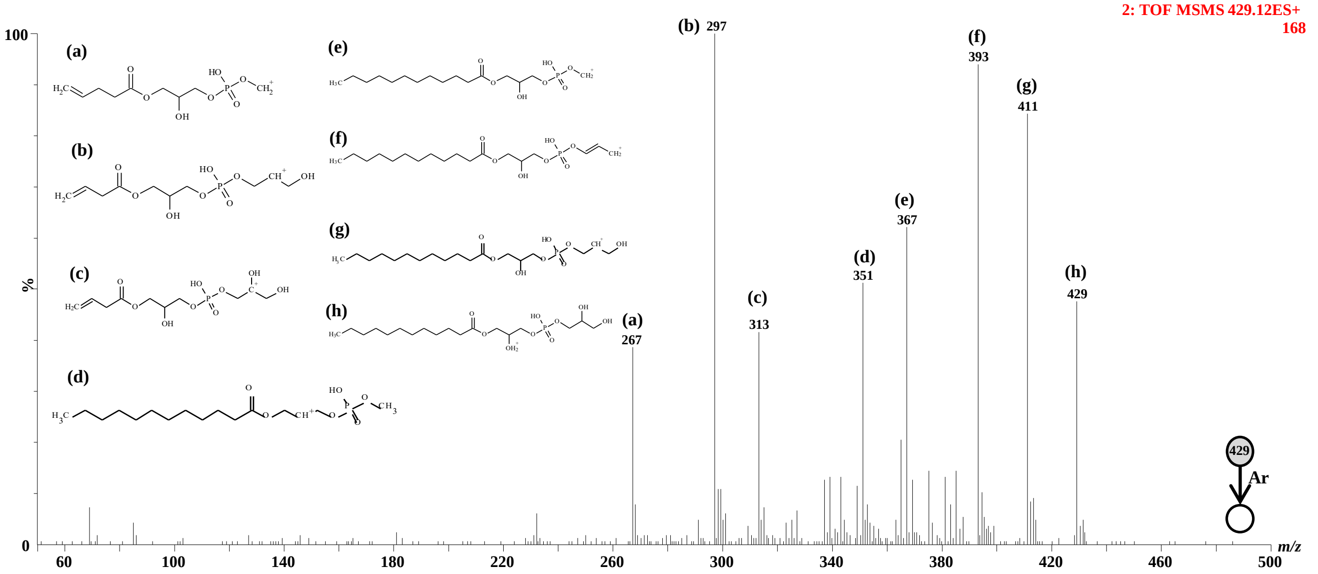
2: TOF MSMS 429.12ES+
168
(a) 267
(b) 297
(c) 313
(d) 351
(e) 367
(f) 393
(g) 411
(h) 429
429
Ar
%
m/z

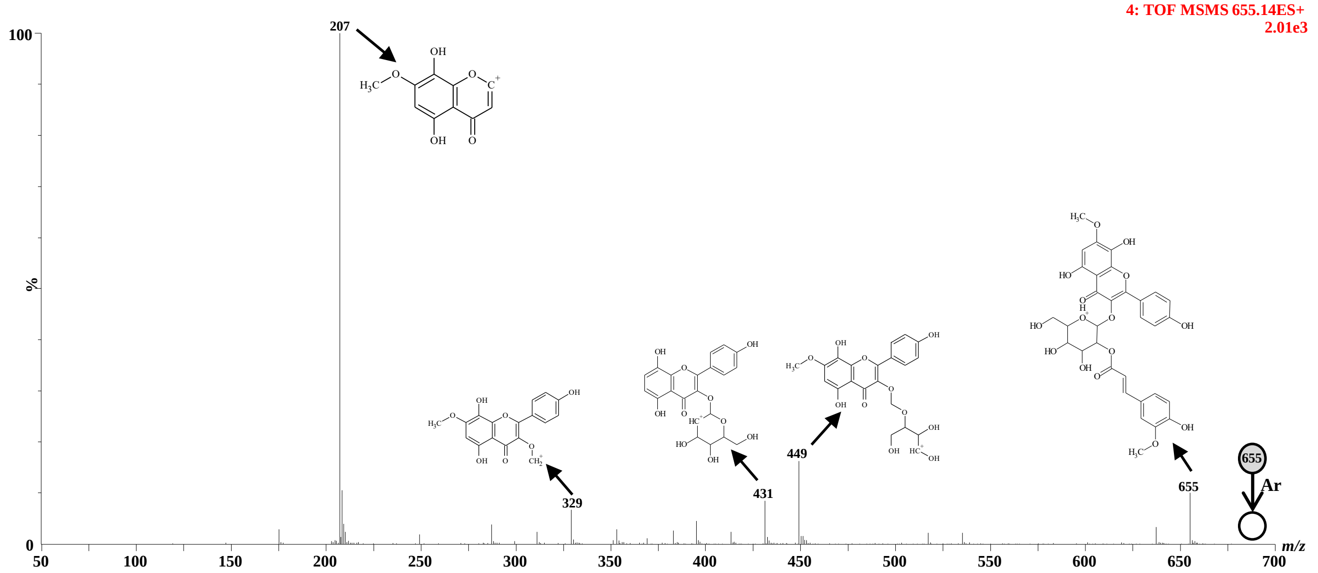
4: TOF MSMS 655.14ES+
2.01e3
207
329
431
449
655
Ar
%
m/z

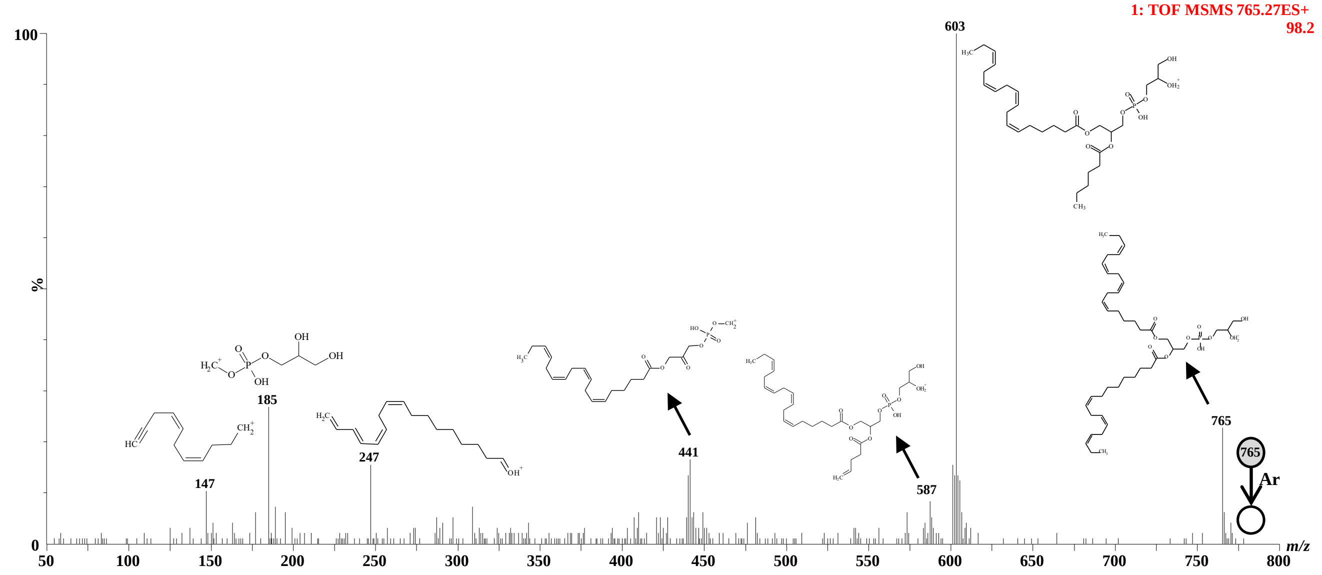
1: TOF MSMS 765.27ES+
98.2
603
147
185
247
441
587
765
Ar
%
m/z

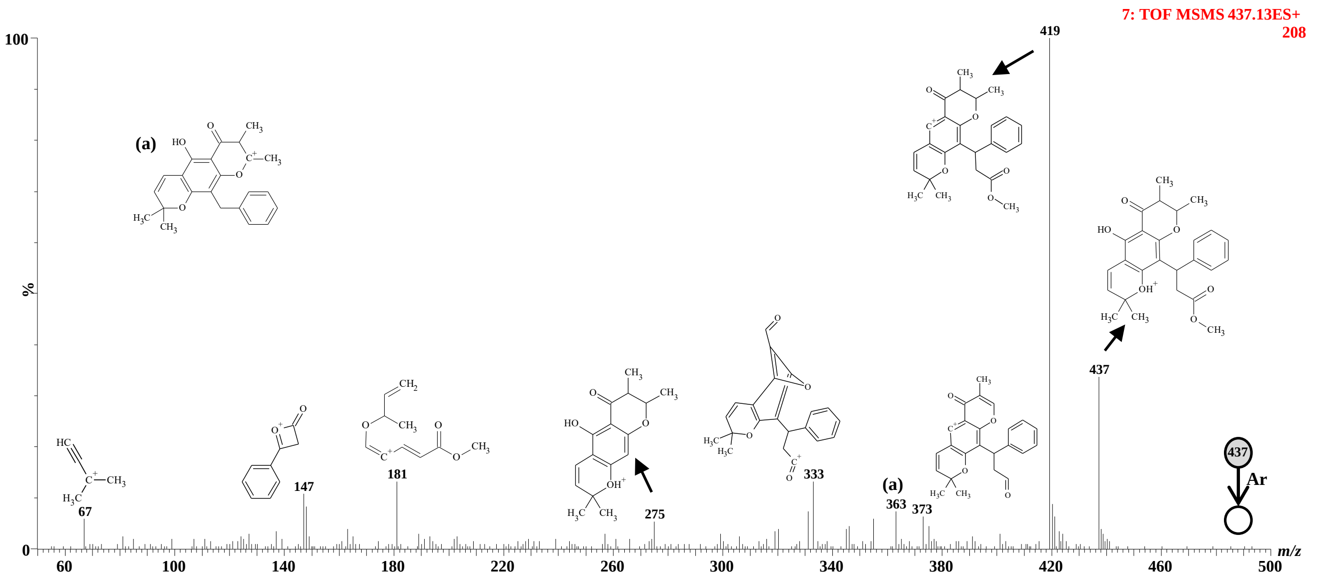
7: TOF MSMS 437.13ES+
208
100
%
0
(a)
67
147
181
275
333
(a) 363
373
419
437
Ar
m/z
60 100 140 180 220 260 300 340 380 420 460 500

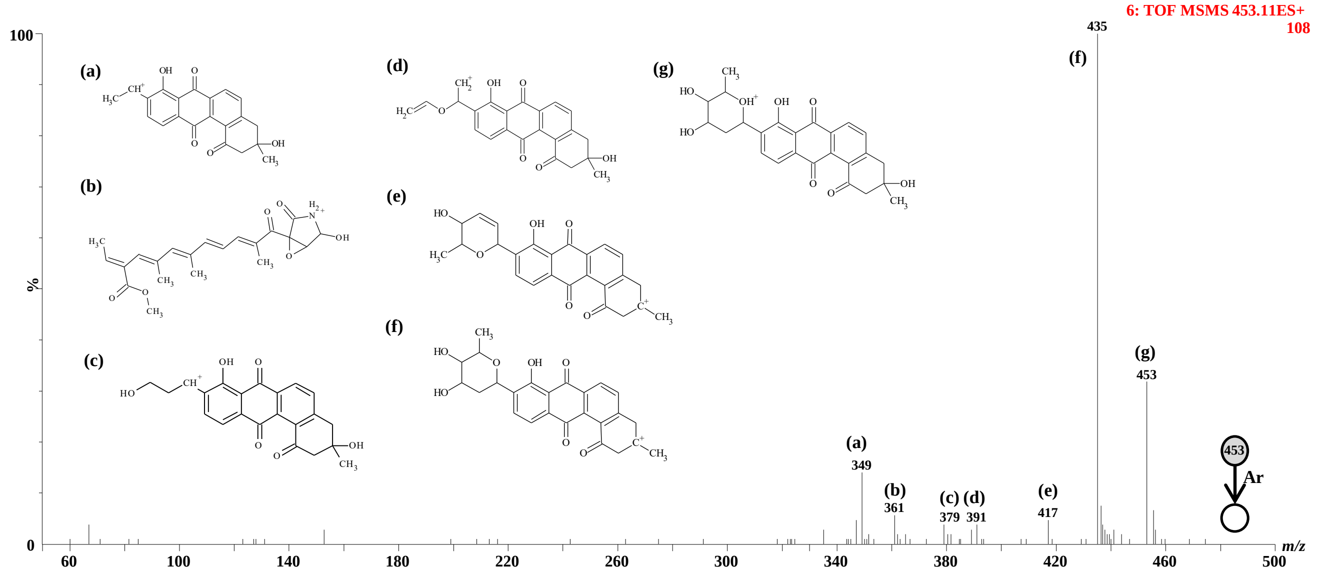
6: TOF MSMS 453.11ES+
108
100
%
0
(a)
(b)
(c)
(d)
(e)
(f)
(g)
(a) 349
(b) 361
(c) 379
(d) 391
(e) 417
(f) 435
(g) 453
Ar
m/z
60 100 140 180 220 260 300 340 380 420 460 500

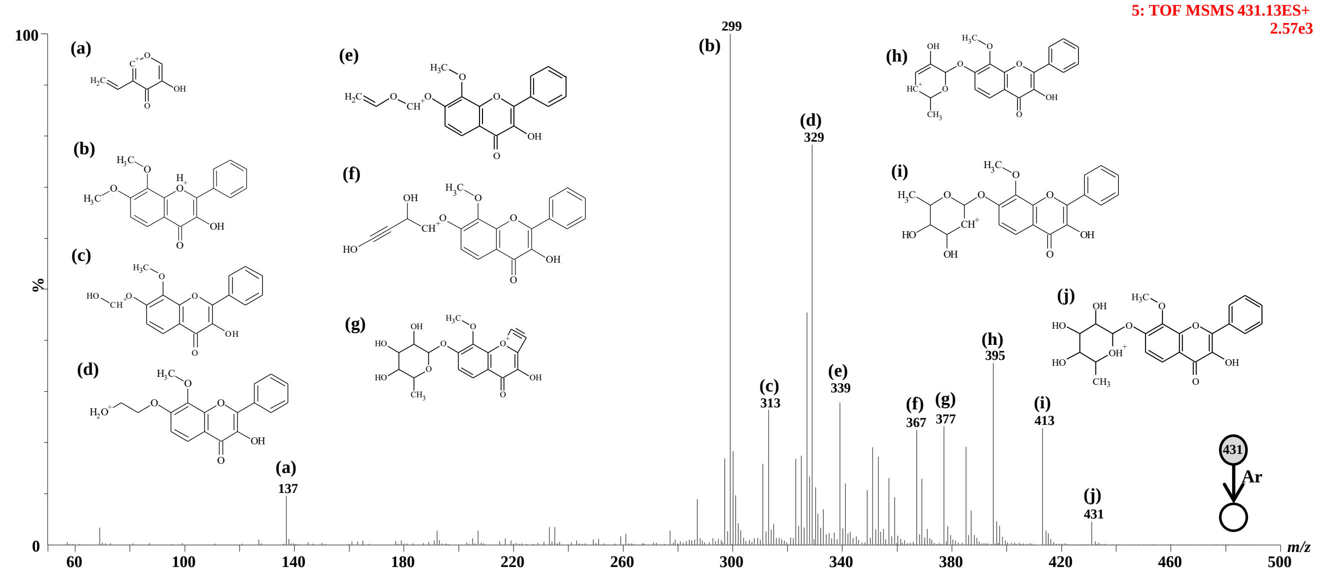
5: TOF MSMS 431.13ES+
2.57e3
100
%
0
(a)
(b)
(c)
(d)
(e)
(f)
(g)
(h)
(i)
(j)
(a) 137
(b) 299
(c) 313
(d) 329
(e) 339
(f) 367
(g) 377
(h) 395
(i) 413
(j) 431
Ar
m/z
60 100 140 180 220 260 300 340 380 420 460 500

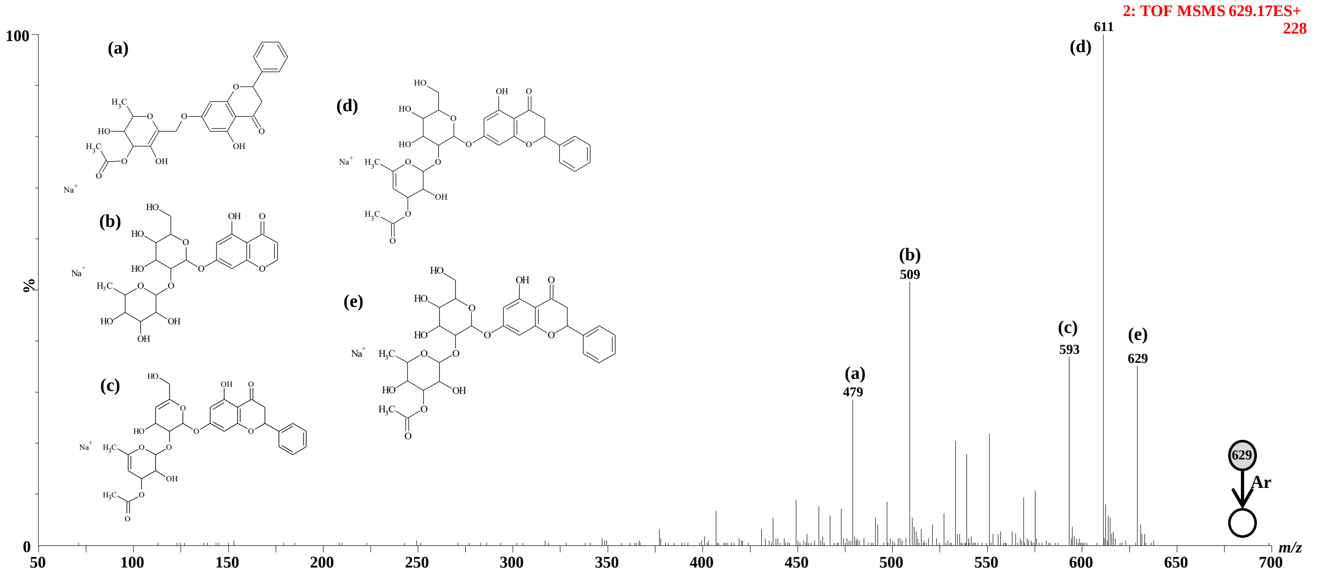
2: TOF MSMS 629.17ES+
228
611
509
593
629
479
629
Ar
m/z

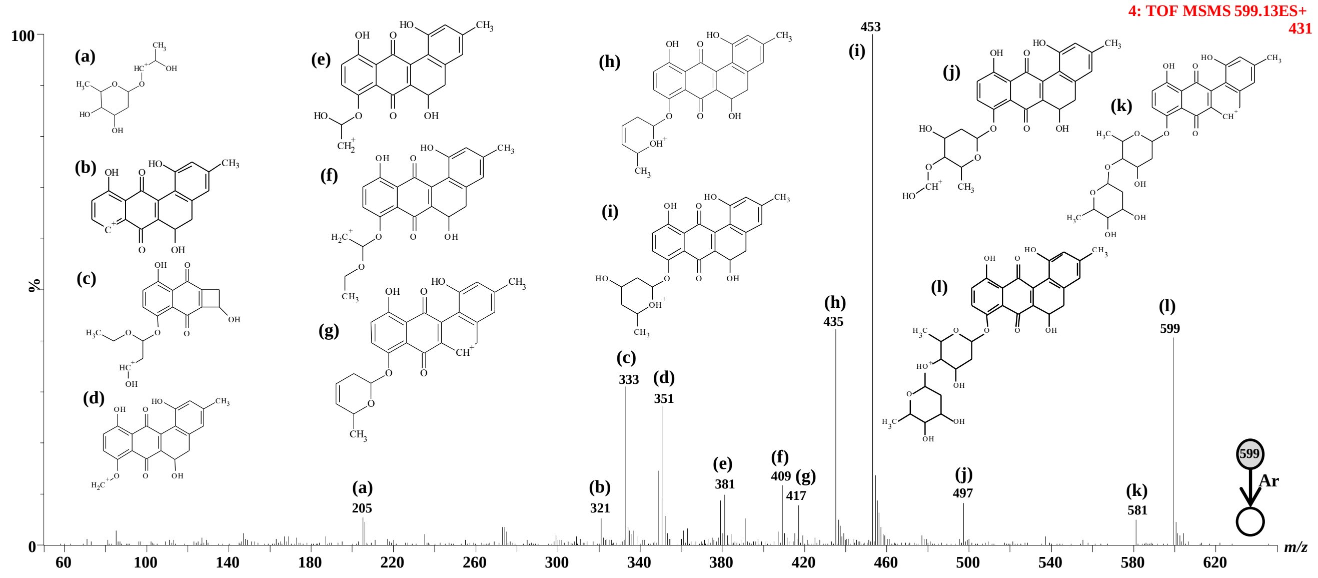
4: TOF MSMS 599.13ES+
431
453
435
333
351
381
409
417
205
321
497
581
599
Ar
m/z

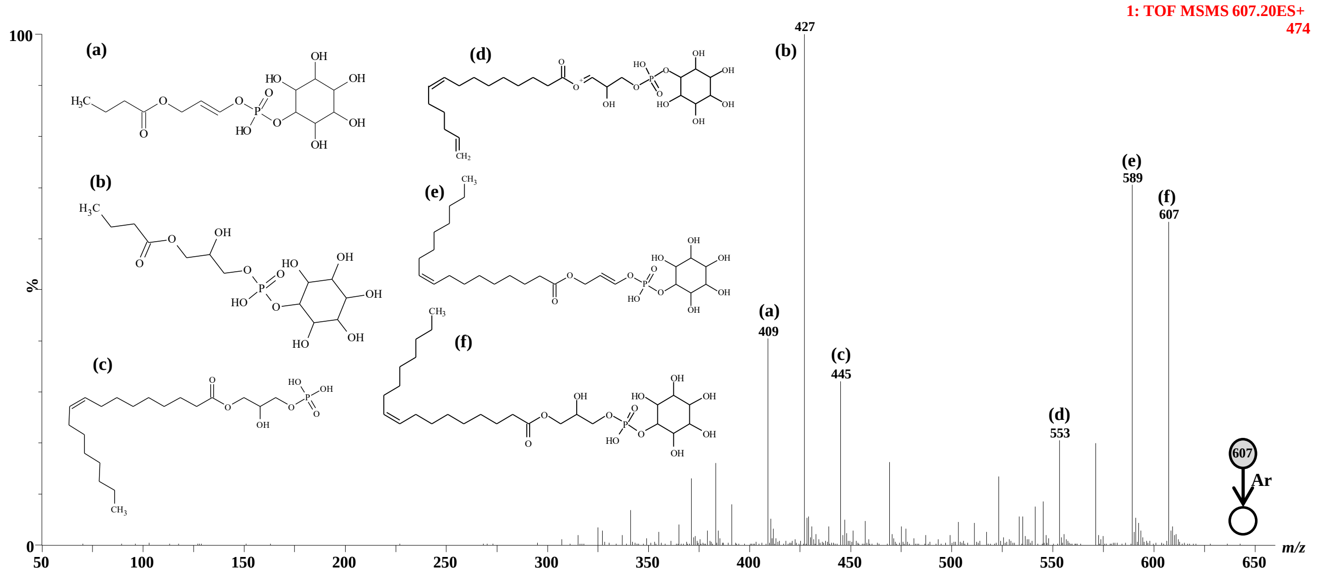
1: TOF MSMS 607.20ES+
474
427
589
607
409
445
553
607
Ar
m/z

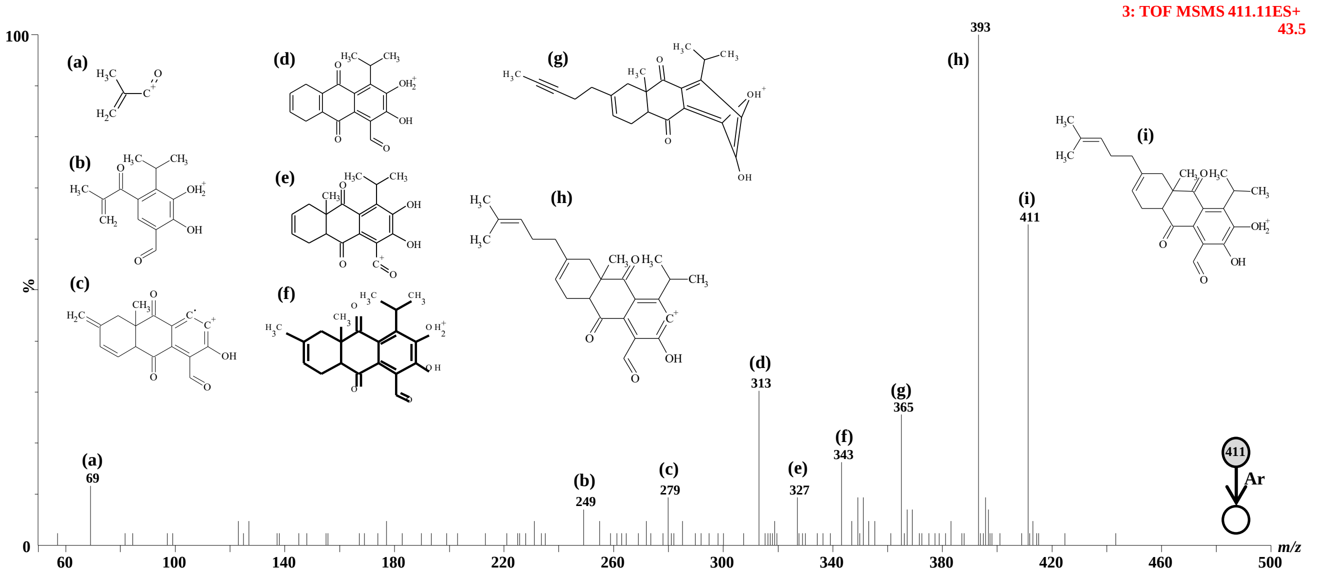
3: TOF MSMS 411.11ES+
43.5
100
%
0
(a) 69
(b) 249
(c) 279
(d) 313
(e) 327
(f) 343
(g) 365
(h) 393
(i) 411
411
Ar
60
100
140
180
220
260
300
340
380
420
460
500
m/z
