# Supplementary material for: Metabolome Dynamics of Smutted Sugarcane Reveals Mechanisms Involved in Disease Progression and Whip Emission
Source: Front Plant Sci. 2017 May 31;8:882. doi: 10.3389/fpls.2017.00882 (PMC5450380; doi:10.3389/fpls.2017.00882)
Supplement: Supplementary file 5 [file Presentation3.PDF]

Supporting Information File S3. Fragmentation patterns of selected metabolites identified as responsive to smut pathogen in LC-ESI-MS/MS negative ionization mode. Metabolites were using ACD/Labs software to theoretical fragmentation of structures from Metlin database (https://metlin.scripps.edu/index.php).

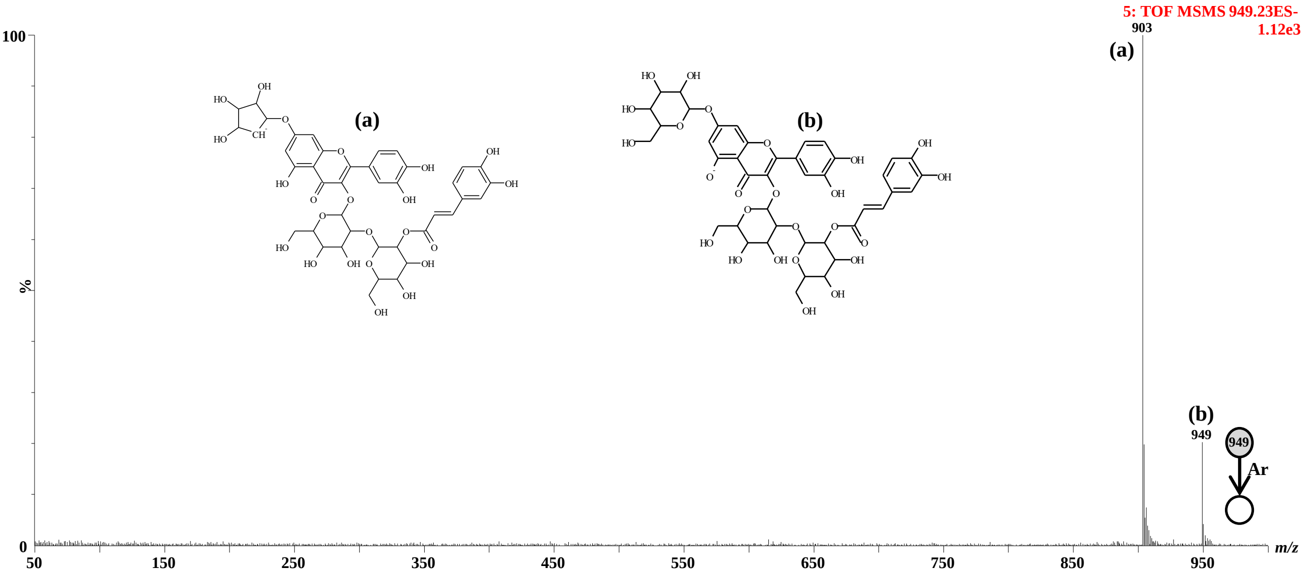

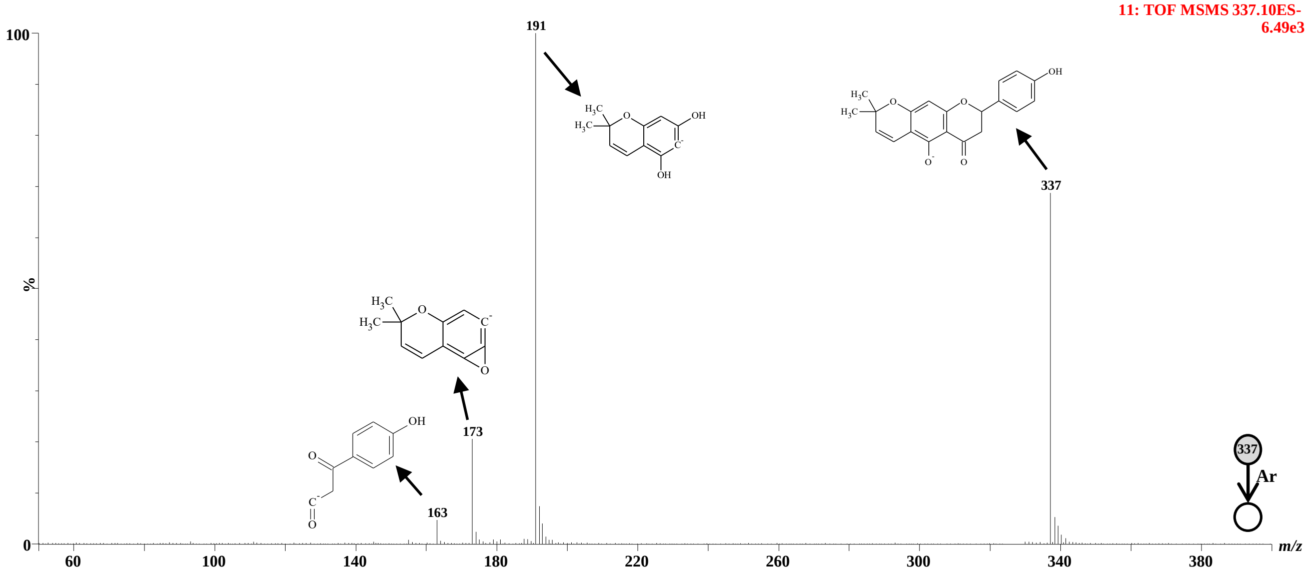

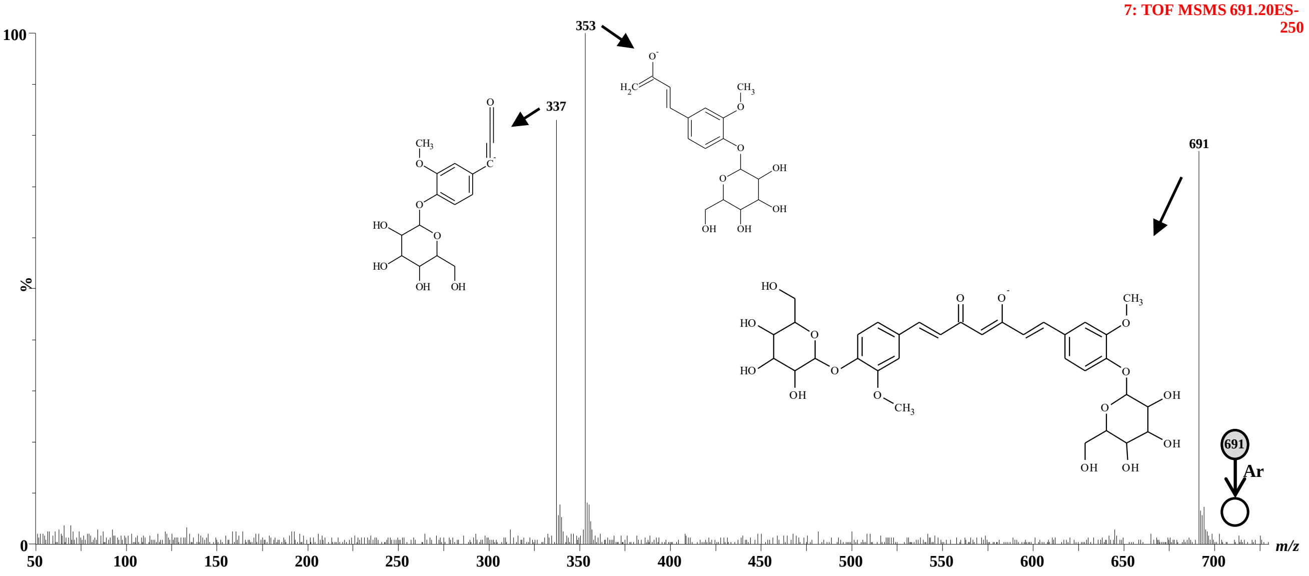

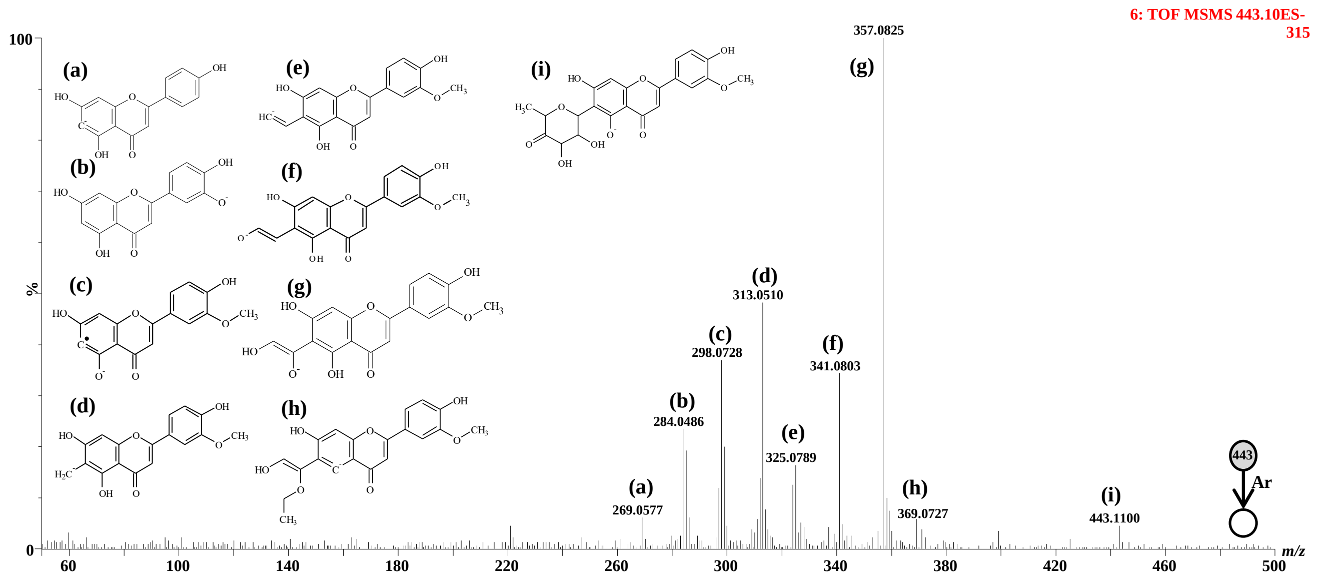
6: TOF MSMS 443.10ES-
315
100
%
0
(a)
(b)
(c)
(d)
(e)
(f)
(g)
(h)
(i)
357.0825
313.0510
298.0728
341.0803
284.0486
325.0789
269.0577
369.0727
443.1100
443
Ar
m/z
60
100
140
180
220
260
300
340
380
420
460
500

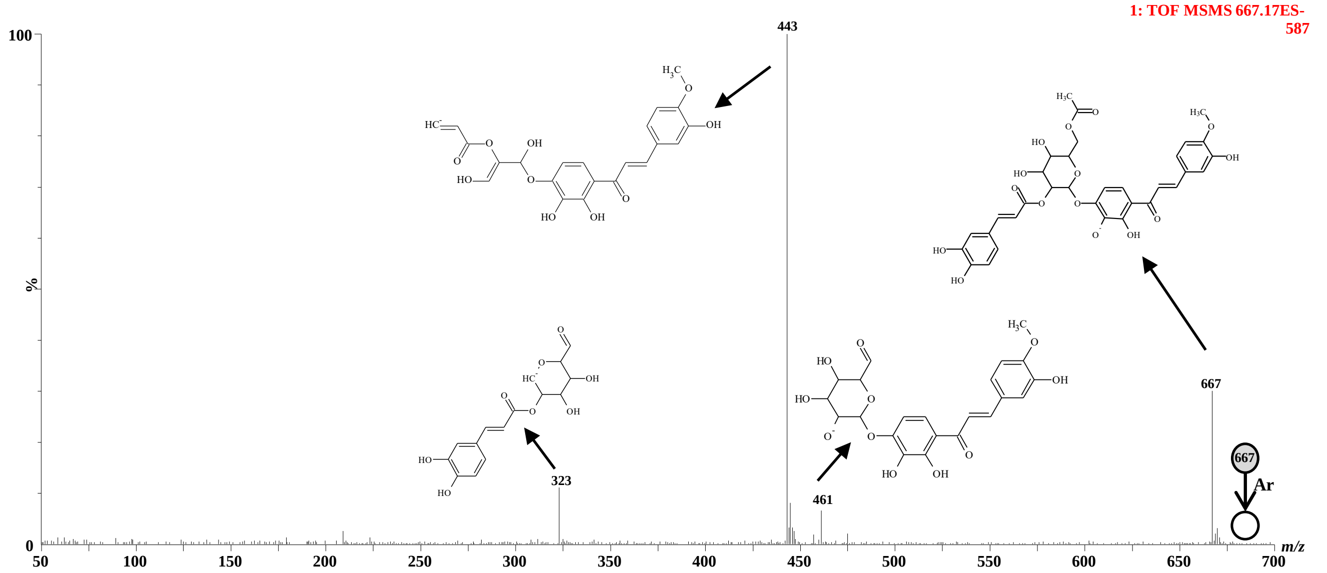
1: TOF MSMS 667.17ES-
587
100
%
0
443
667
323
461
667
Ar
m/z
50
100
150
200
250
300
350
400
450
500
550
600
650
700

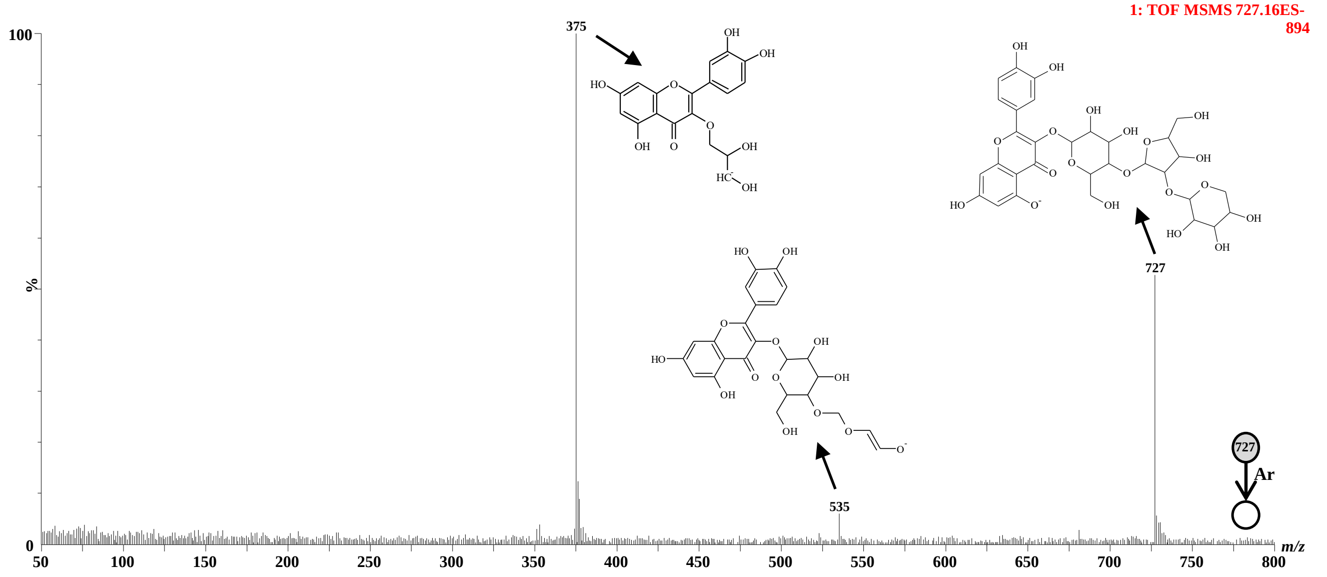
1: TOF MSMS 727.16ES-
894
100
%
0
375
727
535
727
Ar
m/z
50
100
150
200
250
300
350
400
450
500
550
600
650
700
750
800
