# Supplementary material for: Metabolome Dynamics of Smutted Sugarcane Reveals Mechanisms Involved in Disease Progression and Whip Emission
Source: Front Plant Sci. 2017 May 31;8:882. doi: 10.3389/fpls.2017.00882 (PMC5450380; doi:10.3389/fpls.2017.00882)

Supporting Information File S4a: *S. scitamineum* gene clusters codifying to secondary metabolites, according to antiSMASH analysis.

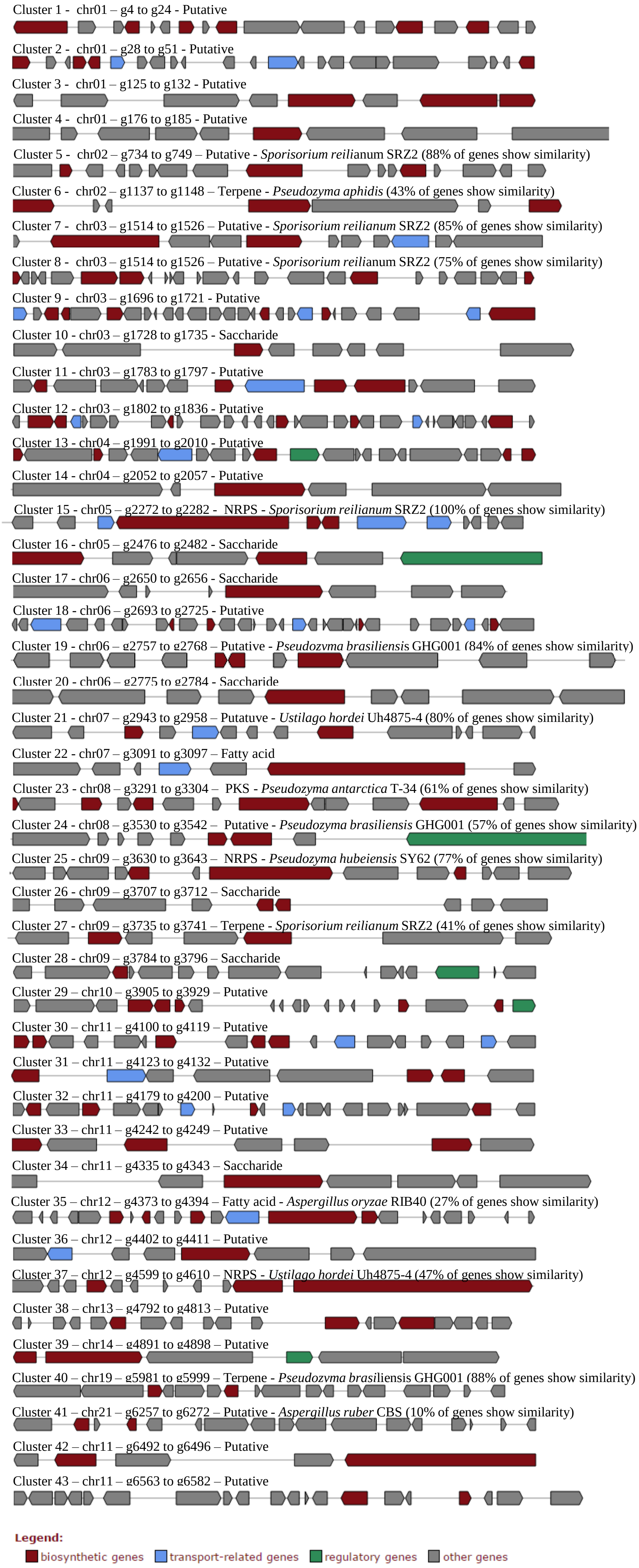

Supporting Information File S4b: Expression profile of *S. scitamineum* gene clusters codifying to secondary metabolites. Normalized number of reads was used. Data source: Taniguti et al., 2015.

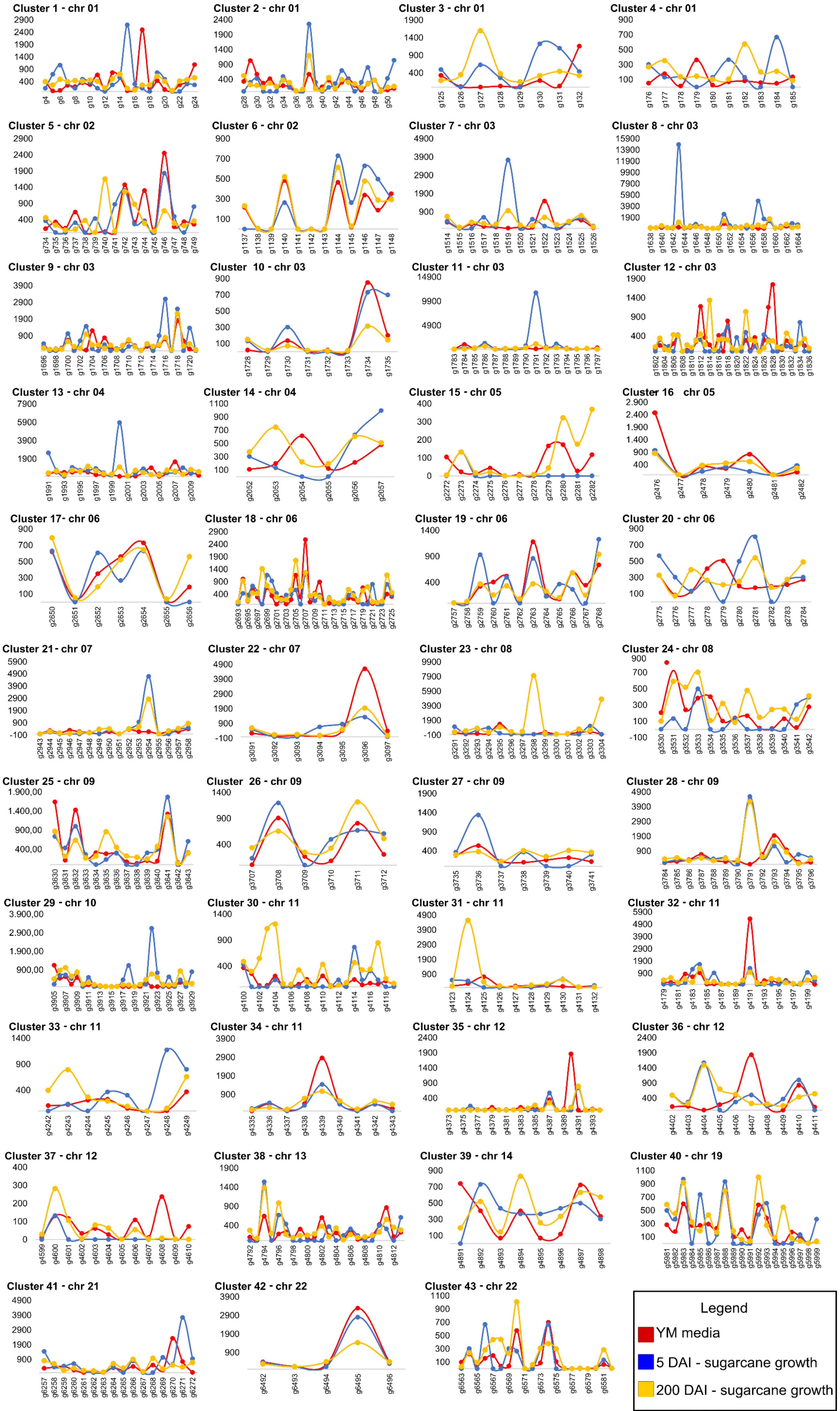

Supplement: Supplementary file 6 [file Presentation4.PDF]
